# Effect of Inoculation with *Lentilactobacillus buchneri* and *Lacticaseibacillus paracasei* on the Maize Silage Volatilome: The Advantages of Advanced 2D-Chromatographic Fingerprinting Approaches

**DOI:** 10.1021/acs.jafc.2c03652

**Published:** 2022-09-14

**Authors:** Simone Squara, Francesco Ferrero, Ernesto Tabacco, Chiara Cordero, Giorgio Borreani

**Affiliations:** † Dipartimento di Scienza e Tecnologia del Farmaco, University of Turin, Turin 10124, Italy; ‡ Department of Agriculture, Forest and Food Sciences, University of Turin, Grugliasco 10124, TO, Italy

**Keywords:** comprehensive two-dimensional gas chromatography, combined untargeted and targeted (UT) fingerprinting, maize silage, volatile organic compounds, fermentative profile, LAB inocula, aerobic stability, yeast activity inhibition

## Abstract

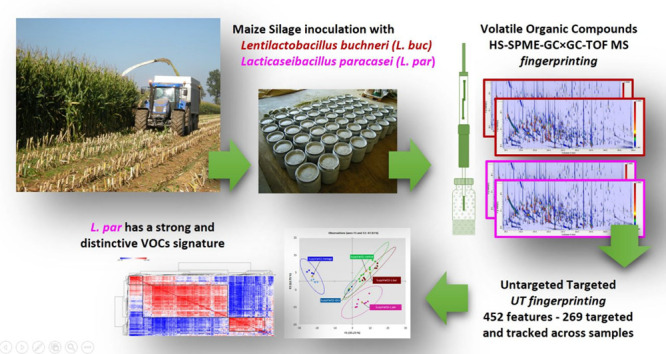

In this study, the complex volatilome of maize silage samples conserved for 229 d, inoculated with *Lentilactobacillus buchneri* (*Lbuc*) and *Lacticaseibacillus paracasei* (*Lpar*), is explored by means of advanced fingerprinting methodologies based on comprehensive two-dimensional gas chromatography and time-of-flight mass spectrometry. The combined untargeted and targeted (UT) fingerprinting strategy covers 452 features, 269 of which were putatively identified and assigned within their characteristic classes. The high amounts of short-chain free fatty acids and alcohols were produced by fermentation and led to a large number of esters. The impact of *Lbuc* fermentation was not clearly distinguishable from the control samples; however, *Lpar* had a strong and distinctive signature that was dominated by propionic acid and 1-propanol characteristic volatiles. The approach provides a better understanding of silage stabilization mechanisms against the degradative action of yeasts and molds during the exposure of silage to air.

## Introduction

Improving silage fermentation and aerobic stability through the use of bacterial inocula is a widely studied area.^1^ The aerobic stability of silage during the feed-out phase is one of the most frequently requested characteristics of silage at a farm level to reduce the risk of aerobic deterioration.^2^ Among the lactic acid bacteria (LAB) used to improve the aerobic stability of silages, heterofermentative *Lentilactobacillus buchneri* strains are the most successful.^1,3^ Their action arises from their capability of modifying fermentative patterns by partially converting lactic acid into acetic acid and 1,2-propanediol.^4^ Several studies have only attributed the improvement of aerobic stability from the use of *Len. buchneri* to an increase in acetic acid,^3,5^ even though, in several cases, the acetic acid content has not been able to fully explain the yeast reduction and the increase in aerobic stability.^6^ Silage fermentation is a complex process, and a large number of compounds is generated.^7^ Silage volatile organic compounds (VOCs) represent a complex fraction that includes both native components of unfermented silage, derived from primary and secondary/specialized plant metabolisms, and volatile metabolites produced by the metabolic activity of bacteria and yeasts during fermentation.^8^ Moreover, certain abiotic conditions (pH and temperature) can promote the formation of additional components, as in the case of ester derivatives.^9^

The investigation of silage VOCs was first reported by Krizsan et al. (2007) who identified and quantified 13 esters, five aldehydes, three alcohols, and one sulfur derivative that showed effects on the voluntary intake of growing steers. The role of silages in contributing to the atmospheric emission of VOCs was then studied.^9,10^ Around 80 compounds of acids, ketones, aldehydes, alcohols, esters, and other groups were identified in maize, alfalfa, and cereal silages.^10^

The use of comprehensive two-dimensional (2D) gas chromatography (GC × GC) coupled with time-of-flight mass spectrometry (TOF MS), can greatly improve the knowledge about the quali-/quantitative composition of silage VOCs and add further information to better understand the complex phenomena behind aerobic stability, bacteria and yeast metabolic activity, synergies, and cross-interactions. The improved separation power of GC × GC, compared to one-dimensional (1D) GC, accompanied by the logical retention patterns of chemically related compounds, and specialized data processing techniques, make GC × GC-TOF MS the most suitable platform for an accurate exploration of complex volatile fractions (i.e., the volatilome).^11−13^ When the fraction under study poses challenges, because of the large dynamic range of concentrations, and consists of analytes with a wide polarity range within a relatively narrow volatility interval, chromatographic resolution and efficiency are fundamental to achieve appropriate performances. Moreover, when an investigation is directed toward all the detectable components, untargeted approaches represent the ideal strategy, since they are not biased by previous knowledge of specific markers or target analytes and lead to a comprehensive understanding of the phenomena.

In this study, state-of-the art GC × GC-TOFMS, combined with automated headspace solid-phase microextraction (HS-SPME), has been adopted for the first time to capture the complexity and chemical dimensionality of the maize silage volatilome. The effects of silage inocula, based on commercial strains of *Len. buchneri* and a new strain of *Lacticaseibacillus paracasei*, on the volatilome of maize silage harvested at two different dry matter (DM) contents, have been studied to investigate the role of some VOCs in improving aerobic stability after silo opening. Moreover, in order to take a step forward in the understanding of the biological phenomena behind silage fermentation, comprehensive chromatographic fingerprinting,^14^ covering untargeted and targeted components, has been applied. The chemical signatures of the volatilome have been explored with unsupervised and supervised chemometrics to highlight the interaction of *Len. buchneri* and *Lcb. paracasei* with the epiphytic microorganisms present on the forage at harvesting.

## Materials and Methods

### Chemicals

The pure α/β-thujone and methyl 2-octynoate reference standards, used as internal standards (ISs), the *n-*alkanes (from *n*-C9 to *n*-C25), used for linear retention indice (I^T^s) calibration, and the solvents (cyclohexane, toluene, and dibutyl phthalate—99% of purity) used in the analyses were all obtained from Merck.

### Fermented Maize Silage Samples

The trial was performed at the experimental farm of the University of Turin in the western Po plain, northern Italy (44°53′N, 7°41′E, altitude 232 m a.s.l.). Maize (P1547W, Pioneer Hi-Bred Italia Srl) was seeded on two different dates (2020-04-16 and 2020-05-25) in order to contemporary harvest two whole crops with different DM contents [LOW (32% DM) and HIGH (39% DM)]. The forage was directly harvested as a chopped whole crop using a precision forage harvester (Claas Jaguar 970 equipped with a New Holland 350W forage harvester head) at a 15 mm chopping length. The field was divided into three blocks. The chopped material from each block was divided into two representative 80 kg piles (one for each treatment) for each DM content. The piles were either not treated, and used as a negative control (CON), or treated with *Lentilactobacillus buchneri* and *Lactiplantibacillus plantarum* (Corteva Agriscience, Johnston, Iowa, USA) at a theoretical application rate of 1.1 × 10^5^ cfu g^–1^ fresh matter FM (*Lbuc*) or *Lacticaseibacillus paracasei* (UNITO 012, University of Turin, Italy) at a theoretical application rate of 1 × 10^6^ cfu g^–1^ FM (*Lpar*). A hand sprayer was used to uniformly spray the inocula onto the forage, which was continuously hand-mixed. The fresh forages were sampled (one sample from each pile) prior to ensiling and after treatment with the inocula. The forages were hand-packed into 20 L plastic silos equipped with a lid that only enabled gas release, and the final average packing density was 674 ± 31 and 584 ± 32 kg FM m^–3^, for LOW and HIGH, respectively. All the laboratory silos were filled within 3 h. The silos were weighed, conserved at ambient temperature (20 ± 1 °C), and opened after 229 d of anaerobic conservation. At opening, each silo was again weighed, and the content was mixed thoroughly and subsampled to determine the DM content, chemical composition, fermentation profile, microbial counts, and aerobic stability.

The weight losses due to fermentation were calculated as the difference between the weight of the forage placed in each plastic silos at ensiling and the weight of the silage at the end of conservation, and they were expressed as the percentage of the amount of DM ensiled in each plastic silo.

After sampling, the silages were subjected to an aerobic stability test. Aerobic stability was determined by monitoring the temperature increases due to the microbial activity of the samples exposed to air. About three kilograms of each silo was allowed to aerobically deteriorate in a controlled temperature room (20 ± 1 °C) in 17 L polystyrene boxes (290 mm diameter and 260 mm height) for 14 d. A single layer of aluminum cooking foil was placed over each box to prevent drying and dust contamination but also to allow air to penetrate. The temperatures of the room and of the silage were measured each hour by a data logger. Aerobic stability was defined as the number of hours the silage remained stable before rising more than 2 °C above room temperature as reported by Kleinschmit and Kung.^3^

### Sample Preparation and Analyses

Each of the pre-ensiled herbages and the silages were split into five subsamples of about 500 g.

The first subsample was analyzed immediately, for the DM content, by oven drying at 80 °C for 24 h. The dry matter was corrected, according to Porter and Murray,^15^ to consider the volatile compound losses that can take place at 80 °C.

The second subsample was used to determine the water activity (*a*_w_), pH, nitrate (NO_3_), and the buffering capacity. The water activity was measured at 25 °C on a fresh sample using an AquaLab Series 3TE (Decagon Devices Inc.), which adopts the chilled-mirror dew point technique. The fresh forage was extracted for pH and nitrate determination, using a Stomacher blender (Seward Ltd.), for 4 min in distilled water at a 9:1 water-to-sample material (fresh weight) ratio. The total nitrate concentration was determined in the water extract, through semiquantitative analysis, using Merckoquant test strips (Merck; detection limit 100 mg NO_3_ kg^–1^ DM). The pH was determined using a specific electrode (DL21 Titrator, Mettler Toledo, with electrode Liq-Glass 238000, Hamilton, Agrate Brianza, IT). The buffering capacity was determined in the water extract, as described by Playne and McDonald.^16^

A third fresh subsample was extracted, using a Stomacher blender, for 4 min in H_2_SO_4_ 0.05 mol L^–1^ at a 4:1 acid-to-sample material (fresh weight) ratio. An aliquot of 40 mL of silage acid extract was filtered with a 0.20 μm syringe filter and used for quantification of the fermentation products. The lactic and monocarboxylic acids (acetic, propionic, and butyric acids) in the acid extract were determined using high-performance liquid chromatography (HPLC, Agilent Technologies, 1200 Series).^17^ Ethanol and 1,2-propanediol were determined using HPLC, coupled with a refractive index detector, on a Aminex HPX-87H column (Bio-Rad Laboratories).

The fourth fresh subsample was used for the microbial analyses. In order to conduct the microbial counts, a 30 g sample was transferred to a sterile homogenization bag, suspended 1:9 w/v in a peptone salt solution (1 g of bacteriological peptone and 9 g of sodium chloride per liter), and homogenized for 4 min in a laboratory Stomacher blender (Seward Ltd.). Serial dilutions were prepared, and the yeast and mold numbers were determined using the pour plate technique with 40.0 g L^–1^ of Yeast Extract Glucose Chloramphenicol Agar (YGC agar, DIFCO) after incubation at 25 °C for 3 and 5 d for yeast and mold, respectively. The yeast and mold colony forming units (cfu) were enumerated separately, according to their macromorphological features, on plates that yielded 1–100 cfu. The LAB were determined on MRS agar with added natamycin (0.25 g L^–1^), by incubating the Petri plates at 30 °C for 3 d in anaerobic jars with a gas generating system (AnaeroGenTM, Thermo Fisher Scientific). Since LAB are facultative anaerobe bacteria, anaerobic incubation was chosen to improve the selectivity of the media against *Bacillus* spp.

The fifth fresh subsample, used for volatilome analysis, was stored in a plastic, phthalate-free container, immediately frozen at −80 °C, and kept refrigerated until analysis.

The main chemical and microbial characteristics of whole crop corn (WCC), harvested at LOW and HIGH DM contents prior to ensiling and after 229 d of fermentation, are reported in Table 1.

**Table 1:** Main Chemical and Microbial Characteristics of Whole Crop Corn (WCC) Harvested at LOW and HIGH DM Content Prior to Ensiling and after 229 d of Fermentation of Treated or Not Treated with Lactic Acid Bacteria Inocula (*Lbuc* and *Lpar*)

	at ensiling (time 0 d)				silage (time 229 d) general means								
	LOW	HIGH	SEM	*P*-value	LOW	HIGH	CON	Lbuc	Lpar	SEM	*D*	*L*	*D* × *L*
DM (%)	32.3	40.9	1.61	<0.001	32.5	38.6	36.7	35.2	34.9	0.794	<0.001	0.051	0.798
pH	5.88	5.98	0.023	0.150	3.96	3.87	3.67^c^	3.81^b^	4.27^a^	0.066	0.046	0.001	0.347
buffering capacity (meq kg^–1^ DM)	56.9	51.1	2.90	0.484									
nitrate (mg kg^–1^ DM)	1397	354	226	0.003	682	<100	498	525	<100				
lactic acid bacteria (log cfu g^–1^)	7.30	8.58	0.211	<0.001	7.49	7.73	6.85^b^	7.95^a^	8.03^a^	0.187	0.242	0.003	0.331
yeast (log cfu g^–1^)	6.79	7.67	0.114	<0.001	<1.00	1.48	1.28	1.42	<1.00	0.215	0.032	0.097	0.209
mold (log cfu g^–1^)	6.33	7.21	0.138	<0.001	1.03	<1.00	<1.00	1.12	<1.00				
enterobacteria (log cfu g^–1^)	6.80	7.69	0.181	0.006	<1.00	<1.00	<1.00	<1.00	<1.00				
DM losses (%)					3.77	2.93	2.53^c^	3.21^b^	4.30^a^	0.219	<0.001	<0.001	0.766
aerobic stability (h)					619	441	340^b^	237^b^	1013^a^	93.16	0.039	<0.001	0.362
lactic to acetic ratio					1.92	2.79	4.15^a^	2.35^b^	0.57^b^	0.393	0.006	<0.001	0.083
lactic acid (g kg^–1^ DM)					43.12	44.06	59.58^a^	50.20^a^	20.99^b^	4.271	0.800	<0.001	0.908
acetic acid (g kg^–1^ DM)					29.05	21.61	15.18^c^	22.37^b^	38.43^a^	2.603	<0.001	<0.001	0.465
butyric acid (g kg^–1^ DM)					<0.1	<0.1	<0.1	<0.1	<0.1				
propionic acid (g kg^–1^ DM)					4.76	3.23	0.15	2.08	9.76				
1-propanol (g k^–1^g DM)					3.83	2.61	<0.1	0.64	9.03				
1,2-propanediol (g kg^–1^ DM)					1.76	2.83	0.96	4.99	0.93				
ethanol (g kg^–1^ DM)					13.59	13.10	11.75^b^	13.50^ab^	14.77^a^	0.416	0.461	0.008	0.649

cfu = colony forming unit; DM = dry matter; SEM = standard error of the mean.

^a−c^ Means within a row with different superscripts differ (P < 0.05).

### Headspace Solid-Phase Microextraction: Devices and Conditions

The volatile organic compounds from the silage samples were sampled by means of HS-SPME using an SPR Auto sampler for GC (SepSolve-Analytical). A divinylbenzene/carboxen/polydimethylsiloxane (DVB/CAR/PDMS) fiber (*d*_f_ 50/30 μm; 2 cm length) from Merck was chosen because of the possibility of combining sorption and adsorption mechanisms with components covering a large polarity range. The SPME fiber was conditioned before use, as recommended by the manufacturer. The ISs (α/β-thujone and methyl 2-octynoate) were preloaded onto the SPME device^18,19^ by sampling from a 20 mL headspace vial containing a 5.0 μL aliquot of the ISs solution (100 mg L^–1^) prepared in dibutyl phthalate as a solvent. The ISs were equilibrated at 40 °C, and the SPME device was exposed to the ISs HS for 5 min. ISs were used for validation purposes (method precision and repeatability) and to normalize the analytes’ absolute responses (i.e., *% normalized response*).

Sampling was carried out on 1.00 ± 0.10 g of finely ground silage, precisely weighed, placed in 20 mL headspace vials, and kept at 40 °C for 50 min under constant agitation. The amount of the sample, the sampling temperature, and time were optimized after preliminary experiments (data not shown). The final conditions were set to obtain the maximum extraction efficiency in a reasonable sampling time (according to the duration of the analytical run) and at a temperature where the formation of artifacts and side-reactions was minimized (i.e., 40 °C).

After extraction, the SPME device was automatically transferred to the split/splitless injection port of the GC × GC system and kept at 250 °C, and thermal desorption was then run for 5 min. The samples were analyzed in duplicate and randomly distributed over one week of measurements.

### GC × GC-TOF MS with Loop-type Thermal Modulation: Instrument Setup and Conditions

Comprehensive two-dimensional GC analyses were carried out with an Agilent 7890B GC chromatograph (Agilent Technologies) coupled with a Markes BenchTOF Select mass spectrometer featuring tandem ionization (Markes International). The GC transfer line was set at 270 °C. The TOF MS was tuned for single ionization at 70 eV, and the scan range was set between 35 and 350 *m*/*z* with a spectrum acquisition frequency of 100 Hz. The thermal modulator was a loop-type, two-stage KT 2004 (Zoex Corporation) cooled with liquid nitrogen and controlled by Optimode, v2.0 (SRA Intruments, Cernusco sul Naviglio). The modulation period (*P*_M_) was set at 3.5 s, while the hot-jet pulse duration was set at 250 ms. The cold-jet stream at the mass flow controller (MFC) was programmed to linearly reduce the total flow (i.e., 20 L/min) from 40% to 8% along the analytical run.

The column set consisted of a ^1^D HeavyWax column (100% poly(ethylene glycol) (PEG); 30 m × 0.25 mm *d*_c_ × 0.25 μm *d*_f_) coupled with a ^2^D DB17 column (50% phenyl-methylpolysiloxane; 1.0 m × 0.10 mm *d*_c_ × 0.10 μm *d*_f_), both supplied by Agilent Technologies. A fused silica capillary loop (1.0 m × 0.1 mm *d*_c_) was used in the modulator slit. SilTite μ-unions (Trajan Scientific and Medical) were used to connect the columns with the capillaries.

The GC split/splitless injector port was set at 250 °C and operated in pulsed-split mode (250 kPa overpressure applied to the injection port until 2 min) with a 1:20 split ratio. A special design liner for SPME thermal-desorption (Merck) was used to improve the transfer of the analytes to the ^1^D column and to limit band broadening in-space. Helium was used as the carrier gas at a nominal flow of 1.3 mL/min. The oven temperature program was set as follows: from 40 °C (2 min) to 240 °C (10 min) at 3.5 °C min^–1^.

The *n-*alkanes solution for I^T^s determination was analyzed under the following conditions: split/splitless injector in split mode, 1:50 split ratio, 250 °C injector temperature, and 1 μL injection volume.

#### HS-SPME-GC × GC-TOF MS Method Performance Parameters

The performance parameters of the method were evaluated to assess the repeatability for the retention times (^1^*t*_R_ and ^2^*t*_R_ over one-week) and for the 2D peak response indicators (i.e., absolute responses −2D peak *volumes* and % normalized responses over ISs–2D peak *percent response*). The % relative standard deviation (%RSD) was therefore calculated for retention indicators on all the targeted and untargeted components (UT features *n* = 452) for all the analyses run over a one week time frame (*n* = 35). The obtained results are reported in Supporting Information Table 1, together with the average retention times in the two chromatographic dimensions, the calculated retention indices (*I*^T^), and the tabulated values, according to the NIST database (NIST Standard Reference Database, 2005).^20^ The mean %RSD of the retention times was 0.79% for the ^1^D (^1^*t*_R_) and 4.09% for the ^2^D (^2^*t*_R_), respectively. The VOCs response indicators were evaluated on the quality control (QC) samples obtained by mixing ground silage from HIGH and LOW control (CON) samples and then randomly analyzing the obtained mixed samples over one week (*n* = 6). The obtained results are shown in Supporting Information Table 1 for the targeted peaks (*n* = 269) and untargeted features (*n* = 183). The “-” symbol in the table refers to those features that were not matched in the QC samples. The maximum %RSD (%RSD QC) of the absolute response was 36%, as reported for heptanoic acid, and the mean was 12.5 %.

#### Untargeted Targeted Fingerprinting by Pattern Recognition

Applying a data elaboration workflow to GC × GC-TOF MS data enables the peak and peak-region features to be captured from the untargeted and targeted components separated on the 2D retention-time plane. Such an approach is named UT fingerprinting.^21,22^ In this application, analyte targeting (i.e., identification) was performed as the last step, before data mining. A schematic workflow of the UT fingerprinting strategy is reported in reference literature by Cordero and Reichenbach.^22−24^

The strategy used to generate and realign untargeted features (i.e., peaks and peak-regions) across all the chromatograms is known as *template matching.*(25) This process is performed as a first step of the processing workflow. Metadata (retention times, MS fragmentation patterns, and detector responses) are extracted for 2D peaks and peak-regions; those that exceed a signal-to-noise (S/N) threshold value of 100 are used to establish correspondences across multiple chromatograms (*n* = 35) and to coherently realign them. Constraints are applied to validate positive matches between chemical entities in order to achieve adequate specificity.^11,26^ A spectral-similarity threshold of 750 was defined for the direct match factor (DMF) and reverse match factor (RMF) between the template (*reference*) and candidate (*analyzed*) MS signatures according to the NIST MS Search algorithm, ver. 2.0 (National Institute of Standards and Technology).^26^ The “peak spectra”, that is, the average MS signature from the largest data point within the 2D peak, was used for spectral matching.

The results of the fingerprinting are tables in which the 2D peaks and/or peak-regions are aligned across all the chromatograms with their-related metadata (e.g., ^1^D and ^2^D retention times – ^1^*t*_R_ and ^2^*t*_R_, MS fragmentation pattern, base peak and molecular ion *m*/*z*, and TIC response).

In this study, the process aligned the 35 acquired chromatograms using *reliable* peaks for registration and generated a *composite* chromatogram from which the peak-region features were delineated and extracted into a template that was used for further chromatogram processing. By applying constraints, the reliable peaks were those 2D peaks that positively matched all but one of the chromatograms (i.e., the most-constrained condition option).

**Figure 1:**
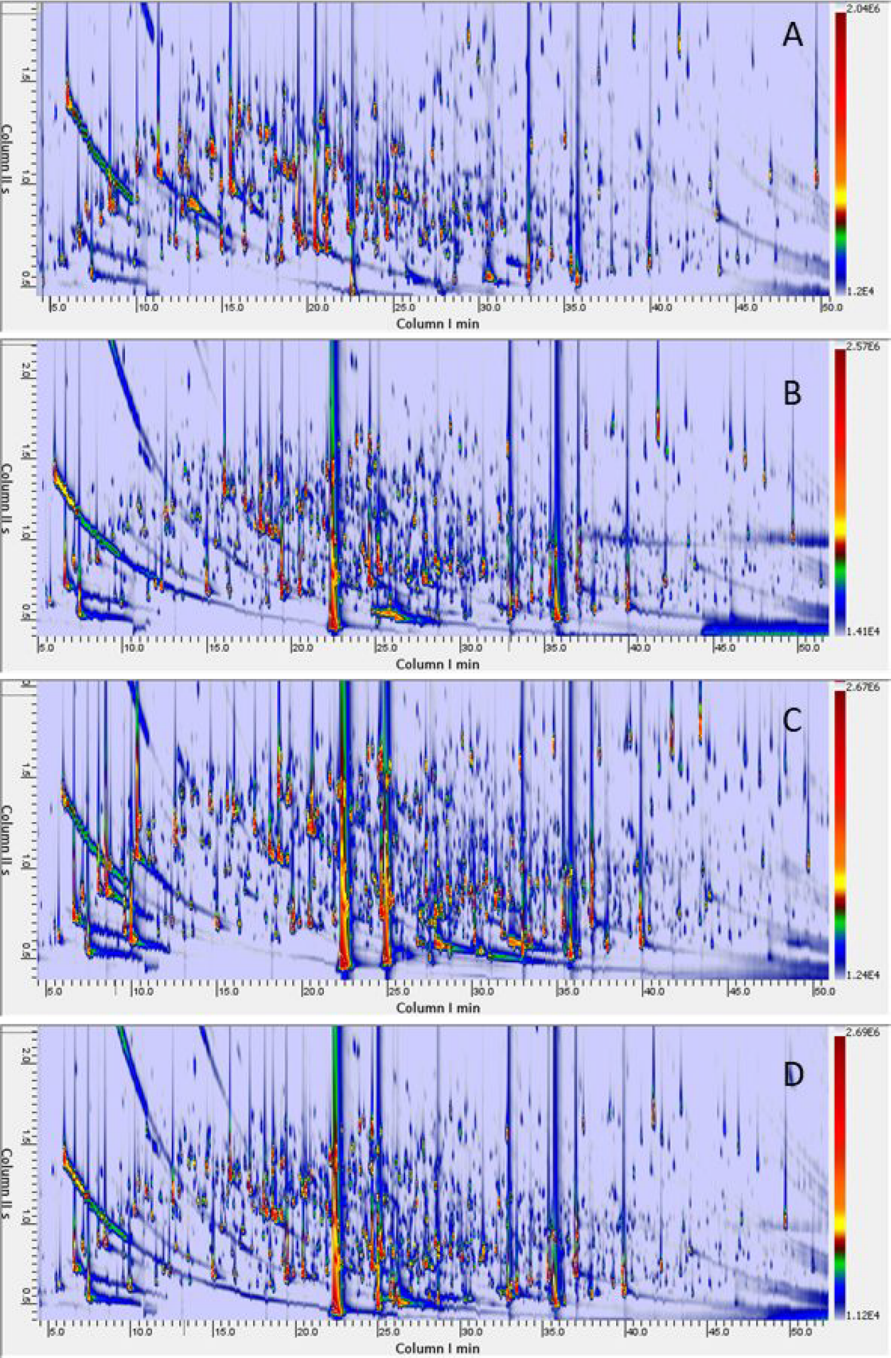
Contour plots showing the complex detectable volatilome of LOW DM herbage (**1A**) and corresponding fermented silages: controls CON (**1B**), *Lpar* (**1C**), and *Lbuc* (**1D**).

The last step of the process was that of targeting the informative compounds, and 269 putatively identified analytes were included.^27^Table 2 lists the target analytes, identified on the 70 eV spectra, according to spectral similarity criteria (DMF above 900 and RMF above 950) and an *I*^T^ tolerance of ±10 units. The analytes are listed according to their chemical classes together with their CAS registry number, ^1^*t*_R_, ^2^*t*_R_, and experimental I^T^s. Figure 1A–D shows the contour plots of the herbage and fermented LOW DM samples (i.e., Figure 1A *herbage*; Figure 1B control CON; Figure 1C *Lpar*; Figure 1D *Lbuc*).

**Table 2:** Target Components Mapped through All Analyzed Samples^a^

feature ID	CAS	^1^*t*_R_, min	%RSD	^2^*t*_R_, s	%RSD	*I*^T^ exp	*I*^T^ tab	*F* all
**Alcohols**								
Ethanol	64–17–5	7.21	0.13	2.33	1.24	948	944	ND
2-Butanol	78–92–2	9.30	1.22	0.64	5.89	1041	1036	ND
1-Propanol	71–23–8	9.66	1.16	0.59	6.48	1052	1051	ND
2-Methyl-1-propanol (isobutanol)	78–83–1	11.21	1.27	0.63	5.77	1099	1101	ND
3-Pentanol	584–02–1	11.64	1.29	0.71	5.63	1113	1111	ND
3-Methyl-2-butanol	598–75–4	12.00	1.29	0.71	5.78	1124	1118	21
1-Butanol	71–36–3	12.68	1.18	0.64	5.51	1146	1146	34
2-Methyl-3-pentanol	565–67–3	13.13	1.25	0.79	5.18	1161	1167	ND
1-Penten-3-ol	616–25–1	13.23	1.28	0.64	6.27	1164	1164	ND
3-Methyl-1-butanol (isoamyl alcohol)	123–51–3	14.64	1.21	0.69	6.19	1209	1211	ND
2-Hexanol	626–93–7	15.04	1.17	0.76	5.70	1222	1222	ND
1-Pentanol	71–41–0	15.95	1.12	0.69	5.40	1251	1252	ND
4-Heptanol	589–55–9	16.99	1.07	0.85	4.27	1284	1285	ND
(E)-2-Penten-1-ol	1576–96–1	17.88	1.02	0.63	5.98	1313	1310	ND
2-Heptanol	543–49–7	18.08	1.08	0.82	4.50	1320	1319	39
(Z)-2-Penten-1-ol	1576–95–0	18.10	1.04	0.63	5.96	1320	1317	12
1-Hexanol	111–27–3	19.07	1.05	0.73	5.13	1352	1344	18
(E)-3-Hexen-1-ol	928–97–2	20.07	0.94	0.69	5.36	1385	1373	5
3-Octanol	589–98–0	20.31	0.88	0.91	4.27	1393	1398	ND
(Z)-2-Hexen-1-ol	928–94–9	20.66	1.43	0.69	7.27	1405	1401	25
4-Hexen-1-ol	6126–50–7	20.76	0.76	0.67	5.51	1408	1408	73
(E)-2-Hexen-1-ol	928–95–0	20.86	1.31	0.67	5.19	1412	1411	ND
2-Octanol	123–96–6	21.07	0.86	0.87	4.23	1419	1405	ND
1-Octen-3-ol	3391–86–4	21.99	0.85	0.78	4.76	1451	1450	79
6-Methyl-5-hepten-2-ol	1569–60–4	22.31	0.82	0.79	4.52	1462	1465	8
4-Nonanol	5932–79–6	22.84	0.53	0.97	2.03	1481	1479	10
2-Ethylhexanol	104–76–7	23.17	0.84	0.82	5.84	1492	1484	ND
(Z)-4-Hepten-1-ol	20851–55–2	23.46	0.77	0.72	5.21	1502	1502	ND
(E)-2-Hepten-1-ol	33467–76–4	23.61	1.13	0.75	9.91	1507	1504	ND
1-Octanol	111–87–5	24.97	0.77	0.81	5.07	1557	1555	ND
2,3-Butanediol	513–85–9	25.38	0.72	0.55	6.89	1572	1583	ND
2,4-Hexadien-1-ol	111–28–4	25.89	0.20	0.61	3.79	1591	1588	6
(5E)-3,7-Dimethyl-1,5,7-octatrien-3-ol (hotrienol)	53834–70–1	26.46	0.70	0.81	5.54	1611	1602	ND
(E)-2-Octen-1-ol	18409–17–1	26.52	0.68	0.75	4.96	1613	1611	ND
2-(2-Ethoxyethoxy)ethanol	111–90–0	26.86	0.61	0.68	3.65	1623	1615	ND
1-Nonanol	143–08–8	27.63	0.67	0.85	4.90	1648	1663	ND
6-Undecanol	23708–56–7	27.80	0.12	0.81	4.28	1654	1640	ND
(Z)-3-Nonen-1-ol	10340–23–5	28.51	0.18	0.81	2.86	1677	1682	ND
1-Decanol	112–30–1	30.24	0.64	0.90	4.33	1741	1748	ND
2-(2-Butoxyethoxy)ethanol	112–34–5	31.18	0.55	0.77	5.05	1780	1786	ND
1-Tetradecanol	112–72–1	39.78	0.44	1.06	3.68	2159	2157	ND
**Aldehydes**								
2-Methylpropanal	78–84–2	5.44	0.48	0.66	5.15	821	819	ND
Acrolein	107–02–8	5.86	0.51	0.58	6.95	834	840	ND
3-Methylbutanal	590–86–3	6.99	0.81	0.82	5.30	929	936	42
2-Butenal	4170–30–3	9.94	1.31	0.75	7.01	1060	1061	210
Hexanal	66–25–1	11.02	1.20	1.04	3.97	1093	1098	22
(E)-2-Pentenal	1576–87–0	12.40	1.19	0.87	4.31	1137	1147	63
(E)-3-Hexenal	69112–21–6	12.83	0.00	0.89	4.62	1151	1146	ND
(Z)-3-Hexenal	6789–80–6	13.13	0.80	0.88	5.38	1161	1158	16
2-Methyl-2-pentenal	623–36–9	13.46	1.24	0.97	5.30	1171	1171	ND
Heptanal	111–71–7	14.12	1.27	1.17	3.60	1193	1190	ND
3-Methyl-2-butenal	107–86–8	14.63	1.16	0.86	6.30	1209	1212	15
(Z)-2-Hexenal	16635–54–4	14.78	0.20	0.96	3.85	1214	1214	5
(E)-2-Hexenal	6728–26–3	15.15	1.10	0.98	4.21	1226	1220	ND
Octanal	124–13–0	17.35	1.09	1.25	3.10	1296	1291	ND
(E)-2-Heptenal	18829–55–5	18.42	1.02	1.05	3.69	1331	1318	ND
Nonanal	124–19–6	20.51	0.92	1.29	3.12	1400	1392	9
2,4-Hexadienal	80466–34–8	20.76	0.84	0.82	4.98	1409	1402	ND
(Z)-2-Octenal	20664–46–4	20.91	0.26	1.10	2.89	1414	1413	9
2-Furancarboxaldehyde (furfural)	98–01–1	21.50	0.85	0.63	5.71	1434	1437	ND
(E)-2-Octenal	2548–87–0	21.56	0.81	1.10	3.50	1436	1434	20
(E,Z)-2,4-heptadienal	4313–02–4	22.52	0.84	0.89	4.28	1469	1464	14
2,4-Heptadienal	5910–85–0	23.34	0.77	0.88	4.42	1498	1489	ND
Decanal	112–31–2	23.54	0.76	1.34	3.10	1505	1505	ND
(E)-2-Nonenal	18829–56–6	24.58	0.85	1.17	2.86	1543	1530	153
(E,Z)-2,6-Nonadienal	557–48–2	26.06	0.58	1.01	3.23	1597	1590	ND
β-Cyclocitral	432–25–7	26.93	0.69	1.14	3.44	1626	1611	ND
(E)-2-Decenal	3913–81–3	27.45	0.68	1.20	3.40	1643	1625	ND
(2Z)-3,7-Dimethyl-2,6-octadienal (neral)	106–26–3	28.69	0.60	1.07	3.35	1683	1663	ND
2,4-Nonadienal	6750–03–4	29.09	0.21	0.98	3.77	1695	1668	ND
Dodecanal	112–54–9	29.17	0.63	1.42	3.16	1698	1708	ND
(E)-2-Undecenal	53448–07–0	30.33	0.47	1.24	3.54	1745	1755	ND
(E,Z)-2,4-decadienal	25152–83–4	31.35	1.37	1.03	3.97	1786	1778	ND
Tridecanal	10486–19–8	31.78	0.64	1.46	3.13	1804	1821	27
Tetradecanal	124–25–4	34.25	0.52	1.49	2.94	1908	1920	ND
trans-4,5-Epoxy-(E)-2-decenal	134454–31–2	35.95	0.88	0.89	6.36	1983	1995	ND
Pentadecanal	2765–11–9	36.78	0.15	1.54	1.54	2020	2040	ND
**Aromatics**								
Toluene	108–88–3	9.81	1.14	0.90	4.86	1056	1054	ND
Ethylbenzene	100–41–4	12.39	0.53	1.05	2.69	1137	1136	7
p-Xylene	106–42–3	12.52	1.15	1.06	4.16	1141	1142	ND
m-Xylene	108–38–3	12.69	1.23	1.04	3.65	1147	1143	ND
o-Xylene	95–47–6	14.05	1.09	1.03	3.87	1190	1188	15
Propylbenzene	103–65–1	14.92	0.87	1.16	3.80	1218	1213	59
1-Ethyl-2-methylbenzene	611–14–3	15.43	1.00	1.16	4.08	1235	1235	152
1,2,4-Trimethylbenzene	95–63–6	17.07	1.00	1.12	3.44	1287	1287	ND
Benzaldehyde	100–52–7	24.24	0.75	0.75	4.87	1531	1529	ND
Methyl benzoate	93–58–3	26.98	0.64	0.81	5.00	1627	1631	ND
Phenylacetaldehyde	122–78–1	27.43	0.67	0.76	4.83	1642	1625	12
Acetophenone	98–86–2	27.65	0.82	0.79	3.98	1649	1634	11
Ethyl benzoate	93–89–0	28.12	0.65	0.88	4.41	1664	1673	ND
1,3-Dimethoxybenzene	151–10–0	30.08	0.63	0.78	4.97	1735	1730	ND
Naphthalene	91–20–3	30.09	0.17	0.85	2.73	1735	1743	ND
Ethyl phenylacetate	101–97–3	30.99	0.59	0.86	5.04	1772	1775	9
2-Methoxyphenol (guaiacol)	90–05–1	32.68	0.63	0.63	6.80	1842	1860	ND
Propyl phenylacetate	4606–15–9	32.91	0.25	0.90	3.82	1852	1848	17
Benzyl alcohol	100–51–6	33.00	0.56	0.60	6.62	1855	1864	ND
Ethyl 3-phenylpropanoate	2021–28–5	33.40	0.59	0.91	4.68	1872	1892	ND
2-Phenylethanol	60–12–8	33.84	0.54	0.65	5.64	1890	1890	ND
2-Methoxy-4-methylphenol	93–51–6	34.88	0.54	0.68	6.03	1936	1938	ND
Phenol	108–95–2	35.88	0.51	0.54	7.72	1980	1994	ND
p-Cresol	106–44–5	37.49	0.45	0.57	6.71	2053	2057	ND
2-Methoxy-4-propylphenol (4-propylguaiacol)	2785–87–7	38.23	0.46	0.75	5.58	2087	2084	ND
(Z)-3-Hexenyl benzoate	25152–85–6	38.75	0.45	1.00	4.24	2112	2120	ND
Phenoxyethanol	122–99–6	38.85	0.44	0.64	6.54	2116	2115	27
2,3-Dimethylphenol	526–75–0	39.50	0.45	0.60	6.01	2146	2155	ND
4-Ethenyl-2-methoxyphenol (4-vinylguaiacol)	7786–61–0	39.96	0.43	0.68	5.95	2168	2175	ND
Ethyl 2-hydroxy-3-phenylpropanoate	15399–05–0	41.75	0.40	0.75	5.43	2259	2249	ND
2-Methoxy-4-(1-propen-1-yl)phenol (isoeugenol)	97–54–1	42.90	0.37	0.71	6.73	2317	2316	211
**Acids**								
Acetic acid	64–19–7	22.04	1.22	0.47	8.15	1453	1452	ND
Propionic acid	79–09–4	24.73	1.04	0.49	7.54	1548	1544	27
Isobutyric acid	79–31–2	25.66	0.60	0.53	9.96	1583	1580	8
Butyric acid	107–92–6	27.39	0.36	0.49	7.32	1641	1624	ND
Isovaleric acid	503–74–2	28.38	0.64	0.52	7.46	1672	1653	ND
Pentanoic acid	109–52–4	30.07	0.64	0.53	7.37	1734	1733	18
Hexanoic acid	142–62–1	32.66	0.53	0.54	7.29	1841	1840	5
Heptanoic acid	111–14–8	35.21	0.47	0.56	7.03	1950	1960	ND
Octanoic acid	124–07–2	37.76	0.07	0.60	3.85	2066	2068	ND
Nonanoic acid	112–05–0	39.86	0.40	0.61	6.30	2163	2173	6
Decanoic acid	334–48–5	41.93	0.39	0.64	6.35	2268	2270	ND
Dodecanoic acid	143–07–7	45.92	0.57	0.70	6.46	2478	2469	ND
**Esters**								
Methyl acetate	79–20–9	5.64	0.68	0.60	6.24	827	832	ND
Ethyl acetate	141–78–6	6.48	0.84	0.71	5.63	854	870	20
Ethyl propionate	105–37–3	7.87	1.07	0.87	4.24	993	964	ND
Propyl acetate	109–60–4	8.23	1.23	0.88	5.22	1008	996	ND
Methyl butyrate	623–42–7	8.59	0.31	0.86	4.31	1019	1004	ND
Methyl isovalerate	556–24–1	9.45	0.00	0.98	2.82	1045	1025	ND
Propyl propionate	106–36–5	10.01	1.25	1.08	3.64	1062	1056	ND
Ethyl 2-methylbutanoate	7452–79–1	10.22	1.24	1.22	3.33	1069	1063	ND
Ethyl isovalerate	108–64–5	10.67	1.21	1.18	3.34	1083	1079	13
2-Pentyl acetate	626–38–0	10.80	1.23	1.15	3.71	1087	1080	48
Methyl pentanoate	624–24–8	11.14	1.05	1.02	2.80	1097	1090	8
Isoamyl acetate	123–92–2	12.25	1.26	1.14	3.79	1133	1126	ND
Propyl butyrate	105–66–8	12.32	0.66	1.22	3.65	1135	1133	ND
Ethyl pentanoate	539–82–2	12.63	1.22	1.19	3.55	1145	1142	ND
Propyl isovalerate	557–00–6	13.22	1.00	1.37	2.75	1164	1153	535
Amyl acetate	628–63–7	13.80	1.26	1.15	4.10	1182	1177	6
Methyl hexanoate	106–70–7	14.23	1.21	1.13	3.96	1196	1190	94
Isoamyl propionate	105–68–0	14.29	1.17	1.34	3.60	1198	1192	6
Propyl pentanoate	141–06–0	15.24	1.13	1.36	3.64	1228	1217	ND
Butyl butyrate	109–21–7	15.34	0.00	1.35	0.00	1232	1230	ND
Ethyl hexanoate	123–66–0	15.67	1.09	1.30	3.13	1242	1240	ND
Methyl (Z)-3-hexenoate	13894–62–7	16.49	1.08	0.98	4.11	1268	1265	15
2-Heptyl acetate	5921–82–4	16.55	1.06	1.39	3.53	1270	1255	ND
Isoamyl butyrate	106–27–4	16.69	0.84	1.48	2.56	1275	1270	ND
Hexyl acetate	142–92–7	16.88	1.05	1.22	2.98	1281	1276	ND
Methyl (E)-2-hexenoate	13894–63–8	17.60	0.17	1.05	0.98	1304	1305	35
(E)-3-Hexenyl acetate	3681–82–1	18.02	0.95	1.07	3.94	1318	1306	14
(Z)-3-Hexenyl acetate	3681–71–8	18.25	1.08	1.06	3.68	1325	1319	ND
Propyl hexanoate	626–77–7	18.28	0.97	1.42	2.96	1326	1316	17
Ethyl heptanoate	106–30–9	18.70	0.96	1.36	3.17	1340	1332	ND
Propanoic acid, 2-hydroxy-, ethyl ester (ethyl lactate)	687–47–8	18.96	0.98	0.67	5.65	1349	1356	ND
Ethyl 2-hexenoate	1552–67–6	19.10	1.01	1.14	3.59	1353	1343	17
Isobutyl hexanoate	105–79–3	19.30	0.93	1.58	2.98	1360	1351	ND
Heptyl acetate	112–06–1	19.92	0.98	1.28	3.71	1381	1370	ND
Ethyl (4E)-4-heptenoate	54340–70–4	20.22	1.13	1.19	3.83	1390	1382	20
Butyl-(Z)-3-hexenoate	69668–84–4	21.14	0.90	1.32	3.47	1422	1421	ND
Ethyl 2-hydroxy-3-methyl butyrate	2441–06–7	21.51	0.94	0.80	1.13	1434	1422	ND
Ethyl octanoate	106–32–1	21.72	0.84	1.42	3.08	1442	1440	ND
Isoamyl hexanoate	2198–61–0	22.43	0.79	1.59	2.78	1466	1453	ND
(E)-3-Hexenyl butyrate	53398–84–8	22.53	0.97	1.30	5.05	1470	1466	12
Octyl acetate	112–14–1	22.90	0.75	1.33	3.07	1483	1480	ND
Ethyl 4-octenoate	138234–61–4	22.99	0.77	1.24	4.03	1485	1470	ND
Ethyl (E,E)-2,4-Hexadienoate (ethyl sorbate)	2396–84–1	23.76	0.75	0.95	4.51	1513	1501	ND
Ethyl nonanoate	123–29–5	24.56	0.74	1.46	2.99	1542	1530	ND
Isopentyl 2-hydroxypropanoate (isoamyl lactate)	19329–89–6	25.47	0.72	0.81	4.98	1576	1583	ND
Nonyl acetate	143–13–5	25.70	0.68	1.38	3.12	1584	1582	11
1,2-Propanediol, 2-acetate	6214–01–3	26.55	0.73	0.60	7.24	1613	1621	ND
Hexyl hexanoate	6378–65–0	26.61	0.69	1.59	2.74	1615	1599	ND
α-Methyl-γ-butyrolactone	1679–47–6	26.85	0.10	0.77	4.17	1623	1625	20
γ-Butyrolactone	96–48–0	27.09	0.71	0.71	4.87	1631	1635	ND
Ethyl decanoate	110–38–3	27.29	0.64	1.49	4.33	1637	1624	ND
(Z)-3-Hexenyl hexanoate	31501–11–8	27.80	0.67	1.39	3.19	1654	1638	ND
Butanedioic acid, 1,4-diethyl ester (diethyl succinate)	123–25–1	28.28	0.67	0.87	5.02	1669	1677	ND
γ-Hexalactone	695–06–7	28.99	0.65	0.82	4.49	1692	1689	ND
Propyl decanoate	30673–60–0	29.44	0.28	1.59	2.73	1709	1722	ND
Propanoic acid, 2-hydroxy-, (3Z)-3-hexenyl ester ((E)-3-hexenyl lactate)	61931–81–5	29.45	0.63	1.22	3.76	1709	1727	ND
3-Methyl-2(5H)-furanone	22122–36–7	29.46	0.48	0.71	5.88	1709	1713	ND
Benzyl acetate	140–11–4	29.65	0.60	0.79	4.83	1717	1733	ND
Methyl 2-hydroxy-benzoate (methyl salicylate)	119–36–8	30.91	0.58	0.81	5.01	1768	1757	ND
2-Phenylethyl acetate	103–45–7	31.79	0.59	0.85	4.66	1804	1820	6
Ethyl dodecanoate	106–33–2	32.44	0.57	1.57	3.15	1832	1848	ND
Propyl dodecanoate	3681–78–5	34.33	0.36	1.64	2.39	1911	1927	ND
γ-Nonalactone	104–61–0	36.64	0.50	0.96	4.30	2014	2020	ND
Isopropyl tetradecanoate (isopropyl myristate)	110–27–0	36.87	0.47	1.77	2.49	2025	2026	10
Ethyl tetradecanoate (ethyl myristate)	124–06–1	37.08	0.47	1.63	2.96	2034	2046	ND
Methyl hexadecanoate (methyl palmitate)	112–39–0	40.61	0.42	1.56	3.01	2204	2210	ND
Ethyl hexadecanoate (Ethyl palmitate)	628–97–7	41.36	0.39	1.69	3.15	2240	2254	ND
Ethyl (E)-9-hexadecenoate	54546–22–4	41.94	0.45	1.56	3.54	2269	2277	ND
Propyl hexadecanoate (propyl palmitate)	2239–78–3	43.04	0.37	1.76	2.81	2324	2335	ND
Ethyl (Z)-9-octadecenoate (Ethyl oleate)	111–62–6	45.69	0.35	1.62	3.05	2466	2470	ND
Ethyl (Z,Z)-9,12-Octadecadienoate (ethyl linoleate)	544–35–4	46.52	0.33	1.51	3.81	>2500		ND
Ethyl (Z,Z,Z)-9,12,15-Octadecatrienoate (ethyl linolenate)	1191–41–9	47.70	0.31	1.39	3.76	>2500		ND
**Heterocyclic compounds**								
2-Methylfuran	534–22–5	6.24	0.00	0.66	0.00	846	850	ND
2-Ethylfuran	3208–16–0	7.78	0.90	0.79	5.75	987	965	ND
2-Pentylfuran	3777–69–3	15.56	1.12	1.19	3.63	1239	1235	32
2-Furanmethanol (furfuryl alcohol)	98–00–0	27.68	0.76	0.55	6.78	1650	1651	ND
2-Ethyl-3-methyl maleimide	20189–42–8	41.56	0.41	0.62	5.80	2250	2260	ND
**Hydrocarbons**								
Propane	74–98–6	4.49	2.45	0.50	6.96	NC	300	ND
Heptane	142–82–5	4.49	0.00	0.86	4.36	700	700	ND
Octane	111–65–9	5.25	0.22	1.30	3.63	800	800	5
Nonane	111–84–2	6.59	0.75	1.90	3.14	900	900	10
(E)-1,3-Octadiene	1002–33–1	7.80	0.76	1.27	4.28	989	958	234
Undecane	1120–21–4	11.24	1.63	2.98	2.69	1100	1100	ND
1-Undecene	821–95–4	12.67	1.03	2.36	1.81	1146	1142	ND
Dodecane	112–40–3	14.38	1.12	3.06	2.09	1200	1200	ND
Tridecane	629–50–5	17.48	1.00	3.05	2.17	1300	1300	ND
1-Tetradecene	1120–36–1	21.84	1.21	2.46	2.49	1446	1428	23
Tetradecane	629–59–4	20.51	0.86	2.99	1.46	1400	1400	ND
Pentadecane	629–62–9	23.41	0.82	2.97	2.47	1500.00	1500	ND
Hexadecane	544–76–3	26.13	0.64	2.88	1.98	1600	1600	ND
**Ketones**								
Acetone	67–64–1	5.48	0.00	0.58	6.38	822	821	ND
Methyl ethyl ketone	78–93–3	6.71	0.00	0.72	4.94	909	905	ND
2-Pentanone	107–87–9	8.28	0.96	0.87	2.96	1010	1007	ND
Butanedione	431–03–8	8.37	1.24	0.64	6.45	1013	993	ND
2-Methyl-3-pentanone	565–69–5	8.72	1.26	1.04	4.09	1023	1003	ND
1-Penten-3-one	1629–58–9	9.33	1.22	0.80	5.36	1042	1024	ND
2,3-Pentanedione	600–14–6	10.56	0.00	0.78	0.00	1079	1070	ND
2-Heptanone	110–43–0	14.00	1.13	1.13	3.17	1189	1184	24
6-Methyl-2-heptanone	928–68–7	15.87	0.00	1.23	0.00	1248	1236	ND
5-Methyl-3-heptanone	541–85–5	16.26	1.04	1.30	3.05	1261	1265	6
2-Octanone	111–13–7	17.26	1.15	1.23	2.62	1293	1291	5
3-Hydroxy-2-butanone (acetoin)	513–86–0	17.32	1.14	0.64	5.74	1295	1287	8
2,2,6-Trimethylcyclohexanone	2408–37–9	18.27	1.03	1.31	3.03	1326	1320	81
4-Nonanone	4485–09–0	18.47	1.13	1.41	3.59	1333	1322	7
(Z)-6-Octen-2-one	74810–53–0	18.73	0.48	1.06	3.24	1341	1316	20
6-Methyl-5-hepten-2-one	110–93–0	18.84	0.94	1.04	3.83	1345	1340	32
2-Nonanone	821–55–6	20.49	0.52	1.28	1.91	1399	1386	ND
(E,Z)-3,5-Octadien-2-one	30086–02–3	24.21	0.18	0.93	2.79	1529	1513	39
(E,E)-3,5-Octadien-2-one	30086–02–3	25.61	0.17	0.92	1.92	1581	1570	ND
2-Undecanone	112–12–9	26.41	0.20	1.37	1.69	1609	1606	33
6,10-Dimethyl-2-undecanone	1604–34–8	28.59	0.64	1.50	3.18	1679	1660	ND
6,10,14-Trimethyl-2-pentadecanone	502–69–2	38.70	0.44	1.71	2.99	2110	2110	ND
**Others**								
Styrene	100–42–5	16.30	1.09	0.88	4.34	1262	1264	ND
Hexanenitrile	628–73–9	17.69	1.02	0.93	4.27	1307	1303	51
1-Nitropentane	628–05–7	20.78	0.97	0.88	4.05	1409	1409	ND
1-Nitrohexane	646–14–0	23.86	0.18	0.94	3.38	1517	1511	ND
2,3,3a,4,5,7a-Hexahydro-3,6-dimethylbenzofuran	70786–44–6	23.95	0.75	1.22	3.33	1520	1527	15
Dimethyl Sulfoxide	67–68–5	24.97	0.00	0.70	1.42	1557	1560	ND
3,5,5-Trimethyl-2-cyclohexene-1,4-dione (ketoisophorone)	1125–21–9	28.95	0.09	0.89	2.60	1691	1676	23
3,4-Dimethyl-2,5-furandione	766–39–2	30.02	0.33	0.80	5.50	1732	1714	ND
Bis(2-hydroxypropyl) ether	110–98–5	31.90	0.74	0.56	3.77	1809	1817	ND
5,6,7,7a-Tetrahydro-4,4,7a-trimethyl-2(4*H*)-benzofuranone	15356–74–8	43.16	0.38	1.02	4.15	2331	2325	ND
Hexadecanolide	109–29–5	43.85	0.44	1.58	3.16	2368	2367	ND
**Terpenes**								
Limonene	138–86–3	14.48	1.12	1.51	3.04	1204	1194	ND
β-Ocimene	13877–91–3	16.08	1.06	1.37	4.43	1255	1254	ND
p-Cymene	535–77–3	16.78	0.91	1.25	1.54	1277	1270	21
cis-Linalool oxide (furanoid)	60047–17–8	21.97	0.88	1.02	3.80	1450	1441	22
Nerol oxide	1786–08–9	22.65	0.82	1.14	3.67	1474	1469	ND
trans-Linalool oxide (furanoid)	34995–77–2	22.74	0.83	1.00	4.48	1477	1469	ND
Cyclosativene	22469–52–9	23.22	0.84	2.09	2.45	1494	1487	ND
Copaene	3856–25–5	23.45	0.82	2.07	2.03	1501	1489	ND
(E)-Theaspirane	43126–22–3	23.72	0.74	1.64	2.65	1511	1500	ND
Linalool	78–70–6	24.73	0.76	0.87	4.09	1549	1544	ND
Theaspirane	36431–72–8	24.75	0.77	1.57	3.18	1549	1540	ND
β-Caryophyllene	87–44–5	26.39	0.74	1.84	2.60	1608	1598	ND
Menthol	2216–51–5	27.26	0.67	0.93	4.22	1636	1626	10
α-Terpineol	98–55–5	28.66	0.64	0.88	4.58	1681	1687	ND
α-Humulene	6753–98–6	29.02	0.61	1.68	2.95	1693	1678	ND
β-Bisabolene	495–61–4	29.65	0.64	1.55	3.36	1717	1723	65
Geranial	141–27–5	29.81	0.50	1.07	4.14	1724	1729	ND
Curcumene	644–30–4	30.79	0.61	1.35	3.33	1764	1766	ND
β-Damascenone	23726–93–4	31.90	0.59	1.14	3.80	1809	1821	9
Dihydro-β-ionone	17283–81–7	32.21	0.55	1.28	3.29	1822	1825	ND
Geraniol	106–24–1	32.30	0.58	0.79	4.60	1826	1836	ND
Geranylacetone	3796–70–1	32.67	0.56	1.20	3.59	1841	1852	ND
Neophytadiene	504–96–1	34.25	0.52	2.26	2.44	1908	1915	ND
β-Ionone	79–77–6	34.64	0.53	1.21	3.40	1925	1926	ND
5,6-Epoxy-β-ionone	23267–57–4	35.93	0.34	1.15	3.62	1982	1977	7

a Target analytes, reported with corresponding CAS registry number, were identified according to criteria of spectral similarity (DMF above 900 and RMF above 950) and *I*^T^ tolerance of ±15 units. Analytes are listed with retention times (^1^*t*_R_, ^2^*t*_R_) and corresponding precision data expressed as %RSD across all analyses (*n* = 35), experimental linear retention index (*I*^T^), and tabulated *I*^T^ (NIST database https://webbook.nist.gov/chemistry/), Fisher ratio (*F*) values calculated for all classes (F all). When features were invariant (e.g., undetected) within a class, the Fisher ratio cannot be computed and in the table is reported as “ND”.

#### Composite Class Image Fingerprinting

The composite class image fingerprinting, adopted to promptly highlight any pattern differences between the sample classes, is an automated procedure that was designed in a previous study to detect hazelnut spoilage volatiles.^28^ In this procedure, 2D chromatograms, preprocessed and elaborated by means of UT fingerprinting (see section Untargeted Targeted Fingerprinting by Pattern Recognition), are grouped according to their sample class and combined in composite chromatograms from raw silage samples with the three fermented additional classes (i.e., *Lpar*, *Lbuc*, and CON) that are representative of each class.

Composite images were generated for all the sample classes (*n* = 4), and they included both biological (*n* = 2) and analytical (*n* = 2) replicates. The composite images resulting from this step of the process were then adopted for a comparative visualization (i.e., visual feature fingerprinting^14^) to track any differences in pattern between classes. The UT template built according to the procedure described in the *experimental section*, which included reliable untargeted and targeted peaks, was matched with composite class chromatograms and UT peak responses for further processing.

#### Data Acquisition, 2D Data Processing, and Statistical Analysis

Raw chromatographic data were acquired by TOF-DS software (Markes International) and processed by GC Image V2020 r1.2 suite (GC Image, LLC).

Heatmap visualization and Hierarchical Clustering (HC) were conducted using Gene-E (https://software.broadinstitute.org/GENE-E). Statistical analysis and chemometrics were performed using GC Investigator (GC Image), XLSTAT statistical and data analysis solution (Addinsoft 2020), and Microsoft Office Excel 2016 (Microsoft).

An unpaired *t*-test was used to compare the effect of DM level (LOW or HIGH) on the mean values of samples before ensiling. The fermentative characteristics, microbial counts, chemical characteristics, and aerobic stability of silages were analyzed by means of a two-way analysis of variance in a completely randomized design. The used statistical model was as follows: *Y*_ijk_ = μ + α_i_ + β_j_ + αβ_ij_ + ε_ijk_, where *Y*_ijk_ = observation, μ = overall mean, α_i_ = DM level fixed effect (i = LOW or HIGH), β_j_ = inoculum fixed effect (j = CONT or *Lbuc* or *Lpar*), αβ_ij_ = interaction effect, and ε_ijk_ = error. The analyses were performed using the R software (R ver. 4.0.3). When the calculated values of *F* were significant, the Bonferroni posthoc test (*P* < 0.05) was used to interpret any significant differences among the mean values.

## Results and Discussion

### Main Fermentative and Microbial Characteristics of the Herbage at Ensiling and Silages

The main chemical and microbial characteristics of the herbage prior to ensiling are reported in Table 1. The early seeded maize (HIGH) presented a higher DM content and a lower nitrate content than the late seeded maize (LOW). This is representative of the agronomic practices of Northern Italy and represents the range of DM content commonly found in corn silages. The microbial counts of the lactic acid bacteria, yeast, mold, and enterobacteria were also greater in the HIGH treatment than in the LOW one. Table 1 also reports the main fermentative products, microbiological counts, DM losses, and aerobic stability of the silages after 229 d of anaerobic conservation. The LOW silages presented a lower pH in the CON silages than in the *Lbuc* and *Lpar* treatments, with the highest value for *Lpar*. Nitrates were reduced from ensiling and resulted below the detection limit in the *Lpar* treatment. Lactic acid bacteria (LAB) were higher in *Lbuc* and *Lpar* than in the CON silages, whereas yeast, mold, and enterobacteria were close to or below the detection limit, with some differences between treatments. The DM losses were the highest for *Lpar* and the lowest for CON for both DM contents, whereas the aerobic stability was greatest for *Lpar* and lowest for CON and *Lbuc*.

The fermentation patterns of the silages presented different lactic and acetic acid contents between treatments, which in turn affected the lactic-to-acetic ratio. On one hand, 1,2-propanediol, a marker of heterolactic fermentation by *Lentilactobacillus* genus bacteria, was present in CON and *Lbuc*, whereas it was below the detection limit in the *Lpar* LOW DM silages. On the other hand, *Lpar* showed a larger amount of 1-propanol and propionic acid than CON and *Lbuc*, likely attributable to a secondary degradation of 1,2-propandiol into propionic acid and 1,2-propanol, as has also been witnessed for the metabolic activity of *Len. diolivorans* in silage.^29^ Ethanol was present in all the silages, with amounts ranging from 11.56 to 15.21 g kg^–1^ DM, without any meaningful variations between the LOW and HIGH DM silages.

### Compositional Complexity of the Volatilome and the Major Chemical Classes

The detectable volatilome of the maize silage was the result of multiple and concurrent chemical reactions catalyzed by endogenous and exogenous enzymes from native and inoculated microorganisms and of the environmental conditions that changed during fermentation, due to anaerobiosis, acidification (from a pH of around 6.0 at ensiling to 3.6 and 4.4), and to temperature increases in the first days of ensiling (from 20 to 28 °C). The comprehensive capturing of compositional changes (qualitative and quantitative) between samples enabled an accurate evaluation to be made on the differential impact of fermentation and also suggested the predominance of specific metabolic pathways.

The chemical complexity encrypted in the volatilome of the samples included several chemical classes that are closely correlated with known metabolic pathways triggered by the microbial fermentation of primary and specialized plant metabolites. Of the 269 putatively identified components, 72 of them were esters, 41 alcohols, 37 aldehydes, 22 ketones, and 12 were carboxylic acids. The congeners are listed according to their retention order in polar columns in Table 2. Additional classes of interest, because of their biological role, are aromatic derivatives (*n* = 31) and terpenes/terpenoids (*n* = 25). The strong signature of lignocellulosic material oxidation is evident within the aromatic derivatives, with several phenol and methoxyphenol derivatives. The presence of terpenes/terpenoids, specialized plant metabolites, comes from the plant at harvesting. They are here represented by native monoterpenoids (e.g., limonene, linalool, α-terpineol, menthol, geraniol, *p*-cymene, neral, geranial) and sesquiterpenoids (e.g., α-humulene, β-bisabolene, β-caryophyllene), some characteristic oxidation products (e.g., *cis-* and *trans*-linalool oxide, nerol oxide, theaspiranes), and nor-isoprenoids (e.g., β-damascenone, β-ionone, and dihydro-β-ionone). The latter class was likely formed by the oxidative degradation of carotenoids, which usually occurs as a result of enzymatic catalysis.

Terpenes are within the few groups of molecules that are not altered during the digestion of ruminants and which are partially carried over into milk and dairy products, thus giving such products a signature that impacts the sensorial response to milk and cheeses and connects the product to the diet fed to cows.^30^ In this study, some terpenes/terpenoids remained almost unchanged after the fermentation process, whereas some others, which had not been detected in the herbages prior to ensiling, appeared after fermentation [e.g., β-damascenone, (*E*)-theaspirane, α-terpineol, linalool, β-ionone, neral, and nerol-oxide] or even decreased (e.g., limonene, *p*-cimene, geranylacetone, β-bisabolene, neophytadiene, menthol, β-cyclosativene, β-caryophyllene), thus suggesting a microbial action on native substrates that in some cases was differentiated by the inocula (i.e., *Lpar* mostly degraded cis-linalool oxides).

Organic acids, from C2 to C12, were present in the herbage prior to ensiling; some homologues (C4 and C8) were depleted by fermentation, while others remained unvaried (C6, C7, C9, C12) or greatly increased in all the treatments (C2, C3, C5, isovaleric, and C10). Isobutyric acid was up-modulated by the *Lpar* inoculum.

The fermentation process produced a great variety of alcohols, including: ethanol, 1-propanol, 2-butanol, 3-methyl-2-butanol, 3-methyl-1-butanol (isoamyl alcohol), 3-pentanol, (*Z*)-2-hexen-1-ol, 2-octanol, 3-octanol, 1-octen-3-ol, 1-nonanol, 1-decanol, and 1-tetradecanol. A series of aromatic derivatives was also detected: phenol, phenoxyethanol, 4-propylguaiacol (dihydroeugenol), 2-methoxy-4-methylphenol (*p*-cresol), 2-phenylethanol, benzyl alcohol, and furfuryl alcohol.

The high relative amounts of short-chain free fatty acids (lactic, acetic, propionic, and decanoic acids) and alcohols (i.e., ethanol, 1,2-propanediol) produced by fermentation, accompanied by the acidic conditions and temperature increases (to 28 °C after 3 d of fermentation) during the first phase of ensiling, help explain the large number of esters (*n* = 72 congeners) found in all the treatments (see Table 2). The presence of lactate and acetate esters in grass and maize silages was also reported by Weiss, Kroschewski, Auerbach,^8^ and Weiss.^31^

Interestingly, the ligninocellulosic fiber degradation products formed by oxidative processes on phenolic acids, such as 4-propylguaiacol (dihydroeugenol), 2-methoxy-4-methylphenol (*p*-cresol), 1,3-dimethoxybenzene (3-methoxyanisole), and 4-vinylguaiacol, which were below the method’s detection limit in the herbage prior to fermentation, were up-modulated by all the treatments for both the LOW and HIGH DM silages. Mishra et al. reported the formation of 4-vinylguaiacol from ferulic acid by yeasts and *Bacillus* species during beer and wine fermentation.^32^ Moreover, some aromatic esters (i.e., methyl benzoate, methyl salicylate, and congeners) with known antifungal and antimicrobic activity increased in the silages after fermentation. Such esters have been used in some studies as silage additives to improve aerobic stability (e.g., Da Silva Pinto^29^).

### Unrevealing the Diagnostic Patterns of Volatiles and Their Correlation with Inocula

In order to delineate the existing strong correlations between the multiple chemical dimensions within the VOCs patterns, a Pearson correlation test was conducted on response data from all the UT features showing meaningful variations within sample classes (Fisher ratio *F*_crit_ (4, 8) = 3.84 with α = 0.5). Therefore, all the UT features with *F*_calc_ > 4 were included. The resulting correlation matrix (157 features × 35 samples) is rendered as a heat map in Supporting Information Figure 1 – SF1. Hierarchical clustering, applied to both columns and rows, helps to highlight patterns with strong intercorrelations within samples. As expected, the ester derivatives showed strong correlation (*r* ≥ 0.900 and significance level α = 0.05) with their respective acid/alcohol moieties. These correlations are highlighted with black squares in Supporting Information Figure 1 – SF1. For example, the Pearson correlation value *r* for 1-propanol and propionic acid was 0.9605 (*p* < 0.0001), thus confirming their common biosynthetic formation pathway; at the same time, corresponding esters like ethyl propionate, propyl propionate, and propyl palmitate showed *r* values greater than 0.950 with acid/alcohol moieties and with each other.

However, the fatty acid hydroperoxide cleavage products, likely formed by hydroperoxide liases in the herbage at ensiling [e.g., (*E*)-2-pentenal; hexanal; (*E*)-2-hexenal; (*E,Z*)-2,4-hexadienal; heptanal; (*E*)-2-heptenal; (*E*)-2-octenal; 1-octen-3-ol; (*E*)-2-octen-1-ol; (*E*)-2-decenal; and (*E,Z*)-2,4-decadienal] did not show any strong correlation (*r* > 0.9000) with any known LAB fermentation products. This evidence is quite reasonable if the chemical signatures are considered in light of their biological phenomena. The effect of microbial transformation on primary plant metabolites dominates the native volatile signatures to a great extent.

**Figure 2:**
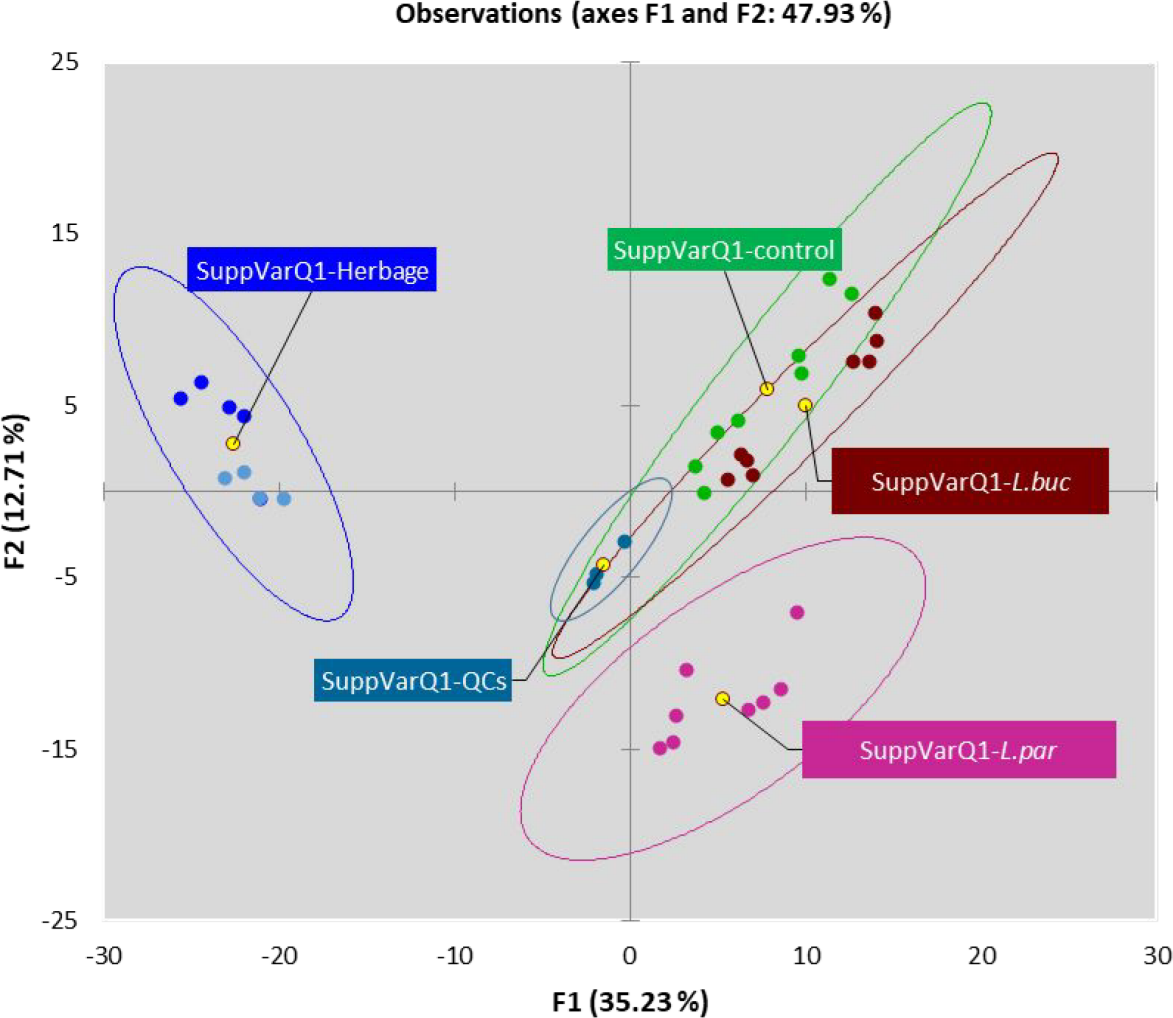
PCA scores plot based on absolute responses from UT components (452 × 35) (component features × samples). Natural samples groups almost independently clustered, as shown by confidence ellipses (95% of confidence level), correspond to herbage prior to fermentation (HIGH DM dark blue indicators – LOW DM light blue indicators) and to fermented silage samples (pink – *Lpar*, purple – *Lbuc*, and green – controls). QC samples (QC) are also reported. Yellow circles locate group centroids.

The primary interest of this study was to delineate volatile patterns pertaining to specific fermentation microorganisms, and a first unsupervised exploration of the complete data matrix was therefore run by means of principal component analysis (PCA). The considered data included absolute responses from UT components (452 × 35) (component features × samples), and mean centering and normalization were performed prior to PCA elaboration. The obtained results, shown in the score plot on PC1 versus PC2 in Figure 2, suggest a clear impact of the fermentation microorganisms on the total detectable volatilome. The natural sample groups are clustered almost independently, as also shown by the confidence ellipses (95% of confidence level), and correspond to herbage at ensiling (HIGH DM and LOW DM blue indicators) and to fermented silage samples (*Lpar* pink, *Lbuc* purple, and CON green indicators) discriminated along the PC1 (35.23% of the total explained variance). In the fermented samples, which in turn were clustered independently along the PC2 (12.71% of the total variance), *Lpar* was connoted by a distinctive pattern of volatiles, compared to the *Lbuc* and CON samples, which likely overlapped. The PCA elaboration clearly shows that the fermetative profiles of CON and *Lbuc* silages were similar, thus suggesting that the control (CON) underwent heterolactic fermentation, especially for the LOW silages (data not shown). This evidence is in keeping with the absence of statistical differences in aerobic stability between the CON and *Lbuc* in the LOW silages (Table 1). It should be noted that the aerobic stability values were greater than those of the homolactic fermented maize silages (with a lactic-to-acetic acid ratio >4.5) with a similar conservation time, which generally ranged from 25 to 95 h of aerobic stability.^3,34^

The set of UT features was then sieved to extract those with a statistically relevant variability between all the sample classes. The criterion was driven by the Fisher test; those with an *F*_calc_ < *F*_crit_; with *F*_crit_ (4, 8) = 3.84 (α = 0.5) were excluded from any further computations. The *F*_calc_ values for all the UT features are reported in Supporting Information Table 2.

**Figure 3:**
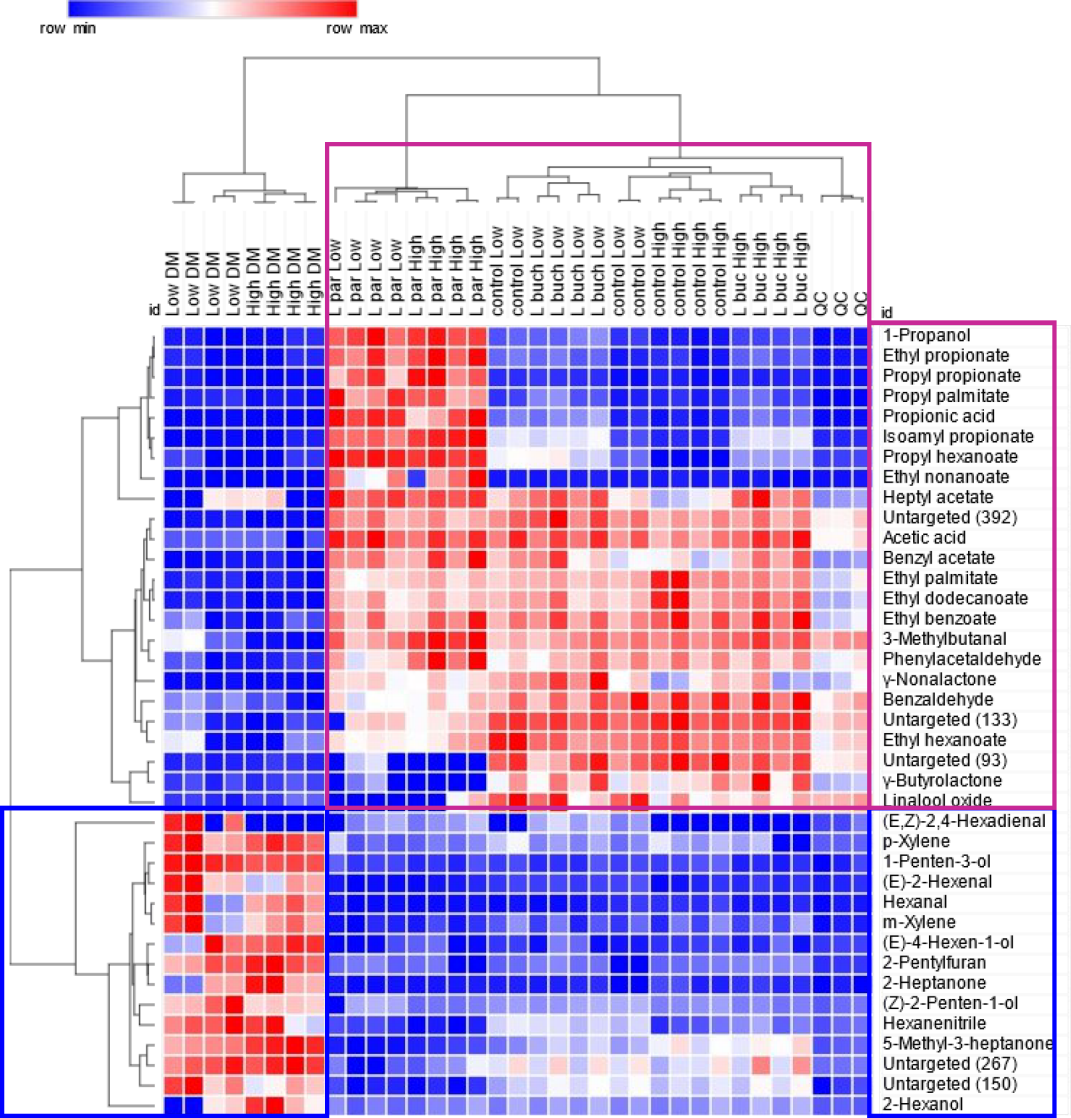
Heat-map visualization based on absolute analytes responses (*F* > 30 all classes) after normalization by Z-score (subtract mean and divide by standard deviation). Hierarchical clustering is by Pearson similarity matrix, colorization is by a blue-red scale (row min/blue–row max/red). HIGH and LOW DM are reported together with samples’ characteristics.

The 1-penten-3-ol alcohol (F 535), followed by 1-propanol (397), ethyl propionate (F 234), propyl palmitate (F 211), propyl propionate (F 210), and propionic acid (152.71) were the most informative volatiles detected in all the analyzed samples and were connoted by a great and meaningful discrimination power between sample classes (i.e., *F*_calc_ > 30 all the classes). In particular, 1-penten-3-ol, together with a series of C6 derivatives formed by lipoxygenase hydroperoxy liase activity [e.g., (*E,Z*)-2,4-hexadienal, (*E*)-4-hexen-1-ol, (*E*)-2-hexenal, hexanal, and 2-hexanol], showed a higher abundance in herbage at ensiling (HIGH and LOW DM samples), as also illustrated in the heat map in Figure 3. The heat map is based on absolute analyte responses after normalization by the *Z*-score (subtracted mean and divided by the standard deviation); Pearson’s similarity matrix was used for the hierarchical clustering.

The herbage at ensiling was also characterized by a distinctive xylene pattern (1,4-dimethyl benzene/*p*-xylene, and 1,3-dimethyl benzene/*m*-xylene); their presence, which has never before been reported in studies focused on silage VOCs, deserves further investigations to exclude the possibility of environmental contamination. Their reduction in silages, where their relative abundance was on average 0.3 to 0.6 of that of the corresponding herbage prior to fermentation, is of particular interest.

*Lpar* has a strong and distinctive signature that is dominated by propionic acid and 1-propanol; characteristic volatiles, as also confirmed by the quantitative HPLC data (Table 1), form an independent cluster in Figure 3 (purple square). Several corresponding esters can be observed: ethyl propionate, propyl propionate, propyl palmitate, isoamyl propionate, and ethyl nonanoate. The predominance of propionic esters is in keeping with the existing knowledge on silage volatile signatures, as reported by Hafner et al.^9^ and Lee et al.^31^ in whole oat flour fermented by *Lcb. paracasei*. However, the large chemical diversity of the detected esters far exceeds the previously documented diversity.

The impact of *Len. Buchneri* fermentation, which generated a volatile pattern that is not clearly distinguishable from the CON samples, is instead connoted by the presence of several ethyl esters (i.e., ethyl propionate, ethyl nonanoate, ethyl palmitate, ethyl dodecanoate, and ethyl benzoate). This suggests that a relevant amount of ethanol produced from fermentation led to ester formation and was favored by the presence of organic acids in acidic conditions. The presence of acetic acid and acetates (i.e., benzyl acetate and heptyl acetate), whose formation is coherent with the heterolactic fermentation acted by bacteria of *Lentilactobacillus* genus,^36^ is also relevant.

### Visual Feature Fingerprinting Used to Promptly Highlight VOCs Diagnostic Patterns

The successive data elaboration step was aimed at highlighting the distinctive fermentation patterns induced by lactic acid bacteria on herbage at ensiling, obtained by means of *visual feature fingerprinting.*(14) The approach performs a pairwise image comparison, through the use of a dedicated algorithm that computes the difference for each data point (i.e., the output of the detector at a point in time) between pairs of chromatograms. These differences are then mapped in a Hue-Intensity-Saturation (HIS) color space to create an image of the relative differences between image pairs in the retention-times plane.^37^ The procedure is fully automated, and when applied after UT fingerprinting, it provides information about variations in targeted or untargeted features between pairwise compared samples. In this application, visual comparisons were performed on composite class images^28^ generated by combining 2D chromatograms from samples belonging to the class (e.g., herbage at ensiling, *Lbuc*, *Lpar*, *control* CON). Details of the application can be found in the *experimental section*.

The example in Supporting Information Figure 2A – SF2A refers to an *Lpar* composite-class image and was considered as the *analyzed* class image compared with the raw silage considered as the *reference*. The resulting image is rendered as a “colorized fuzzy ratio”; the difference between the aligned class images is computed at each data point and colored green when positive (larger detector response in the *analyzed* image, i.e., samples fermented by *Lcb. paracasei*) or red when the difference is negative (larger detector response in the *reference* image, i.e., unfermented herbage). The brightness in the image depends on the magnitude of the response; white saturation indicates pixels/regions with detector responses that are nearly equal to the pair images.

The data point differences (red or green colored pixels) combined with response data from the UT features indicate that several analytes, including some untargeted ones, were likely, if not exclusively, produced by *Lcb. paracasei* fermentation and were not detectable in the herbage prior to fermentation. These volatile metabolites may be considered the result of a specific transformation pathway of *Lcb. paracasei*. The most abundant volatiles were: propyl acetate, isoamyl lactate, ethyl lactate, propyl pentanoate, propyl phenylacetate, and propyl laurate. Degradation products of phenolic acids were also formed: 2-methoxy-4-propylphenol (isoeugenol), 4-propylguaiacol, and *p*-cresol. These components, connoted by a strong phenolic odor, could have an impact on the sensory properties of silage but could also exert a protective effect against molds such as *Aspergillus parasiticus.*(38)

**Figure 4:**
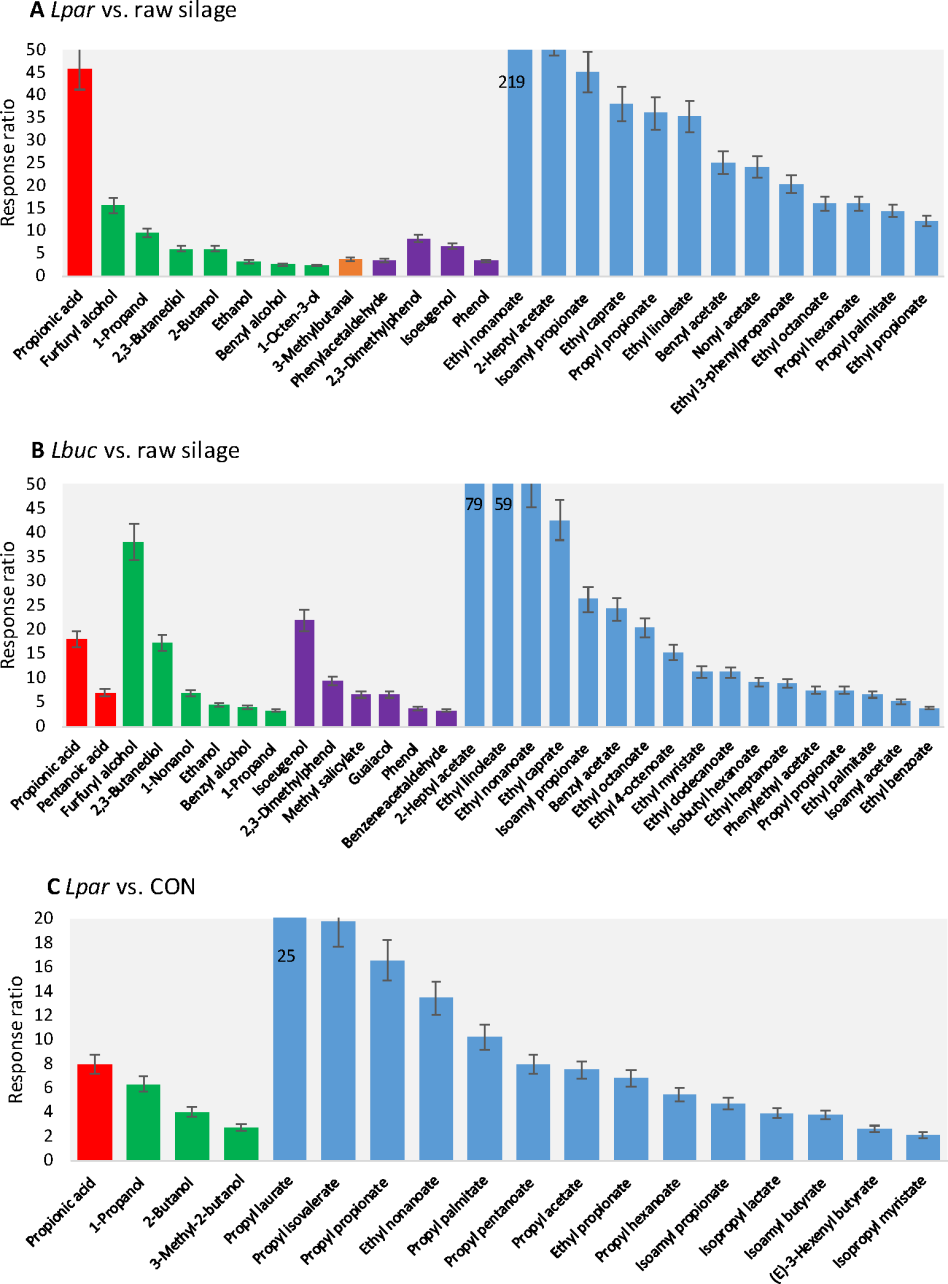
Response ratios variation between pairwise cumulative chromatograms highlighting up-regulated target analytes. Bar coloring refers to chemical classes (*red* carboxylic acids; *green* alcohols; *orange* aldehydes; *purple* phenols; *cyano* esters). *Lbc*. *paracasei* vs herbage prior ensiling (**4A**); *Lbc. buchneri* vs herbage prior ensiling (**4B**); *Lbc*. *paracasei* vs control CON (**4C**).

A selection of volatiles already detected in the herbage prior to ensiling, but largely up-regulated by *Lcb. paracasei* fermentation, are shown in the histogram in Figure 4A. Analytes, grouped according to their chemical classes (differently colored histogram bars), are reported in descending order of the response ratios (i.e., the ratio between the absolute 2D peak volumes). For all these components, *F*_calc_ > *F*_crit_.

Propanoic acid (*F*_calc_ 247) was the most variable organic acid, with a 46-fold increase over the herbage at ensiling; accordingly, 3-methyl-1-butyl propanoate (isoamyl propionate – *F*_calc_ 313) was 45 times more abundant in the fermented samples. Furfuryl alcohol (2-furanmethanol – *F*_calc_ 101) showed a 15-fold increase, while 1-propanol (*F*_calc_ 907) had a 9-fold increase. Ethyl nonanoate (*F*_calc_ 19) was 219 times higher in *Lcb. paracasei* samples than in the herbage prior to fermentation. Nonanoic acid and its ethyl ester have already been found in *Lcb. paracasei* fermentation products.^35,39^

The comparative visualization shown in Supporting Information Figure 2B – SF2B highlights pattern differences between composite-class images obtained by combining 2D chromatograms of *Lbuc* silage samples versus unfermented herbage. An inspection of the response data and the relative supervised statistics (Fisher ratio *Lbuc* vs herbage prior to ensiling) indicates that several targeted compounds in the fermented silages were below the method’s detection limit. Such compounds could be considered characteristic yet unique components of unfermented samples. Among these compounds, several carbonylic derivatives were identified: (*Z*)-2-hexenal; (*E*)-3-hexenal; (*E,E*)-2,4-nonadienal, (*Z*)-2-undecenal; pentadecanal; 2-propanone; 2-butanone; 2-pentanone; (*E,E*)-3,5-octadien-2-one; 2-nonanone; 2-undecanone. Their presence suggests an extensive lipoxygenase hydroperoxy liase activity, likely as the result of the activity of plant endogenous enzymes. Some of the compounds were also in the subgroup of most informative components for maize herbage prior to fermentation, as shown in Figure 3.

Several analytes were also detected in the fermented samples, and those with the highest responses were: propyl acetate; diethyl succinate; ethyl sorbate; ethyl lactate; isoamyl lactate, and *p*-cresol. Lactic and acetic acid esters, derived from esterification of the main organic acids produced by heterolactic fermentation,^40^ were the most abundant esters and showed the highest percentage of the total response. As far as the statistically meaningful variations (*F*_calc_ > *F*_crit_) are concerned, the histogram in Figure 4B illustrates the fold-change of a selection of targeted compounds ordered according to their chemical class. Propionic (*F*_calc_ 147) and pentanoic acid (*F*_calc_ 12) resulted to be 18 and 7 times more abundant, respectively, in silage fermented by *Lbuc* than in the original herbage. The larger amount of several aromatic derivatives, as also discussed in section “Compositional Complexity of the Volatilome and the Major Chemical Classes”, could have beneficial effects on the microbial and yeast stability of silage. Isoeugenol, guaiacol, 2,3-dimethylphenol, methyl salicylate, and benzeneacetaldehyde, some of the most up-regulated phenols, are worth mentioning. In particular, isoeugenol had a 22-fold increase, compared to the original herbage. Eugenol and its derivatives are known to have antimicrobial activity against yeast (i.e., *Saccharomyces cerevisiae*)^41^ and the fungi of *Aspergillus* genus,^38^ with a lower minimal inhibitory concentration for iso-eugenol. Moreover, eugenol and its derivatives have also been studied because of their inhibition effect on the capacity of molds to produce mycotoxins.^42,43^ Those with a higher statistical relevance on average showed a 20-fold increase for ethyl esters and dominated the others (e.g., ethyl linoleate 59-fold; ethyl nonanoate 50-fold; and ethyl caprate 43-fold increase).

The different impact of *Lcb. paracasei* fermentation over the *control* samples, where epiphytic microbiota dominated fermentation, is shown in Supporting Information Figure 2C – SF2C, where composite-class images of *Lcb. paracasei* samples are compared with cumulative chromatographic patterns of *control* samples. The observed compositional differences can mainly be ascribed to the inoculation of *Lcb. paracasei*, which affected fermentation through a strongly heterolactic pathway with a relevant production of acetic acid, propionic acid, and 1-propanol (Table 1). The latter formed from the secondary degradation of acetic acid and 1,2-propandiol caused by such bacteria of the *Lentilactobacillus* genus as *Len. Diolivorans.*(29)

The volatiles that show the most meaningful variation between *Lpar* and CON samples (shown in the histogram in Figure 4C) are propionic acid, with an eightfold increase (*F*_calc_ 150), followed by 1-propanol (sixfold increase), 2-butanol (fourfold increase), and 3-methyl-2-butanol (threefold increase). The esters, which are dominated by 1-propanol derivatives, on average show a 10-fold increase. Compared to *control* samples, the silage volatilome impacted by *Lcb. paracasei* shows a distinctive signature dominated by propionic acid and 1-propanol derivatives.

The great chemical complexity of fermented silage VOCs, explored by GC × GC-TOF MS, adds knowledge on the metabolic pathways triggered by specific bacterial strains and offers further interpretation keys that can be used to obtain a better understanding of silage stabilization mechanisms against the degradative action of yeasts and molds during the exposure of silage to air. This study, by combining well-established chemical characterization protocols and marker compound monitoring, has enabled a robust cross-validation of data derived from comprehensive VOCs fingerprinting, based on HS-SPME sampling. Although not quantitative *per se*, the approach promptly reveals pattern variations over a large dynamic range of concentrations and provides evidence on the activation of metabolic pathways and on the synergies between bacterial strains.

Moreover, by resorting to untargeted/targeted fingerprinting, it was possible to monitor several chemical classes, while the up-/down-regulation of single analytes was easily tracked over many samples by multivariate statistics and dedicated data processing (i.e., visual feature fingerprinting).

## Supporting Information Available

Metadata [retention times ^*1*^*t*_R_, ^*2*^*t*_R_, corresponding precision data as %RSD (*n* = 35), and experimental linear retention index (*I*^*T*^)], average response over samples’ classes, and precision data (% RSD) on QCs features volumes; Target components and untargeted features mapped through all analyzed samples. Target analytes, reported with corresponding CAS registry number, were identified according to criteria of spectral similarity (DMF above 900 and RMF above 950) and *I*^*T*^ tolerance of ±15 units. Analytes are listed with retention times (^*1*^*t*_R_, ^*2*^*t*_R_) and corresponding precision data expressed as %RSD across all analyses (*n* = 35), experimental linear retention index (*I*^*T*^) and tabulated *I*^*T*^ (NIST database https://webbook.nist.gov/chemistry/), Fisher ratio (*F*) values calculated for all classes (F all). When features were invariant (e.g., undetected) within a class, the Fisher ratio cannot be computed and in table is reported as “ND”. This table complements Table 2 of the manuscript. Pearson correlation matrix obtained from absolute response data corresponding to UT features with a F calc >4 for all classes. Hierarchical clustering is based on Pearson correlation while heat map colorization ranges from blue (−1) to red (1) *r* values. Black squares highlight features cluster with a strong correlation. Comments are reported in the text; Comparative visualization between composite class images obtained by summing 2D chromatograms from samples belonging to the same class. In **SF2A** the *analyzed* image is the composite-class chromatogram from *L. par* fermented samples while as *reference* is taken the composite class image from all herbage samples. In **SF2B** the *analyzed* image is from *L. buc* while the *reference* is that from herbage samples. In **SF2C** the *analyzed* is that from *L par* samples compared to the *reference* from control samples. The comparative visualization is rendered as “colorized fuzzy ratio”; the difference at each data point between aligned pairwise images is computed and colored green, when positive (larger detector response in the *analyzed* image) or colored red, when negative (larger detector response in the *reference* image) (PDF)
