# Supplementary material for: Effect of Inoculation with Lentilactobacillus buchneri and Lacticaseibacillus paracasei on the Maize Silage Volatilome: The Advantages of Advanced 2D-Chromatographic Fingerprinting Approaches
Source: J Agric Food Chem. 2022 Sep 14;70(38):12232–48. doi: 10.1021/acs.jafc.2c03652 (PMC9523707; doi:10.1021/acs.jafc.2c03652)

Supporting Information

Effect of inoculation with *Lentilactobacillus buchneri* and *Lacticaseibacillus paracasei* on maize silage volatilome: advantages of advanced 2D-chromatographic fingerprinting approaches

Simone Squara[1$], Francesco Ferrero[2$], Ernesto Tabacco[2], Chiara Cordero[1*], Giorgio Borreani[2]

[$]Simone Squara and Francesco Ferrero contributed equally to this work

Authors' affiliation:

[1]University of Turin, Dipartimento di Scienza e Tecnologia del Farmaco, Turin, Italy

[2]University of Turin, Department of Agriculture, Forest and Food Sciences (DISAFA), Grugliasco (TO), Italy

Contents

Supplementary Table 1: Features metadata [retention times $^{1}t_R$, $^{2}t_R$, corresponding precision data as %RSD (n=35), and experimental linear retention index (I^T)], average response over samples' classes, and precision data (% RSD) on QCs features volumes (Table S1);

Supplementary Table 2: Target components and untargeted features mapped through all analyzed samples. Target analytes, reported with corresponding CAS registry number, were identified according to criteria of spectral similarity (DMF above 900 and RMF above 950) and I^T tolerance of ± 15 units. Analytes are listed with retention times ($^{1}t_R$, $^{2}t_R$) and corresponding precision data expressed as %RSD across all analyses (n=35), experimental linear retention index (I^T) and tabulated I^T (NIST database https://webbook.nist.gov/chemistry/), Fisher ratio (*F*) values calculated for all classes (F all). When features were invariant (e.g., undetected) within a class, the Fisher ratio cannot be computed and in table is reported as "ND". This table complements Table 2 of the manuscript.

Supplementary Figure 1 – SF1: Pearson correlation matrix obtained from absolute response data corresponding to UT features with a F calc > 4 for all classes. Hierarchical clustering is based on Pearson correlation while heat map colorization ranges from blue (-1) to red (1) *r* values. Black squares highlight features cluster with a strong correlation. Comments are reported in the text (Figure S1);

Supplementary Figure 2 – SF2: Comparative visualization between composite class images obtained by summing 2D chromatograms from samples belonging to the same class. In **SF2A** the *analyzed* image is the composite-class chromatogram from *L. par* fermented samples while as *reference* is taken the composite class image from all herbage samples. In **SF2B** the *analyzed* image is from *L. buc* while the *reference* is that from herbage samples. In **SF2C** the *analyzed* is that from *L par* samples compared to the *reference* from control samples. The comparative visualization is rendered as "colorized fuzzy ratio"; the difference at each data point between aligned pair-wise images is computed and colored green, when positive (larger detector response in the *analyzed* image) or colored red, when negative (larger detector response in the *reference* image) (Figure S2).

Supplementary Table 1: Features metadata [retention times ${}^{1}t_R$, ${}^{2}t_R$, corresponding precision data as %RSD (n=35), and experimental linear retention index (I^T)], average response over samples' classes, and precision data (% RSD) on QCs features volumes.

						Average 2D peak volumes						
**Feature ID**	**${}^{1}t_R$ min**	**%RSD**	**${}^{2}t_R$ s**	**%RSD**	**I^T exp**	**Raw low DM**	**Raw high DM**	**Controls**	**L buchneri**	**L paracasei**	**QCs**	***RSD% QC***
(E)-1,3-Octadiene	7.80	0.76	1.27	4.28	989	1.44E+06	2.44E+06	2.28E+05	ND	ND	1.43E+05	*29.03*
(E)-2-Decenal	27.45	0.68	1.20	3.40	1643	5.37E+06	4.72E+06	1.27E+07	1.38E+07	1.25E+07	8.96E+06	*12.47*
(E)-2-Hepten-1-ol	23.61	1.13	0.75	9.91	1507	1.04E+07	1.76E+07	6.70E+06	5.44E+06	6.70E+06	2.60E+06	*16.50*
(E)-2-Heptenal	18.42	1.02	1.05	3.69	1331	2.11E+07	1.22E+07	2.80E+07	3.26E+07	2.02E+07	2.53E+07	*14.12*
(E)-2-Hexen-1-ol	20.86	1.31	0.67	5.19	1412	4.60E+06	3.44E+06	7.59E+06	3.35E+06	6.76E+05	1.71E+06	*6.59*
(E)-2-Hexenal	15.15	1.10	0.98	4.21	1226	1.44E+06	8.00E+05	ND	1.12E+06	ND	ND	*ND*
(E)-2-Nonenal	24.58	0.85	1.17	2.86	1543	6.66E+06	5.34E+06	7.14E+06	5.53E+06	ND	9.08E+06	*5.03*
(E)-2-Octen-1-ol	26.52	0.68	0.75	4.96	1613	1.50E+07	2.12E+07	1.40E+07	1.27E+07	9.22E+06	6.78E+06	*10.04*
(E)-2-Octenal	21.56	0.81	1.10	3.50	1436	4.89E+07	2.66E+07	1.69E+07	3.18E+07	2.74E+07	1.76E+07	*10.09*
(E)-2-Penten-1-ol	17.88	1.02	0.63	5.98	1313	7.72E+06	1.32E+07	2.09E+06	1.72E+06	3.25E+06	7.57E+05	*7.37*
(E)-2-Pentenal	12.40	1.19	0.87	4.31	1137	5.11E+06	8.54E+06	7.24E+06	1.10E+07	8.95E+06	6.57E+06	*10.36*
(E)-2-Undecenal	30.33	0.47	1.24	3.54	1745	3.93E+06	3.17E+06	9.50E+05	ND	7.88E+05	1.33E+06	*17.62*
(E)-3-Hexen-1-ol	20.07	0.94	0.69	5.36	1385	2.14E+07	2.02E+07	2.10E+07	2.15E+07	1.59E+07	1.60E+07	*9.31*
(E)-3-Hexenal	12.83	0.00	0.89	4.62	1151	5.06E+06	1.04E+06	ND	ND	ND	ND	*ND*
(E)-3-Hexenyl acetate	18.02	0.95	1.07	3.94	1318	4.34E+06	2.55E+06	5.57E+06	1.10E+07	1.51E+07	8.27E+06	*1.56*
(E)-3-Hexenyl butyrate	22.53	0.97	1.30	5.05	1470	1.02E+07	1.39E+07	6.38E+06	7.43E+06	3.12E+07	6.71E+06	*6.29*
(E)-3-Hexenyl lactate	29.45	0.63	1.22	3.76	1709	2.74E+06	6.46E+05	5.97E+06	5.41E+06	5.15E+06	2.38E+06	*11.99*
(E)-4-Hexen-1-ol	20.76	0.76	0.67	5.51	1408	2.34E+07	3.26E+07	5.78E+06	5.54E+06	4.34E+06	5.97E+06	*8.30*
(E)-Theaspirane	23.72	0.74	1.64	2.65	1511	ND	ND	9.78E+06	1.05E+07	8.44E+06	5.47E+06	*7.45*
(E,E)-2,4-Heptadienal	22.52	0.84	0.89	4.28	1469	1.75E+07	7.74E+06	1.89E+07	1.88E+07	1.89E+07	1.48E+07	*8.33*
(E,E)-2,4-Nonadienal	26.06	0.58	1.01	3.23	1597	1.03E+07	3.73E+06	ND	ND	ND	ND	*ND*
(E,E)-3,5-Octadien-2-one	25.61	0.17	0.92	1.92	1581	1.45E+07	9.69E+06	ND	ND	ND	ND	*ND*
(E,Z)-2,4-decadienal	31.35	1.37	1.03	3.97	1786	2.58E+06	1.54E+06	2.98E+06	3.77E+06	5.70E+06	5.56E+05	*12.03*
(E,Z)-2,4-heptadienal	23.34	0.77	0.88	4.42	1498	3.42E+07	2.54E+07	2.73E+07	2.73E+07	2.55E+07	1.42E+07	*12.80*
(E,Z)-2,4-Hexadienal	20.76	0.84	0.82	4.98	1409	8.17E+06	ND	ND	2.26E+06	3.18E+06	2.10E+06	*12.37*
(E,Z)-2,6-Nonadienal	29.09	0.21	0.98	3.77	1695	2.74E+06	ND	ND	8.66E+05	ND	ND	*ND*
(E,Z)-3,5-Octadien-2-one	24.21	0.18	0.93	2.79	1529	2.23E+07	1.81E+07	ND	ND	ND	ND	*ND*
(Z)-2-Hexen-1-ol	20.66	1.43	0.69	7.27	1405	ND	ND	6.28E+06	5.05E+06	4.23E+06	7.18E+05	*29.25*
(Z)-2-Hexenal	14.78	0.20	0.96	3.85	1214	1.79E+07	1.15E+07	ND	ND	ND	ND	*ND*
(Z)-2-Octenal	20.91	0.26	1.10	2.89	1414	2.88E+06	2.33E+06	ND	ND	ND	ND	*ND*
(Z)-2-Penten-1-ol	18.10	1.04	0.63	5.96	1320	1.47E+07	1.21E+07	7.06E+06	7.06E+06	6.41E+06	5.89E+06	*8.19*
(Z)-3-Hexenal	13.13	0.80	0.88	5.38	1161	3.28E+06	1.89E+06	ND	ND	ND	ND	*ND*
(Z)-3-Hexenyl acetate	18.25	1.08	1.06	3.68	1325	2.45E+07	1.71E+07	2.83E+07	3.63E+07	3.04E+07	2.45E+07	*6.81*
(Z)-3-Hexenyl benzoate	38.75	0.45	1.00	4.24	2112	2.35E+06	1.51E+06	2.59E+06	2.17E+06	1.77E+06	9.28E+05	*15.17*
(Z)-3-Hexenyl hexanoate	27.80	0.67	1.39	3.19	1654	3.54E+06	1.39E+06	3.07E+06	3.23E+06	3.62E+06	1.36E+06	*6.77*
(Z)-3-Nonen-1-ol	28.51	0.18	0.81	2.86	1677	1.63E+07	1.89E+07	ND	ND	ND	ND	*ND*
(Z)-6-Octen-2-one	18.73	0.48	1.06	3.24	1341	3.75E+06	3.16E+07	8.91E+06	5.23E+06	6.06E+06	ND	*ND*
1,2,4-Trimethylbenzene	17.07	1.00	1.12	3.44	1287	3.87E+06	4.16E+06	4.88E+06	5.85E+06	4.43E+06	3.23E+06	*6.83*
1,2-Propanediol, 2-acetate	26.55	0.73	0.60	7.24	1613	ND	ND	ND	1.61E+07	8.17E+06	3.04E+06	*4.04*
1,3-Dimethoxybenzene	30.08	0.63	0.78	4.97	1735	1.09E+07	1.45E+07	8.51E+06	7.83E+06	6.21E+06	3.60E+06	*8.87*
1-Butanol	12.68	1.18	0.64	5.51	1146	5.51E+06	7.65E+06	7.99E+06	4.64E+06	7.60E+06	2.89E+05	*28.00*
1-Decanol	30.24	0.64	0.90	4.33	1741	2.99E+06	2.90E+06	7.44E+06	6.48E+06	4.18E+06	3.06E+06	*13.00*
1-Ethyl-2-methylbenzene	15.43	1.00	1.16	4.08	1235	3.98E+06	4.63E+06	ND	1.82E+06	7.05E+05	2.80E+06	*14.81*
1-Hexanol	19.07	1.05	0.73	5.13	1352	7.68E+07	1.26E+08	1.15E+08	8.19E+07	6.41E+07	6.84E+07	*7.27*
1-Nitrohexane	23.86	0.18	0.94	3.38	1517	2.28E+07	1.63E+07	ND	ND	ND	ND	*ND*
1-Nitropentane	20.78	0.97	0.88	4.05	1409	1.40E+07	1.31E+07	ND	1.07E+06	ND	6.53E+05	*7.36*
1-Nonanol	27.63	0.67	0.85	4.90	1648	4.65E+06	ND	1.88E+07	1.60E+07	1.59E+07	8.91E+06	*12.36*
1-Octanol	24.97	0.77	0.81	5.07	1557	1.36E+07	1.91E+07	9.74E+06	1.94E+07	2.09E+07	8.50E+06	*6.36*
1-Octen-3-ol	21.99	0.85	0.78	4.76	1451	2.92E+07	3.08E+07	3.31E+07	6.09E+07	6.89E+07	5.13E+07	*4.99*
1-Pentanol	15.95	1.12	0.69	5.40	1251	2.39E+07	3.06E+07	2.05E+07	1.88E+07	1.57E+07	1.28E+07	*13.87*
1-Penten-3-ol	13.23	1.28	0.64	6.27	1164	1.89E+07	1.74E+07	6.85E+06	5.88E+06	6.36E+06	5.56E+06	*19.29*
1-Penten-3-one	9.33	1.22	0.80	5.36	1042	5.80E+06	4.04E+06	3.82E+06	7.82E+06	ND	3.45E+06	*6.18*
1-Propanol	9.66	1.16	0.59	6.48	1052	9.29E+06	1.20E+07	1.07E+07	2.61E+07	1.01E+08	8.92E+06	*9.09*

1-Tetradecanol	39.78	0.44	1.06	3.68	2159	1.39E+06	2.74E+06	7.53E+06	7.39E+06	6.15E+06	1.80E+06	16.44
1-Tetradecene	21.84	1.21	2.46	2.49	1446	2.56E+06	2.93E+06	4.01E+06	1.62E+06	1.70E+06	2.61E+06	3.95
1-Undecene	12.67	1.03	2.36	1.81	1146	7.59E+05	8.24E+05	4.80E+05	5.69E+05	ND	2.89E+05	11.33
2,2,6-Trimethylcyclohexanone	18.27	1.03	1.31	3.03	1326	1.33E+07	1.03E+07	3.97E+07	1.28E+07	4.60E+06	1.20E+07	7.89
2,3,3a,4,5,7a-Hexahydro-3,6-dimethylbenzofuran	23.95	0.75	1.22	3.33	1520	ND	ND	3.13E+06	3.46E+06	1.46E+06	1.65E+06	7.30
2,3-Butanediol	25.38	0.72	0.55	6.89	1572	1.65E+07	1.08E+07	1.14E+07	1.20E+07	6.93E+06	6.06E+06	3.65
2,3-Dimethylphenol	39.50	0.45	0.60	6.01	2146	1.42E+07	3.92E+06	1.12E+08	8.77E+07	7.51E+07	4.56E+07	7.27
2,3-Pentanedione	10.56	0.00	0.78	0.00	1079	5.50E+07	1.56E+07	3.52E+06	4.03E+06	3.42E+06	1.43E+06	12.40
2,4-Hexadien-1-ol	25.89	0.20	0.61	3.79	1591	4.77E+06	3.41E+06	ND	ND	ND	ND	ND
2-Butanol	9.30	1.22	0.64	5.89	1041	5.64E+06	6.49E+06	7.94E+06	1.39E+07	3.64E+07	5.88E+06	9.69
2-Butenal	9.94	1.31	0.75	7.01	1060	9.40E+05	2.26E+05	1.66E+05	1.58E+05	ND	6.29E+05	5.47
2-ethyl-3-methyl maleimide	41.56	0.41	0.62	5.80	2250	2.84E+06	3.23E+06	4.56E+06	1.07E+06	1.19E+06	1.16E+06	9.76
2-Ethylfuran	7.78	0.90	0.79	5.75	987	7.12E+06	6.34E+06	ND	7.25E+05	ND	ND	ND
2-Ethylhexanol	23.17	0.84	0.82	5.84	1492	4.23E+06	ND	ND	2.58E+06	2.23E+06	2.18E+06	16.35
2-Heptanol	18.08	1.08	0.82	4.50	1320	1.91E+07	2.29E+07	1.41E+07	1.65E+07	1.28E+07	1.16E+07	9.74
2-Heptanone	14.00	1.13	1.13	3.17	1189	4.41E+07	7.92E+07	1.02E+07	7.81E+06	3.71E+06	1.91E+06	16.22
2-Heptyl acetate	16.55	1.06	1.39	3.53	1270	ND	3.12E+05	7.15E+06	1.07E+07	7.10E+06	4.03E+06	19.41
2-Hexanol	15.04	1.17	0.76	5.70	1222	3.04E+06	7.99E+06	2.96E+06	3.27E+06	2.55E+06	1.92E+06	5.96
2-Methoxy-4-methylphenol	34.88	0.54	0.68	6.03	1936	ND	ND	9.85E+07	1.11E+08	7.50E+07	3.32E+07	11.26
2-Methyl-2-pentenal	13.46	1.24	0.97	5.30	1171	6.91E+06	ND	ND	ND	2.03E+05	ND	ND
2-Methyl-3-pentanol	13.13	1.25	0.79	5.18	1161	1.78E+06	1.36E+07	1.55E+07	1.70E+07	1.47E+07	1.14E+07	11.45
2-Methyl-3-pentanone	8.72	1.26	1.04	4.09	1023	4.63E+06	6.38E+06	3.83E+06	4.55E+06	5.21E+05	1.54E+06	0.46
2-Methylfuran	6.24	0.00	0.66	0.00	846	ND	1.96E+06	ND	ND	ND	ND	ND
2-Nonanone	20.49	0.52	1.28	1.91	1399	1.90E+07	2.19E+07	5.40E+06	ND	ND	ND	ND
2-Octanol	21.07	0.86	0.87	4.23	1419	3.93E+06	6.29E+06	7.56E+06	8.64E+06	6.51E+06	4.24E+06	9.02
2-Octanone	17.26	1.15	1.23	2.62	1293	2.39E+07	5.28E+07	5.55E+06	1.61E+06	ND	1.18E+06	7.97
2-Pentanone	8.28	0.96	0.87	2.96	1010	7.33E+07	8.75E+07	4.20E+07	ND	ND	ND	ND
2-Pentyl acetate	10.80	1.23	1.15	3.71	1087	3.47E+06	7.53E+06	1.22E+07	1.24E+07	1.29E+07	7.19E+06	13.15
2-Pentylfuran	15.56	1.12	1.19	3.63	1239	1.92E+07	2.30E+07	5.55E+06	6.55E+06	4.32E+06	2.65E+06	10.21
2-Phenylethanol	33.84	0.54	0.65	5.64	1890	2.25E+07	2.72E+07	9.62E+07	6.61E+07	4.18E+07	4.04E+07	10.28
2-Phenylethyl acetate	31.79	0.59	0.85	4.66	1804	1.82E+06	3.83E+06	2.53E+07	2.11E+07	1.99E+07	8.95E+06	11.07
2-Undecanone	26.41	0.20	1.37	1.69	1609	7.73E+06	8.31E+06	ND	ND	ND	ND	ND
3-Methyl-2(5H)-furanone	29.46	0.48	0.71	5.88	1709	9.36E+05	4.99E+05	1.96E+05	6.25E+05	ND	6.61E+05	7.11
3-Methyl-2-butanol	12.00	1.29	0.71	5.78	1124	1.46E+07	4.46E+07	3.68E+07	3.23E+07	1.93E+07	2.77E+07	22.26
3-Methyl-2-butenal	14.63	1.16	0.86	6.30	1209	3.28E+06	3.81E+06	ND	6.24E+05	ND	1.20E+06	18.73
3-Methylbutanal	6.99	0.81	0.82	5.30	929	1.68E+07	3.35E+06	3.38E+07	3.41E+07	3.67E+07	3.13E+07	6.65
3-Octanol	20.31	0.88	0.91	4.27	1393	8.40E+06	1.05E+07	1.43E+07	1.57E+07	1.47E+07	9.90E+06	8.60
3-Pentanol	11.64	1.29	0.71	5.63	1113	5.01E+06	7.09E+06	1.39E+07	1.82E+07	9.15E+06	5.17E+06	10.64
4-Heptanol	16.99	1.07	0.85	4.27	1284	6.97E+06	8.63E+06	1.02E+07	1.03E+07	6.73E+06	5.88E+06	10.77
4-Hepten-1-ol	23.46	0.77	0.72	5.21	1502	5.36E+06	5.22E+06	7.25E+06	8.09E+06	5.53E+06	4.86E+06	11.30
4-Nonanol	22.84	0.53	0.97	2.03	1481	4.02E+06	8.41E+06	ND	ND	5.78E+06	ND	ND
4-Nonanone	18.47	1.13	1.41	3.59	1333	4.26E+06	3.90E+06	1.23E+07	6.98E+06	ND	3.26E+06	9.24
4-Propylguaiacol	38.23	0.46	0.75	5.58	2087	ND	ND	3.83E+06	4.61E+06	5.15E+06	1.79E+06	27.93
4-Vinylguaiacol	39.96	0.43	0.68	5.95	2168	5.26E+06	ND	7.43E+06	7.91E+06	9.71E+06	3.61E+06	13.92
5,6,7,7a-Tetrahydro-4,4,7a-trimethyl-2(4H)-benzofuranone	43.16	0.38	1.02	4.15	2331	1.09E+07	1.12E+07	1.52E+07	1.44E+07	1.30E+07	5.61E+06	22.17
5,6-Epoxy-β-ionone	35.93	0.34	1.15	3.62	1982	2.31E+07	1.46E+07	ND	4.41E+06	1.10E+07	ND	ND
5-Methyl-3-heptanone	16.26	1.04	1.30	3.05	1261	2.96E+07	3.82E+07	2.08E+07	1.93E+07	4.68E+06	8.97E+06	12.82
6,10,14-Trimethyl-2-pentadecanone	38.70	0.44	1.71	2.99	2110	1.17E+07	2.63E+07	2.68E+07	1.67E+07	1.12E+07	5.72E+06	19.75
6,10-Dimethyl-2-undecanone	28.59	0.64	1.50	3.18	1679	2.19E+06	2.23E+06	3.21E+06	2.28E+06	1.93E+06	7.21E+05	11.15
6-Methyl-2-heptanone	15.87	0.00	1.23	0.00	1248	ND	1.68E+06	ND	ND	ND	ND	ND
6-Methyl-5-hepten-2-ol	22.31	0.82	0.79	4.52	1462	3.50E+07	3.24E+07	1.95E+07	2.03E+07	1.41E+07	1.21E+07	5.13
6-Methyl-5-hepten-2-one	18.84	0.94	1.04	3.83	1345	5.07E+07	4.38E+07	9.19E+06	1.74E+07	1.26E+07	1.58E+07	8.37
6-Undecanol	27.80	0.12	0.81	4.28	1654	7.23E+06	ND	ND	ND	ND	ND	ND
Acetic acid	22.04	1.22	0.47	8.15	1453	5.32E+07	3.92E+07	1.99E+08	2.41E+08	2.46E+08	1.51E+08	7.51
Acetoin	17.32	1.14	0.64	5.74	1295	2.36E+07	1.39E+07	1.12E+07	2.29E+06	5.27E+05	1.03E+06	10.44
Acetone	5.48	0.00	0.58	6.38	822	4.94E+06	1.12E+07	ND	ND	ND	ND	ND
Acetophenone	27.65	0.82	0.79	3.98	1649	ND	1.24E+07	8.70E+06	4.53E+06	ND	7.61E+06	7.77
Acrolein	5.86	0.51	0.58	6.95	834	4.87E+06	2.39E+06	1.40E+06	ND	3.53E+06	2.40E+06	8.34

Amyl acetate	13.80	1.26	1.15	4.10	1182	8.01E+06	1.16E+07	1.56E+07	1.61E+07	1.64E+07	8.36E+06	*4.32*
Benzaldehyde	24.24	0.75	0.75	4.87	1531	7.02E+07	5.48E+07	1.21E+08	1.17E+08	9.39E+07	1.00E+08	*8.07*
Benzyl acetate	29.65	0.60	0.79	4.83	1717	1.32E+06	1.67E+06	2.27E+07	3.47E+07	3.73E+07	1.42E+07	*10.47*
Benzyl alcohol	33.00	0.56	0.60	6.62	1855	1.59E+07	1.95E+07	5.62E+07	7.15E+07	4.46E+07	4.93E+07	*22.09*
Bis(2-hydroxypropyl) ether	31.90	0.74	0.56	3.77	1809	6.49E+05	9.72E+05	1.14E+06	2.20E+06	6.44E+06	5.04E+05	*35.21*
Butanedione	8.37	1.24	0.64	6.45	1013	6.30E+06	8.66E+06	8.23E+06	7.95E+06	ND	1.01E+06	*15.62*
Butyl butyrate	15.34	0.00	1.35	0.00	1232	1.44E+07	8.52E+06	ND	ND	ND	ND	*ND*
Butyl-(Z)-3-hexenoate	21.14	0.90	1.32	3.47	1422	5.46E+05	ND	6.99E+06	6.02E+06	3.91E+06	2.93E+06	*7.93*
Butyric acid	27.39	0.36	0.49	7.32	1641	1.43E+07	1.45E+07	ND	ND	7.50E+06	1.55E+06	*16.33*
cis-Linalool oxide (furanoid)	21.97	0.88	1.02	3.80	1450	1.18E+07	2.10E+07	7.48E+07	8.63E+07	1.85E+07	8.74E+07	*5.38*
Copaene	23.45	0.82	2.07	2.03	1501	5.09E+06	2.52E+06	8.29E+06	8.96E+06	6.56E+06	3.25E+06	*20.94*
Curcumene	30.79	0.61	1.35	3.33	1764	9.33E+05	6.38E+05	2.06E+06	1.95E+06	1.78E+06	9.21E+05	*14.97*
Cyclosativene	23.22	0.84	2.09	2.45	1494	4.60E+06	2.07E+06	8.49E+06	7.45E+06	6.02E+06	2.39E+06	*9.76*
Decanal	23.54	0.76	1.34	3.10	1505	2.44E+07	2.32E+07	1.55E+07	1.34E+07	1.22E+07	6.87E+06	*19.92*
Decanoic acid	41.93	0.39	0.64	6.35	2268	ND	ND	2.69E+06	1.59E+06	1.08E+06	1.78E+06	*11.58*
Diethyl succinate	28.28	0.67	0.87	5.02	1669	ND	ND	3.71E+07	3.14E+07	2.41E+07	1.55E+07	*9.81*
Diethylene glycol monobutyl ether	31.18	0.55	0.77	5.05	1780	2.78E+06	3.77E+06	1.81E+06	9.24E+05	1.49E+06	6.18E+05	*15.27*
Diethylene glycol monoethyl ether	26.86	0.61	0.68	3.65	1623	7.93E+05	1.10E+06	9.03E+05	ND	ND	3.58E+05	*6.24*
Dihydro-β-ionone	32.21	0.55	1.28	3.29	1822	3.63E+06	2.03E+06	6.36E+06	6.93E+06	4.24E+06	3.71E+06	*11.69*
Dimethyl Sulfoxide	24.97	0.00	0.70	1.42	1557	ND	2.41E+06	ND	ND	ND	ND	*ND*
Dimethylmaleic anhydride	30.02	0.33	0.80	5.50	1732	5.87E+05	7.43E+05	ND	ND	ND	1.01E+06	*31.65*
Dodecanal	29.17	0.63	1.42	3.16	1698	2.46E+06	2.36E+06	3.36E+06	3.02E+06	2.41E+06	1.95E+06	*17.45*
Dodecane	14.38	1.12	3.06	2.09	1200	3.04E+06	5.74E+06	1.81E+07	1.90E+07	1.89E+07	1.34E+07	*10.24*
Ethanol	7.213	0.13	2.33	1.24	948	2.06E+07	2.10E+07	9.88E+07	1.04E+08	6.53E+07	5.82E+07	*12.35*
Ethyl (4E)-4-heptenoate	20.22	1.13	1.19	3.83	1390	6.27E+06	3.85E+07	3.96E+07	3.34E+07	1.42E+06	3.36E+07	*27.71*
Ethyl 2-hexenoate	19.10	1.01	1.14	3.59	1353	5.32E+07	3.10E+07	5.19E+06	2.05E+07	9.93E+06	3.19E+07	*2.76*
Ethyl 2-hydroxy-3-methyl butyrate	21.51	0.94	0.80	1.13	1434	ND	ND	4.88E+06	ND	ND	4.99E+05	*13.24*
Ethyl 2-hydroxy-3-phenylpropanoate	41.75	0.40	0.75	5.43	2259	ND	ND	4.08E+07	2.29E+07	1.03E+07	1.29E+07	*15.49*
Ethyl 2-methylbutanoate	10.22	1.24	1.22	3.33	1069	4.42E+06	5.42E+06	4.03E+06	4.46E+06	4.23E+06	3.46E+06	*11.68*
Ethyl 3-phenylpropanoate	33.40	0.59	0.91	4.68	1872	1.63E+06	ND	1.62E+07	1.34E+07	1.60E+07	5.38E+06	*9.59*
Ethyl 4-octenoate	22.99	0.77	1.24	4.03	1485	6.91E+05	ND	4.70E+06	5.50E+06	ND	4.07E+06	*2.96*
Ethyl 9-hexadecenoate	41.94	0.45	1.56	3.54	2269	4.44E+06	1.14E+06	6.74E+06	5.60E+06	3.72E+06	2.08E+06	*23.49*
Ethyl acetate	6.48	0.84	0.71	5.63	854	4.85E+07	4.13E+07	8.40E+07	9.62E+07	7.40E+07	7.25E+07	*6.87*
Ethyl benzoate	28.12	0.65	0.88	4.41	1664	9.42E+06	7.28E+06	3.19E+07	3.11E+07	2.87E+07	1.79E+07	*10.37*
Ethyl caprate	27.29	0.64	1.49	4.33	1637	9.71E+05	ND	2.13E+07	1.96E+07	1.75E+07	1.02E+07	*9.89*
Ethyl dodecanoate	32.44	0.57	1.57	3.15	1832	3.59E+06	1.73E+06	3.38E+07	3.00E+07	2.58E+07	1.59E+07	*12.74*
Ethyl heptanoate	18.70	0.96	1.36	3.17	1340	7.15E+06	ND	3.38E+07	3.38E+07	1.42E+07	1.63E+07	*1.33*
Ethyl hexanoate	15.67	1.09	1.30	3.13	1242	4.47E+07	3.21E+07	1.08E+08	1.04E+08	8.18E+07	8.01E+07	*10.88*
Ethyl isovalerate	10.67	1.21	1.18	3.34	1083	5.54E+06	8.47E+06	1.05E+07	1.15E+07	6.38E+06	6.26E+06	*4.16*
Ethyl lactate	46.52	0.33	1.51	3.81	>2500	ND	ND	1.89E+07	1.59E+07	7.92E+06	1.38E+07	*0.32*
Ethyl linoleate	47.70	0.31	1.39	3.76	>2500	1.54E+06	ND	6.19E+07	4.42E+07	2.63E+07	1.26E+07	*19.14*
Ethyl linolenate	18.96	0.98	0.67	5.65	1349	ND	ND	3.42E+07	2.46E+07	1.29E+07	1.04E+07	*15.55*
Ethyl myristate	37.08	0.47	1.63	2.96	2034	3.09E+06	1.83E+06	3.73E+07	2.78E+07	2.14E+07	1.48E+07	*19.59*
Ethyl nonanoate	24.56	0.74	1.46	2.99	1542	1.42E+06	9.36E+05	1.87E+07	1.52E+07	2.47E+08	1.33E+07	*20.60*
Ethyl octanoate	21.72	0.84	1.42	3.08	1442	4.48E+06	ND	5.08E+07	4.51E+07	3.58E+07	2.71E+07	*11.38*
Ethyl oleate	45.69	0.35	1.62	3.05	2466	7.48E+06	6.13E+06	3.26E+07	1.99E+07	1.30E+07	3.96E+06	*11.33*
Ethyl palmitate	41.36	0.39	1.69	3.15	2240	1.76E+07	1.19E+07	1.19E+08	9.73E+07	8.59E+07	6.53E+07	*15.90*
Ethyl phenylacetate	30.99	0.59	0.86	5.04	1772	ND	ND	2.92E+07	2.74E+07	1.67E+07	1.40E+07	*13.40*
Ethyl propionate	7.87	1.07	0.87	4.24	993	8.19E+06	7.64E+06	1.01E+07	2.44E+07	9.63E+07	7.47E+06	*5.33*
Ethyl sorbate	23.76	0.75	0.95	4.51	1513	ND	ND	3.08E+07	3.20E+07	2.35E+07	1.83E+07	*9.91*
Ethyl valerate	12.63	1.22	1.19	3.55	1145	1.86E+07	1.60E+07	2.15E+07	2.13E+07	1.27E+07	1.58E+07	*14.31*
Ethylbenzene	12.39	0.53	1.05	2.69	1137	8.77E+06	9.70E+06	ND	ND	ND	8.75E+05	*15.33*
Furfural	21.50	0.85	0.63	5.71	1434	ND	ND	1.74E+06	1.13E+06	8.32E+05	1.96E+06	*7.92*
Furfuryl alcohol	27.68	0.76	0.55	6.78	1650	5.02E+05	7.19E+05	2.81E+07	1.89E+07	8.80E+06	1.02E+07	*9.30*
Geranial	29.81	0.50	1.07	4.14	1724	2.58E+06	1.32E+06	1.24E+06	ND	ND	3.20E+06	*32.13*
Geraniol	32.30	0.58	0.79	4.60	1826	2.55E+06	2.22E+06	5.33E+06	6.11E+06	4.22E+06	3.41E+06	*12.86*
Geranylacetone	32.67	0.56	1.20	3.59	1841	3.54E+07	3.36E+07	4.51E+07	5.34E+07	2.65E+07	3.24E+07	*18.22*
Guaiacol	32.68	0.63	0.63	6.80	1842	1.51E+07	ND	5.00E+07	5.06E+07	2.47E+07	8.51E+06	*9.44*

Heptanal	14.12	1.27	1.17	3.60	1193	1.92E+07	1.16E+07	4.27E+06	8.78E+06	2.16E+06	5.59E+06	*16.00*
Heptane	4.49	0.00	0.86	4.36	700	1.29E+06	2.71E+06	ND	ND	ND	4.37E+05	*25.03*
Heptanoic acid	35.21	0.47	0.56	7.03	1950	3.53E+06	3.46E+06	6.25E+06	8.48E+06	4.04E+06	5.71E+06	*36.81*
Heptyl acetate	19.92	0.98	1.28	3.71	1381	5.26E+06	5.49E+06	8.12E+06	1.55E+07	1.62E+07	5.63E+06	*11.20*
Hexadecane	26.13	0.64	2.88	1.98	1600.00	2.39E+06	2.07E+06	3.35E+06	4.49E+06	3.02E+06	2.77E+06	*16.33*
Hexadecanolide	43.85	0.44	1.58	3.16	2368	ND	ND	6.05E+05	8.33E+05	3.27E+06	1.54E+05	*8.89*
Hexanal	11.02	1.20	1.04	3.97	1093	7.43E+07	8.77E+07	1.93E+07	1.33E+07	1.10E+07	1.24E+07	*18.63*
Hexanenitrile	17.69	1.02	0.93	4.27	1307	5.61E+06	4.85E+06	2.48E+06	3.29E+06	2.13E+06	2.64E+06	*6.23*
Hexanoic acid	32.66	0.53	0.54	7.29	1841	3.05E+07	4.20E+07	2.35E+07	3.06E+07	4.17E+07	3.59E+07	*12.67*
Hexyl acetate	16.88	1.05	1.22	2.98	1281	3.91E+07	4.89E+07	7.76E+07	8.64E+07	4.90E+07	5.53E+07	*9.98*
Hexyl hexanoate	26.61	0.69	1.59	2.74	1615	1.37E+06	1.26E+06	3.76E+06	5.83E+06	3.70E+06	2.53E+06	*10.92*
Hotrienol	26.46	0.70	0.81	5.54	1611	ND	ND	ND	6.81E+06	2.25E+06	5.82E+06	*3.52*
Isoamyl acetate	12.25	1.26	1.14	3.79	1133	1.30E+07	2.17E+07	8.93E+07	9.07E+07	4.74E+07	4.72E+07	*11.74*
Isoamyl alcohol	14.64	1.21	0.69	6.19	1209	2.86E+07	4.85E+07	8.15E+07	6.84E+07	4.97E+07	5.95E+07	*24.55*
Isoamyl butyrate	16.69	0.84	1.48	2.56	1275	1.04E+06	3.39E+06	1.79E+06	ND	3.41E+06	3.34E+05	*12.33*
Isoamyl hexanoate	22.43	0.79	1.59	2.78	1466	1.28E+07	6.15E+06	4.21E+07	3.98E+07	2.57E+07	1.85E+07	*8.53*
Isoamyl lactate	25.47	0.72	0.81	4.98	1576	ND	ND	4.18E+07	2.63E+07	7.59E+06	1.73E+07	*8.18*
Isoamyl propionate	14.29	1.17	1.34	3.60	1198	5.56E+05	1.87E+06	4.46E+06	2.68E+07	5.24E+07	4.57E+06	*8.67*
Isobutanol	11.21	1.27	0.63	5.77	1099	7.64E+06	5.15E+06	1.65E+07	1.70E+07	5.15E+06	7.29E+06	*7.99*
Isobutyl hexanoate	19.30	0.93	1.58	2.98	1360	6.84E+05	6.91E+05	6.02E+06	6.04E+06	2.63E+06	2.39E+06	*10.46*
Isobutyraldehyde	5.44	0.48	0.66	5.15	821	3.55E+06	ND	3.67E+06	3.00E+06	6.30E+06	4.69E+06	*4.28*
Isobutyric acid	25.66	0.60	0.53	9.96	1583	1.22E+06	3.85E+06	9.23E+06	ND	3.63E+06	ND	*ND*
Isoeugenol	42.90	0.37	0.71	6.73	2317	ND	ND	2.29E+06	1.63E+06	1.76E+06	8.73E+05	*18.88*
Isopropyl myristate	36.87	0.47	1.77	2.49	2025	9.49E+05	1.41E+06	5.45E+05	4.01E+05	5.94E+05	3.27E+05	*12.37*
Isovaleric acid	28.38	0.64	0.52	7.46	1672	2.28E+07	1.84E+07	4.03E+07	4.15E+07	9.34E+06	9.58E+06	*17.13*
Ketoisophorone	28.95	0.09	0.89	2.60	1691	5.54E+06	5.96E+06	ND	ND	ND	ND	*ND*
Lauric acid	45.92	0.57	0.70	6.46	2478	2.05E+06	2.34E+06	4.56E+06	2.72E+06	1.23E+06	1.31E+06	*32.75*
Limonene	14.48	1.12	1.51	3.04	1204	1.43E+07	1.75E+07	2.81E+07	1.53E+07	2.07E+07	1.27E+07	*10.97*
Linalool	24.73	0.76	0.87	4.09	1549	5.39E+06	6.28E+06	9.25E+07	8.65E+07	5.28E+07	4.82E+07	*22.38*
Menthol	27.26	0.67	0.93	4.22	1636	7.33E+06	9.09E+06	6.77E+06	5.98E+06	7.06E+06	1.52E+06	*4.27*
Methyl (E)-2-hexenoate	17.60	0.17	1.05	0.98	1304	2.66E+06	3.26E+06	ND	ND	ND	ND	*ND*
Methyl (Z)-3-hexenoate	16.49	1.08	0.98	4.11	1268	1.85E+07	4.42E+06	1.50E+07	1.10E+07	6.48E+06	1.16E+07	*10.38*
Methyl acetate	5.64	0.68	0.60	6.24	827	1.99E+07	1.91E+07	4.81E+06	1.36E+07	1.89E+07	6.48E+06	*9.99*
Methyl benzoate	26.98	0.64	0.81	5.00	1627	5.60E+06	2.12E+06	4.81E+06	4.62E+06	3.31E+06	4.68E+06	*8.37*
Methyl butyrate	8.59	0.31	0.86	4.31	1019	2.88E+07	2.29E+07	ND	ND	ND	ND	*ND*
Methyl ethyl ketone	6.71	0.00	0.72	4.94	909	1.70E+06	1.67E+07	ND	ND	ND	ND	*ND*
Methyl hexanoate	14.23	1.21	1.13	3.96	1196	1.71E+07	3.49E+06	7.40E+06	9.28E+06	8.78E+06	9.36E+06	*27.44*
Methyl isovalerate	9.45	0.00	0.98	2.82	1045	3.73E+06	3.77E+06	ND	ND	ND	ND	*ND*
Methyl palmitate	40.61	0.42	1.56	3.01	2204	8.71E+06	5.78E+06	9.02E+06	9.25E+06	8.93E+06	2.23E+06	*16.96*
Methyl pentanoate	11.14	1.05	1.02	2.80	1097	ND	7.14E+06	9.06E+05	2.03E+06	1.21E+06	ND	*ND*
Methyl salicylate	30.91	0.58	0.81	5.01	1768	4.58E+06	1.13E+06	1.60E+07	1.80E+07	1.97E+07	1.12E+07	*20.32*
m-Xylene	12.69	1.23	1.04	3.65	1147	1.18E+07	1.26E+07	4.92E+06	4.89E+06	3.21E+06	2.90E+06	*12.47*
Naphthalene	30.09	0.17	0.85	2.73	1735	2.37E+06	2.70E+06	ND	ND	ND	ND	*ND*
Neophytadiene	34.25	0.52	2.26	2.44	1908	1.84E+07	1.44E+07	1.92E+07	2.11E+07	1.74E+07	1.35E+07	*13.96*
Neral	28.69	0.60	1.07	3.35	1683	3.46E+06	2.55E+06	ND	ND	ND	1.61E+05	*13.61*
Nerol oxide	22.65	0.82	1.14	3.67	1474	ND	ND	2.92E+06	8.70E+06	6.55E+06	4.75E+06	*11.30*
Nonanal	20.51	0.92	1.29	3.12	1400	2.24E+07	1.87E+07	2.69E+07	2.65E+07	2.21E+07	1.72E+07	*14.02*
Nonane	6.59	0.75	1.90	3.14	900	1.78E+06	4.98E+06	1.08E+07	7.75E+06	5.10E+06	1.05E+07	*12.33*
Nonanoic acid	39.86	0.40	0.61	6.30	2163	5.10E+06	1.07E+07	1.07E+07	1.10E+07	7.63E+06	1.30E+07	*10.51*
Nonyl acetate	25.70	0.68	1.38	3.12	1584	ND	1.11E+06	9.31E+06	1.81E+07	1.27E+07	3.95E+06	*8.21*
Octanal	17.35	1.09	1.25	3.10	1296	1.99E+07	6.45E+06	1.14E+07	1.19E+07	5.43E+06	8.93E+06	*20.43*
Octane	5.25	0.22	1.30	3.63	800	1.40E+06	2.22E+06	8.11E+05	1.14E+06	4.74E+05	4.51E+05	*16.48*
Octanoic acid	37.76	0.07	0.60	3.85	2066	4.59E+06	4.96E+06	ND	ND	ND	ND	*ND*
Octyl acetate	22.90	0.75	1.33	3.07	1483	3.80E+06	3.76E+06	3.22E+07	2.46E+07	2.64E+07	7.03E+06	*7.97*
o-Xylene	14.05	1.09	1.03	3.87	1190	5.34E+06	5.04E+06	2.47E+06	3.92E+06	1.88E+06	2.48E+06	*6.61*
p-Cresol	37.49	0.45	0.57	6.71	2053	ND	ND	2.25E+07	2.77E+07	2.61E+07	1.79E+07	*31.32*
p-Cymene	16.78	0.91	1.25	1.54	1277	2.58E+06	3.37E+06	4.77E+06	ND	1.90E+06	4.96E+05	*13.40*
Pentadecanal	36.78	0.15	1.54	1.54	2020	1.33E+07	7.57E+06	ND	ND	ND	ND	*ND*

Pentadecane	23.41	0.82	2.97	2.47	1500.00	1.01E+07	2.53E+06	1.28E+07	1.15E+07	1.12E+07	1.68E+07	*5.24*
Pentanoic acid	30.07	0.64	0.53	7.37	1734	ND	3.11E+06	8.05E+06	1.19E+07	ND	5.41E+06	*9.55*
Phenol	35.88	0.51	0.54	7.72	1980	1.85E+07	1.07E+07	2.45E+07	5.45E+07	4.80E+07	1.75E+07	*4.40*
Phenoxyethanol	38.85	0.44	0.64	6.54	2116	4.91E+06	5.44E+06	1.02E+07	2.17E+07	1.51E+07	6.58E+06	*24.16*
Phenylacetaldehyde	27.43	0.67	0.76	4.83	1642	1.49E+07	1.36E+07	4.61E+07	4.39E+07	4.78E+07	3.54E+07	*9.03*
Propane	4.49	2.45	0.50	6.96	NC	1.00E+07	4.73E+06	2.84E+06	3.33E+06	3.54E+06	5.25E+06	*24.17*
Propionic acid	24.73	1.04	0.49	7.54	1548	6.13E+06	4.72E+06	1.58E+07	7.35E+07	2.48E+08	7.65E+06	*13.32*
Propyl acetate	8.23	1.23	0.88	5.22	1008	ND	ND	ND	1.04E+08	1.94E+08	1.69E+07	*6.80*
Propyl butyrate	12.32	0.66	1.22	3.65	1135	2.05E+07	1.62E+07	ND	ND	3.19E+07	ND	*ND*
Propyl decanoate	29.44	0.28	1.59	2.73	1709	ND	ND	ND	ND	2.26E+07	ND	*ND*
Propyl hexanoate	18.28	0.97	1.42	2.96	1326	1.02E+07	7.44E+06	ND	5.80E+07	1.40E+08	1.82E+07	*12.47*
Propyl isovalerate	13.22	1.00	1.37	2.75	1164	1.91E+06	3.28E+06	ND	7.90E+06	1.65E+07	ND	*ND*
Propyl laurate	34.33	0.36	1.64	2.39	1911	ND	ND	ND	3.60E+06	2.26E+07	ND	*ND*
Propyl palmitate	43.04	0.37	1.76	2.81	2324	6.50E+06	2.92E+06	6.20E+06	1.67E+07	6.78E+07	3.89E+05	*22.88*
Propyl pentanoate	15.24	1.13	1.36	3.64	1228	ND	ND	ND	1.05E+07	3.46E+07	1.00E+06	*17.07*
Propyl phenylacetate	32.91	0.25	0.90	3.82	1852	ND	ND	ND	ND	2.89E+07	ND	*ND*
Propyl propionate	10.01	1.25	1.08	3.64	1062	5.48E+06	2.53E+06	7.44E+06	1.49E+07	1.44E+08	4.85E+06	*5.31*
Propylbenzene	14.92	0.87	1.16	3.80	1218	1.42E+06	1.72E+06	3.60E+05	2.93E+05	1.62E+05	2.19E+05	*12.33*
p-Xylene	12.52	1.15	1.06	4.16	1141	9.14E+06	9.71E+06	4.05E+06	2.66E+06	2.67E+06	2.19E+06	*3.61*
Styrene	16.30	1.09	0.88	4.34	1262	7.71E+06	6.77E+06	1.20E+07	1.08E+07	6.24E+06	5.51E+06	*7.75*
Tetradecanal	34.25	0.52	1.49	2.94	1908	1.86E+06	1.94E+06	2.14E+06	1.34E+06	1.17E+06	1.16E+06	*3.33*
Tetradecane	20.51	0.86	2.99	1.46	1400	8.17E+06	8.23E+06	1.55E+07	1.24E+07	1.20E+07	1.51E+07	*9.37*
Theaspirane	24.75	0.77	1.57	3.18	1549	ND	ND	4.73E+07	7.00E+07	1.51E+07	2.07E+07	*26.06*
Toluene	9.81	1.14	0.90	4.86	1056	9.13E+06	9.62E+06	8.09E+06	4.97E+06	5.88E+06	5.21E+07	*9.33*
trans-4,5-Epoxy-(E)-2-decenal	35.95	0.88	0.89	6.36	1983	4.90E+06	2.06E+06	6.21E+06	3.18E+06	1.15E+06	ND	*ND*
trans-Linalool oxide (furanoid isomer)	22.74	0.83	1.00	4.48	1477	3.99E+06	ND	2.66E+07	2.00E+07	9.70E+06	1.05E+07	*4.71*
Tridecanal	31.78	0.64	1.46	3.13	1804	1.72E+06	1.47E+06	3.26E+06	1.31E+06	2.74E+06	1.14E+06	*20.16*
Tridecane	17.48	1.00	3.05	2.17	1300	7.72E+06	7.27E+06	1.05E+07	9.87E+06	9.97E+06	1.41E+07	*11.33*
Undecane	11.24	1.63	2.98	2.69	1100	2.06E+06	2.67E+06	7.44E+06	3.12E+06	4.70E+06	1.08E+07	*1.37*
Untargeted (100)	15.38	1.11	0.99	4.11	1233	1.06E+07	5.63E+06	7.63E+06	1.09E+07	1.13E+07	5.17E+06	*7.94*
Untargeted (104)	15.75	0.00	2.85	1.76	1245	ND	ND	ND	ND	ND	8.32E+05	*17.65*
Untargeted (107)	15.88	1.24	1.30	3.23	1249	ND	ND	1.08E+07	1.29E+07	ND	8.42E+06	*10.85*
Untargeted (108)	15.91	1.08	1.10	3.33	1250	1.55E+06	4.62E+06	2.90E+06	2.68E+06	1.83E+06	1.45E+06	*16.86*
Untargeted (110)	15.99	1.26	2.72	2.19	1252	1.13E+06	3.78E+06	4.30E+06	2.06E+06	2.26E+06	6.83E+05	*7.97*
Untargeted (112)	16.12	0.19	1.32	2.81	1256	7.61E+06	3.74E+06	ND	ND	ND	ND	*ND*
Untargeted (121)	16.98	0.26	0.92	3.00	1284	3.45E+06	1.54E+06	ND	ND	ND	ND	*ND*
Untargeted (124)	17.14	1.06	2.57	2.34	1289	9.12E+05	1.09E+06	1.55E+06	1.19E+06	8.26E+05	4.85E+05	*2.17*
Untargeted (128)	17.44	0.00	2.90	2.10	1299	ND	ND	ND	ND	ND	2.27E+06	*13.84*
Untargeted (129)	17.44	1.13	1.11	3.71	1299	ND	ND	1.83E+07	1.35E+07	2.48E+07	6.85E+06	*2.47*
Untargeted (133)	17.76	1.06	1.10	3.39	1309	3.46E+07	1.97E+07	1.36E+08	1.30E+08	8.20E+07	9.61E+07	*7.21*
Untargeted (135)	17.91	0.38	1.23	1.86	1314	5.29E+06	4.32E+06	ND	ND	ND	ND	*ND*
Untargeted (14)	7.40	4.10	0.68	18.01	959	4.95E+06	5.10E+06	3.72E+07	1.41E+07	3.87E+07	ND	*ND*
Untargeted (146)	18.69	35.00	2.20	2.31	1340	8.76E+05	1.49E+06	1.08E+07	6.86E+06	7.97E+06	8.94E+06	*16.85*
Untargeted (15)	7.41	0.00	1.46	2.16	960	1.79E+06	1.28E+06	ND	ND	ND	ND	*ND*
Untargeted (150)	18.85	0.99	0.77	4.84	1345	1.13E+07	9.56E+06	8.33E+06	8.69E+06	5.80E+06	6.00E+06	*9.39*
Untargeted (152)	19.00	0.93	1.62	3.23	1350	ND	ND	4.21E+07	3.87E+07	1.65E+07	4.81E+07	*14.79*
Untargeted (156)	19.47	0.47	1.24	3.74	1366	3.85E+06	4.11E+06	ND	ND	5.06E+05	ND	*ND*
Untargeted (161)	20.24	0.96	1.21	3.35	1391	ND	3.72E+06	1.22E+07	2.71E+07	1.32E+08	1.12E+07	*10.26*
Untargeted (163)	20.42	0.00	1.17	0.00	1397	ND	1.43E+06	ND	ND	ND	ND	*ND*
Untargeted (168)	20.71	0.90	2.87	2.81	1407	1.35E+06	ND	3.38E+05	1.49E+06	1.42E+06	9.96E+05	*7.33*
Untargeted (169)	20.72	0.89	1.06	4.01	1407	ND	ND	4.17E+06	6.40E+06	3.33E+06	3.78E+06	*7.06*
Untargeted (17)	7.66	2.50	1.07	4.48	978	2.72E+06	2.97E+06	6.35E+06	1.06E+07	1.81E+07	5.40E+06	*8.82*
Untargeted (170)	20.73	0.14	0.79	5.31	1407	5.56E+06	1.89E+06	ND	ND	7.60E+05	ND	*ND*
Untargeted (174)	20.83	0.86	1.13	3.34	1411	1.84E+07	9.50E+06	1.47E+07	9.63E+06	5.98E+06	9.37E+06	*28.79*
Untargeted (177)	20.94	1.00	0.84	4.41	1415	1.68E+06	3.23E+06	5.93E+06	5.24E+06	3.80E+06	2.95E+06	*10.63*
Untargeted (178)	21.06	0.00	1.09	0.00	1419	3.12E+06	ND	ND	ND	ND	ND	*ND*
Untargeted (180)	21.13	0.84	2.62	2.86	1422	2.67E+06	2.66E+06	2.93E+06	2.08E+06	1.49E+06	2.59E+05	*7.33*
Untargeted (182)	21.14	0.94	1.14	3.78	1422	3.21E+06	ND	1.28E+07	1.01E+07	9.68E+06	4.31E+06	*10.74*

Untargeted (183)	21.22	0.85	1.04	3.58	1424	1.75E+07	1.12E+07	5.68E+06	9.17E+06	6.53E+06	4.87E+06	*8.68*
Untargeted (184)	21.35	0.00	1.15	0.00	1429	ND	2.08E+06	ND	ND	ND	ND	*ND*
Untargeted (185)	21.38	0.90	1.28	3.24	1430	ND	ND	6.41E+06	6.56E+06	2.12E+06	3.33E+06	*18.37*
Untargeted (186)	21.44	0.83	0.72	5.54	1432	ND	ND	3.88E+06	2.43E+07	4.25E+07	5.07E+06	*23.63*
Untargeted (187)	21.45	0.95	1.45	3.36	1432	ND	ND	6.36E+06	1.36E+07	ND	3.76E+06	*9.89*
Untargeted (193)	21.92	1.34	0.78	3.17	1449	2.63E+06	3.18E+06	7.60E+06	6.65E+06	2.98E+07	4.69E+06	*8.50*
Untargeted (196)	22.04	2.26	0.55	8.15	1453	2.79E+06	ND	ND	ND	ND	ND	*ND*
Untargeted (197)	22.05	0.49	1.13	8.48	1453	4.79E+06	8.63E+05	ND	ND	ND	ND	*ND*
Untargeted (198)	22.07	0.83	1.43	3.07	1454	ND	ND	2.14E+07	1.88E+07	8.52E+06	7.91E+06	*17.20*
Untargeted (199)	22.11	0.87	1.35	3.80	1455	9.52E+05	ND	ND	1.31E+07	1.72E+07	1.63E+07	*3.05*
Untargeted (200)	22.12	0.87	0.99	4.13	1456	ND	8.98E+05	1.30E+07	1.28E+07	9.68E+07	1.21E+07	*6.60*
Untargeted (203)	22.43	0.86	1.38	3.00	1466	ND	ND	1.71E+07	2.37E+07	3.75E+06	9.19E+06	*26.51*
Untargeted (204)	22.47	0.86	0.64	5.65	1468	ND	ND	2.23E+07	1.41E+07	1.38E+07	1.13E+07	*5.00*
Untargeted (207)	22.55	0.74	3.13	1.59	1471	2.26E+07	1.96E+07	3.60E+07	3.94E+07	5.29E+07	ND	*ND*
Untargeted (215)	23.00	0.13	0.68	5.42	1486	1.84E+06	1.08E+06	ND	ND	ND	ND	*ND*
Untargeted (216)	23.02	0.78	0.74	5.13	1487	1.22E+07	2.11E+07	5.18E+06	1.50E+07	1.05E+07	8.07E+06	*13.68*
Untargeted (217)	23.04	0.80	1.46	3.02	1487	ND	ND	2.86E+07	2.83E+07	1.93E+07	1.07E+07	*6.63*
Untargeted (22)	7.95	0.72	2.26	2.66	1000	9.77E+05	1.09E+06	ND	ND	ND	2.52E+05	*5.70*
Untargeted (222)	23.52	0.78	1.18	3.28	1504	ND	ND	8.46E+06	9.09E+06	7.84E+06	4.24E+06	*4.93*
Untargeted (224)	23.60	0.77	0.66	5.87	1507	1.04E+06	ND	9.10E+06	7.26E+06	5.51E+06	4.02E+06	*4.23*
Untargeted (226)	23.66	0.84	0.81	3.83	1509	1.79E+06	ND	ND	6.92E+06	ND	2.24E+06	*16.33*
Untargeted (23)	7.97	2.99	1.03	3.52	1000	5.00E+06	4.85E+06	9.98E+06	1.15E+07	9.95E+06	3.61E+06	*7.90*
Untargeted (231)	23.99	0.76	0.86	4.55	1522	2.30E+06	1.55E+06	6.98E+06	9.37E+06	5.44E+06	2.87E+06	*9.57*
Untargeted (232)	24.18	0.76	1.36	3.34	1528	ND	ND	1.81E+07	1.12E+07	9.28E+06	9.74E+06	*6.68*
Untargeted (236)	24.27	0.75	1.30	3.66	1532	ND	ND	8.88E+06	7.09E+06	7.33E+06	7.53E+06	*3.01*
Untargeted (237)	24.45	0.78	0.57	6.55	1538	2.82E+06	2.93E+06	9.99E+06	7.37E+06	ND	6.22E+06	*0.51*
Untargeted (238)	24.50	0.00	0.93	0.00	1540	ND	2.45E+06	ND	ND	ND	ND	*ND*
Untargeted (244)	24.80	0.85	2.46	2.69	1551	2.17E+06	1.92E+06	1.15E+06	7.41E+06	ND	3.47E+06	*16.91*
Untargeted (245)	24.96	0.55	0.95	2.57	1557	2.10E+07	8.28E+06	ND	7.57E+05	ND	ND	*ND*
Untargeted (246)	24.97	0.00	0.78	0.00	1557	1.93E+06	ND	ND	ND	ND	ND	*ND*
Untargeted (249)	24.99	0.41	1.15	3.39	1558	1.33E+07	9.69E+06	ND	ND	ND	1.78E+07	*7.35*
Untargeted (250)	25.36	0.20	0.84	3.12	1572	1.96E+06	1.41E+06	ND	ND	ND	ND	*ND*
Untargeted (251)	25.36	0.50	0.93	3.69	1572	1.01E+07	5.42E+06	ND	2.11E+05	ND	ND	*ND*
Untargeted (257)	25.74	0.70	0.80	4.38	1586	7.43E+06	9.15E+06	1.04E+07	2.23E+07	1.17E+07	5.54E+06	*10.74*
Untargeted (258)	25.86	0.81	0.53	5.94	1590	9.77E+06	3.25E+06	1.79E+07	1.14E+08	3.93E+07	2.98E+07	*8.33*
Untargeted (259)	25.87	0.70	1.29	3.44	1590	ND	ND	1.61E+07	2.04E+07	1.34E+07	6.94E+06	*9.24*
Untargeted (261)	26.02	0.69	0.93	3.78	1596	7.27E+06	6.41E+06	1.13E+07	1.32E+07	6.23E+06	5.26E+06	*8.81*
Untargeted (264)	26.24	0.72	1.39	3.39	1603	ND	ND	8.59E+06	1.21E+07	1.16E+07	5.46E+06	*7.91*
Untargeted (267)	26.45	0.70	0.91	4.25	1610	2.24E+07	2.43E+07	1.18E+07	1.42E+07	5.50E+06	5.96E+06	*5.08*
Untargeted (27)	8.56	2.36	0.97	4.52	1018	6.60E+06	7.69E+06	1.74E+07	1.39E+07	2.99E+06	8.29E+06	*6.10*
Untargeted (274)	26.56	0.69	1.30	3.30	1614	4.21E+06	3.90E+06	7.37E+06	8.91E+06	1.03E+07	ND	*ND*
Untargeted (274)	26.86	0.58	3.14	2.05	1623	ND	ND	1.14E+07	1.53E+07	1.41E+07	8.32E+06	*10.88*
Untargeted (278)	27.09	0.11	0.95	3.23	1631	6.57E+05	9.93E+05	ND	ND	ND	ND	*ND*
Untargeted (280)	27.18	0.49	0.50	5.99	1634	ND	ND	5.37E+06	9.43E+06	5.48E+06	ND	*ND*
Untargeted (284)	27.42	0.00	1.52	4.21	1641	ND	ND	ND	ND	ND	5.65E+05	*14.20*
Untargeted (285)	27.42	0.53	0.57	6.91	1641	ND	ND	ND	ND	1.98E+07	ND	*ND*
Untargeted (288)	27.46	0.25	0.82	2.79	1643	4.34E+05	7.25E+05	ND	ND	ND	ND	*ND*
Untargeted (289)	27.51	0.65	1.02	4.63	1645	ND	ND	2.72E+07	2.23E+07	1.49E+07	1.18E+07	*18.15*
Untargeted (293)	27.69	0.19	1.03	2.52	1650	1.91E+06	2.07E+06	6.97E+05	ND	ND	ND	*ND*
Untargeted (294)	27.77	0.00	1.48	4.09	1653	ND	ND	ND	ND	ND	1.63E+06	*12.93*
Untargeted (297)	27.88	0.68	0.74	4.59	1656	5.64E+06	1.03E+07	1.31E+07	1.29E+07	6.60E+06	7.86E+06	*9.23*
Untargeted (298)	28.03	0.33	0.63	7.28	1661	3.23E+06	1.77E+06	ND	ND	1.94E+06	ND	*ND*
Untargeted (299)	28.04	0.18	1.00	3.18	1662	3.89E+06	2.73E+06	ND	ND	ND	ND	*ND*
Untargeted (300)	28.05	0.63	1.62	3.88	1662	2.28E+06	8.97E+05	1.31E+06	2.32E+06	2.54E+06	9.32E+05	*13.47*
Untargeted (302)	28.28	0.46	0.55	2.25	1669	ND	ND	ND	ND	2.10E+07	ND	*ND*
Untargeted (304)	28.35	0.00	0.66	0.00	1672	ND	6.22E+05	ND	ND	ND	ND	*ND*
Untargeted (306)	28.41	0.88	1.66	3.61	1674	2.94E+06	3.08E+06	4.06E+06	6.51E+06	4.99E+06	1.95E+06	*10.05*
Untargeted (31)	9.02	1.16	1.08	3.93	1032	3.08E+06	3.42E+06	3.13E+06	3.46E+06	1.43E+06	1.69E+06	*12.18*

Untargeted (311)	28.77	0.70	0.79	4.74	1685	3.87E+06	1.44E+06	2.61E+06	2.83E+06	9.34E+05	2.23E+06	*19.15*
Untargeted (314)	29.00	0.22	1.65	5.01	1693	9.55E+05	ND	ND	ND	ND	8.52E+05	*8.08*
Untargeted (316)	29.08	0.61	0.92	4.41	1695	ND	ND	5.55E+06	7.46E+06	6.52E+06	5.60E+06	*15.23*
Untargeted (319)	29.37	0.11	0.77	4.50	1706	1.53E+06	ND	ND	ND	ND	ND	*ND*
Untargeted (320)	29.39	0.92	1.04	4.07	1707	1.10E+06	9.59E+05	5.84E+06	4.87E+06	6.72E+06	1.86E+06	*19.80*
Untargeted (324)	29.64	0.51	0.60	5.30	1717	ND	ND	4.44E+06	3.22E+06	2.67E+07	3.19E+05	*11.37*
Untargeted (327)	29.81	0.00	0.91	0.00	1724	4.20E+05	ND	ND	ND	ND	ND	*ND*
Untargeted (33)	9.31	1.13	1.72	3.11	1041	6.51E+06	8.35E+06	7.31E+06	4.67E+06	7.18E+06	3.78E+06	*7.43*
Untargeted (335)	30.28	0.50	0.52	6.30	1743	8.00E+06	6.04E+06	ND	ND	5.68E+06	ND	*ND*
Untargeted (336)	30.32	0.41	0.92	5.33	1745	ND	ND	ND	1.85E+06	2.96E+07	ND	*ND*
Untargeted (338)	30.33	0.40	0.63	4.74	1745	5.13E+06	3.83E+06	ND	7.40E+05	6.84E+06	ND	*ND*
Untargeted (339)	30.54	0.18	0.75	3.09	1753	3.64E+06	3.01E+06	ND	ND	ND	ND	*ND*
Untargeted (344)	31.31	0.17	0.94	3.35	1785	6.59E+06	5.02E+06	ND	ND	ND	ND	*ND*
Untargeted (353)	32.27	0.50	0.92	4.06	1825	2.41E+06	1.97E+06	ND	ND	9.65E+05	6.04E+05	*10.67*
Untargeted (355)	32.30	0.27	0.80	4.73	1826	1.23E+06	6.27E+05	ND	ND	ND	1.07E+06	*15.31*
Untargeted (356)	32.39	0.61	1.01	4.07	1830	ND	ND	1.93E+06	5.33E+06	7.30E+06	1.73E+06	*10.65*
Untargeted (358)	32.48	0.90	0.92	5.03	1834	7.04E+06	ND	1.13E+06	5.90E+06	5.86E+06	ND	*ND*
Untargeted (36)	9.49	1.09	1.78	3.34	1047	6.29E+05	3.64E+05	1.97E+05	2.30E+05	ND	2.06E+05	*7.33*
Untargeted (364)	33.02	0.60	1.03	3.17	1856	4.57E+06	3.43E+06	6.22E+06	1.64E+06	ND	ND	*ND*
Untargeted (365)	33.26	0.61	0.84	6.56	1866	1.05E+07	2.53E+06	1.23E+06	4.25E+05	1.33E+06	ND	*ND*
Untargeted (366)	33.29	0.50	1.65	2.51	1868	2.53E+06	2.80E+06	2.30E+06	1.93E+06	1.00E+06	3.51E+05	*12.37*
Untargeted (367)	33.34	0.16	0.73	3.27	1869	6.55E+06	7.63E+06	ND	ND	ND	ND	*ND*
Untargeted (368)	33.37	0.00	1.11	0.00	1871	1.13E+06	ND	ND	ND	ND	ND	*ND*
Untargeted (370)	33.43	0.00	0.86	0.00	1873	ND	4.38E+05	ND	ND	ND	ND	*ND*
Untargeted (371)	33.65	0.59	0.58	4.14	1882	3.30E+05	2.73E+05	8.32E+05	1.44E+06	ND	4.66E+05	*6.33*
Untargeted (374)	33.98	0.54	0.93	4.86	1896	ND	ND	3.41E+06	7.78E+06	8.59E+06	3.06E+06	*12.05*
Untargeted (378)	34.34	0.51	0.62	6.21	1912	7.80E+05	5.28E+05	1.09E+06	1.69E+06	2.16E+06	3.07E+05	*11.33*
Untargeted (380)	34.81	0.56	0.67	1.18	1933	1.48E+06	1.83E+06	ND	ND	1.28E+06	ND	*ND*
Untargeted (382)	35.11	0.54	1.00	1.86	1946	3.89E+06	5.07E+06	1.79E+06	1.22E+06	ND	ND	*ND*
Untargeted (383)	35.15	0.15	0.64	4.72	1947	4.15E+06	2.15E+06	ND	ND	ND	ND	*ND*
Untargeted (384)	35.25	1.11	0.83	2.88	1952	2.57E+06	1.47E+06	ND	ND	ND	ND	*ND*
Untargeted (385)	35.25	0.50	0.92	4.86	1952	ND	ND	1.68E+07	1.86E+07	1.80E+07	9.54E+06	*15.28*
Untargeted (39)	9.84	1.35	1.90	2.58	1057	4.49E+06	2.40E+06	3.07E+06	4.25E+06	4.15E+06	2.26E+06	*16.33*
Untargeted (390)	35.98	0.56	1.09	4.10	1984	7.44E+05	ND	5.30E+06	4.40E+06	3.84E+05	1.20E+06	*24.58*
Untargeted (391)	36.13	0.49	0.93	4.83	1991	ND	ND	1.08E+07	1.60E+07	1.42E+07	7.43E+06	*32.96*
Untargeted (392)	36.58	0.50	0.72	5.21	2011	3.27E+06	9.47E+05	5.25E+07	6.06E+07	5.37E+07	4.27E+07	*9.15*
Untargeted (396)	36.92	0.43	0.56	7.35	2027	7.71E+05	4.94E+05	3.85E+06	4.25E+06	2.00E+06	2.13E+06	*3.33*
Untargeted (398)	37.23	0.48	0.58	7.80	2041	1.70E+06	1.48E+06	ND	ND	5.64E+05	4.80E+05	*18.99*
Untargeted (399)	37.35	0.57	1.02	5.69	2047	4.49E+05	8.31E+05	1.43E+06	2.05E+05	6.23E+05	2.94E+05	*4.27*
Untargeted (402)	37.64	0.49	0.89	4.90	2060	ND	ND	1.72E+07	1.81E+07	1.54E+07	7.35E+06	*3.27*
Untargeted (404)	37.79	0.14	0.90	2.57	2067	3.40E+06	3.46E+06	ND	ND	ND	ND	*ND*
Untargeted (405)	38.21	0.30	0.93	4.71	2087	1.18E+06	1.52E+06	ND	3.38E+05	ND	2.75E+05	*11.33*
Untargeted (407)	38.27	0.24	0.72	5.72	2089	1.03E+06	1.33E+06	ND	ND	6.97E+05	ND	*ND*
Untargeted (410)	38.81	0.20	1.67	2.71	2114	ND	ND	ND	3.27E+06	1.71E+07	ND	*ND*
Untargeted (413)	39.28	0.44	1.03	3.82	2136	1.28E+06	1.61E+06	7.23E+06	8.93E+06	5.94E+06	5.15E+06	*25.64*
Untargeted (416)	39.69	0.47	0.98	4.29	2155	ND	ND	2.47E+06	2.34E+06	8.40E+05	2.40E+06	*12.23*
Untargeted (420)	40.13	0.45	1.34	3.57	2176	5.30E+06	ND	2.59E+06	5.04E+06	6.28E+06	3.64E+06	*16.49*
Untargeted (421)	40.36	0.34	0.90	1.15	2186	ND	ND	1.58E+06	5.33E+05	9.15E+05	1.94E+05	*4.33*
Untargeted (423)	40.80	0.40	0.74	5.21	2213	1.06E+06	1.01E+06	9.36E+05	1.74E+06	1.38E+06	2.36E+05	*9.33*
Untargeted (424)	41.08	0.46	1.05	4.58	2227	1.38E+06	1.96E+06	2.18E+06	1.43E+06	1.40E+06	7.15E+05	*14.82*
Untargeted (426)	41.43	0.07	0.74	3.13	2244	2.25E+06	2.06E+06	ND	ND	ND	ND	*ND*
Untargeted (427)	41.47	0.28	0.69	4.86	2245	ND	ND	4.09E+06	1.82E+06	3.34E+05	ND	*ND*
Untargeted (433)	41.98	0.39	1.03	4.74	2271	9.52E+05	1.15E+06	1.45E+06	7.58E+05	4.35E+05	2.84E+05	*6.33*
Untargeted (435)	42.18	0.69	0.97	6.86	2280	8.26E+05	4.58E+05	1.00E+06	9.76E+05	7.86E+05	1.26E+05	*6.33*
Untargeted (436)	42.36	0.56	0.59	1.99	2289	3.84E+05	7.57E+05	1.04E+06	5.73E+05	7.30E+05	1.12E+05	*18.17*
Untargeted (437)	42.55	0.37	1.39	3.55	2298	ND	ND	2.86E+06	3.41E+06	3.65E+06	1.16E+06	*15.42*
Untargeted (438)	42.56	0.38	0.68	7.29	2299	8.26E+05	2.69E+05	ND	4.09E+05	9.13E+05	1.10E+05	*8.52*
Untargeted (439)	42.66	0.40	0.55	9.17	2304	3.28E+05	3.77E+05	1.37E+06	7.85E+05	5.95E+05	ND	*ND*

Untargeted (440)	42.75	0.38	0.80	5.19	2309	4.75E+05	9.56E+05	1.33E+06	1.26E+06	1.42E+06	3.03E+05	*30.99*
Untargeted (444)	43.35	0.60	0.65	1.07	2341	2.84E+05	1.09E+06	9.90E+05	5.41E+05	6.08E+05	1.97E+05	*24.19*
Untargeted (445)	43.37	0.45	0.76	8.18	2342	7.40E+05	4.65E+05	1.17E+06	7.63E+05	6.86E+05	4.04E+05	*11.45*
Untargeted (446)	43.48	0.23	1.60	2.47	2348	ND	ND	ND	1.47E+06	6.17E+06	ND	*ND*
Untargeted (447)	43.55	0.38	1.16	5.30	2352	ND	1.13E+06	2.03E+06	1.27E+06	9.90E+05	9.79E+05	*21.17*
Untargeted (451)	44.13	0.35	0.66	6.45	2383	8.40E+05	1.87E+06	2.54E+06	2.17E+06	1.92E+06	2.22E+05	*9.90*
Untargeted (452)	44.74	0.32	1.07	4.62	2415	ND	ND	ND	1.57E+05	ND	4.65E+05	*30.37*
Untargeted (453)	44.80	0.38	0.65	7.38	2419	ND	2.96E+06	ND	ND	2.69E+06	1.35E+06	*18.99*
Untargeted (454)	45.28	0.69	0.64	9.62	2444	ND	1.59E+06	ND	3.79E+05	1.72E+06	3.40E+05	*30.61*
Untargeted (455)	45.42	0.32	1.47	3.63	2452	2.99E+06	ND	2.84E+06	2.94E+06	2.50E+06	7.55E+05	*19.01*
Untargeted (456)	45.43	0.35	0.90	6.25	2452	2.92E+06	2.79E+06	1.09E+06	2.03E+06	3.18E+06	9.13E+05	*5.50*
Untargeted (457)	45.58	0.48	1.13	5.39	2460	7.51E+05	7.46E+05	2.24E+06	1.55E+06	2.29E+06	2.14E+05	*9.31*
Untargeted (458)	45.59	0.33	1.22	4.97	2461	ND	ND	1.49E+05	2.20E+06	2.11E+06	4.47E+05	*15.91*
Untargeted (461)	46.38	0.33	0.81	5.55	>2500	ND	ND	1.72E+06	1.21E+06	8.86E+05	3.62E+05	*5.86*
Untargeted (463)	46.73	0.24	0.95	4.42	>2500	ND	ND	ND	ND	ND	8.35E+05	*21.92*
Untargeted (464)	46.79	0.38	0.85	5.21	>2500	ND	ND	1.32E+06	9.96E+05	1.11E+05	8.64E+05	*16.73*
Untargeted (466)	47.01	0.32	0.66	6.41	>2500	3.18E+06	4.27E+06	2.92E+06	2.97E+06	2.71E+06	1.35E+06	*25.92*
Untargeted (467)	47.06	0.31	0.84	5.55	>2500	ND	ND	1.28E+06	9.02E+05	1.23E+06	5.45E+05	*25.22*
Untargeted (468)	47.19	0.28	1.68	3.26	>2500	9.21E+05	ND	1.28E+06	3.83E+06	7.76E+06	ND	*ND*
Untargeted (469)	47.25	0.11	1.26	3.30	>2500	ND	ND	ND	ND	2.20E+06	ND	*ND*
Untargeted (471)	47.99	0.16	1.53	3.74	>2500	ND	ND	2.09E+06	4.78E+06	1.68E+07	ND	*ND*
Untargeted (472)	48.22	0.47	0.79	1.11	>2500	3.99E+05	6.45E+06	2.02E+06	3.83E+06	3.42E+06	1.80E+05	*18.83*
Untargeted (474)	49.10	0.40	1.41	3.15	>2500	ND	ND	9.35E+06	1.13E+07	1.08E+07	2.10E+05	*9.25*
Untargeted (476)	51.03	0.24	0.75	6.08	>2500	ND	ND	9.23E+06	7.51E+06	8.07E+06	1.23E+06	*10.64*
Untargeted (51)	11.30	1.01	1.65	1.90	1102	3.63E+06	3.88E+06	4.88E+05	ND	ND	2.92E+05	*11.46*
Untargeted (53)	11.52	1.02	1.16	3.42	1109	2.66E+06	2.13E+06	2.60E+06	3.32E+06	2.08E+06	5.00E+05	*11.96*
Untargeted (55)	11.80	2.12	1.61	2.92	1118	1.01E+06	9.82E+05	6.56E+05	1.01E+06	ND	5.48E+05	*5.21*
Untargeted (56)	11.83	1.25	1.00	3.39	1119	3.71E+06	2.92E+06	9.69E+05	1.05E+06	1.62E+06	4.75E+05	*7.12*
Untargeted (69)	12.91	1.73	1.24	3.42	1154	2.37E+06	1.73E+06	ND	ND	2.41E+06	8.46E+05	*8.27*
Untargeted (70)	13.02	1.14	1.54	2.70	1157	4.07E+06	7.01E+06	4.66E+06	5.95E+06	4.26E+06	2.86E+06	*9.56*
Untargeted (75)	13.37	1.23	1.43	3.50	1168	6.32E+06	6.15E+06	6.04E+06	5.80E+06	5.13E+06	4.63E+06	*29.87*
Untargeted (77)	13.56	0.23	1.11	2.34	1175	9.88E+05	1.90E+06	ND	ND	ND	ND	*ND*
Untargeted (78)	13.58	1.17	1.37	3.34	1175	3.16E+06	5.50E+06	7.93E+06	8.06E+06	5.80E+06	4.01E+06	*9.13*
Untargeted (79)	13.60	1.27	1.97	2.61	1176	1.17E+06	1.67E+06	8.00E+06	5.29E+06	4.14E+06	2.66E+06	*8.78*
Untargeted (81)	13.83	1.31	1.45	3.60	1183	1.27E+06	ND	3.10E+06	5.40E+06	1.61E+06	1.58E+06	*28.91*
Untargeted (93)	14.93	1.19	1.34	3.12	1219	8.02E+05	9.92E+05	9.46E+06	8.04E+06	1.14E+06	5.67E+06	*5.81*
Untargeted (95)	15.08	1.74	1.59	1.73	1223	1.97E+07	1.03E+07	3.83E+05	1.70E+06	ND	ND	*ND*
Untargeted (98)	15.30	2.20	1.11	1.27	1230	1.86E+06	4.15E+06	ND	ND	2.58E+06	ND	*ND*
α-Humulene	29.02	0.61	1.68	2.95	1693	1.08E+07	7.40E+06	1.56E+07	1.53E+07	1.23E+07	9.01E+06	*12.46*
α-Methyl-γ-butyrolactone	26.85	0.10	0.77	4.17	1623	3.72E+06	2.55E+06	ND	ND	ND	ND	*ND*
α-Terpineol	28.66	0.64	0.88	4.58	1681	ND	ND	1.07E+07	9.70E+06	7.52E+06	5.39E+06	*14.07*
β-Bisabolene	29.65	0.64	1.55	3.36	1717	7.44E+06	4.41E+06	1.06E+07	1.29E+07	4.11E+06	5.90E+06	*16.38*
β-Caryophyllene	26.39	0.74	1.84	2.60	1608	5.72E+06	4.81E+06	3.54E+06	3.81E+06	3.60E+06	1.08E+06	*8.81*
β-Cyclocitral	26.93	0.69	1.14	3.44	1626	1.34E+07	1.10E+07	1.72E+07	1.98E+07	1.68E+07	1.21E+07	*7.33*
β-Damascenone	31.90	0.59	1.14	3.80	1809	ND	ND	2.81E+07	2.77E+07	2.18E+07	1.66E+07	*11.08*
β-Ionone	34.64	0.53	1.21	3.40	1925	1.93E+07	1.27E+07	7.46E+06	7.10E+06	6.04E+06	3.73E+06	*20.60*
β-Ocimene	16.08	1.06	1.37	4.43	1255	1.99E+06	7.88E+06	6.41E+06	4.41E+06	3.74E+06	2.78E+06	*17.19*
γ-Butyrolactone	27.09	0.71	0.71	4.87	1631	1.82E+06	1.90E+06	8.83E+06	1.09E+07	1.17E+06	5.57E+06	*7.57*
γ-Hexalactone	28.99	0.65	0.82	4.49	1692	1.05E+07	1.52E+07	3.46E+07	3.19E+07	2.46E+07	2.09E+07	*10.85*
γ-Nonalactone	36.64	0.50	0.96	4.30	2014	1.05E+07	9.04E+06	1.07E+08	1.52E+08	1.28E+08	9.59E+07	*22.21*

Supplementary Table2: Target components and untargeted features mapped through all analyzed samples. Target analytes, reported with corresponding CAS registry number, were identified according to criteria of spectral similarity (DMF above 900 and RMF above 950) and I^T tolerance of ± 15 units. Analytes are listed with retention times (^1t_R, ^2t_R) and corresponding precision data expressed as %RSD across all analyses (n=35), experimental linear retention index (I^T) and tabulated I^T (NIST database https://webbook.nist.gov/chemistry/), Fisher ratio (F) values calculated for all classes (F all). When features were invariant (e.g., undetected) within a class, the Fisher ratio cannot be computed and in table is reported as "ND". This table complements Table 2 of the manuscript.

Feature ID	CAS	^1t_R min	%RSD	^2t_R s	%RSD	I^T exp	I^T tab	*F* all
**Alcohols**								
Ethanol	64-17-5	7.21	0.13	2.33	1.24	948	944	ND
2-Butanol	78-92-2	9.30	1.22	0.64	5.89	1041	1036	ND
1-Propanol	71-23-8	9.66	1.16	0.59	6.48	1052	1051	ND
2-Methyl-1-propanol (isobutanol)	78-83-1	11.21	1.27	0.63	5.77	1099	1101	ND
3-Pentanol	584-02-1	11.64	1.29	0.71	5.63	1113	1111	ND
3-Methyl-2-butanol	598-75-4	12.00	1.29	0.71	5.78	1124	1118	21
1-Butanol	71-36-3	12.68	1.18	0.64	5.51	1146	1146	34
2-Methyl-3-pentanol	565-67-3	13.13	1.25	0.79	5.18	1161	1167	ND
1-Penten-3-ol	616-25-1	13.23	1.28	0.64	6.27	1164	1164	ND
3-Methyl-1-butanol (isoamyl alcohol)	123-51-3	14.64	1.21	0.69	6.19	1209	1211	ND
2-Hexanol	626-93-7	15.04	1.17	0.76	5.70	1222	1222	ND
1-Pentanol	71-41-0	15.95	1.12	0.69	5.40	1251	1252	ND
4-Heptanol	589-55-9	16.99	1.07	0.85	4.27	1284	1285	ND
(E)-2-Penten-1-ol	1576-96-1	17.88	1.02	0.63	5.98	1313	1310	ND
2-Heptanol	543-49-7	18.08	1.08	0.82	4.50	1320	1319	39
(Z)-2-Penten-1-ol	1576-95-0	18.10	1.04	0.63	5.96	1320	1317	12
1-Hexanol	111-27-3	19.07	1.05	0.73	5.13	1352	1344	18
(E)-3-Hexen-1-ol	928-97-2	20.07	0.94	0.69	5.36	1385	1373	5
3-Octanol	589-98-0	20.31	0.88	0.91	4.27	1393	1398	ND
(Z)-2-Hexen-1-ol	928-94-9	20.66	1.43	0.69	7.27	1405	1401	25
4-Hexen-1-ol	6126-50-7	20.76	0.76	0.67	5.51	1408	1408	73
(E)-2-Hexen-1-ol	928-95-0	20.86	1.31	0.67	5.19	1412	1411	ND
2-Octanol	123-96-6	21.07	0.86	0.87	4.23	1419	1405	ND
1-Octen-3-ol	3391-86-4	21.99	0.85	0.78	4.76	1451	1450	79
6-Methyl-5-hepten-2-ol	1569-60-4	22.31	0.82	0.79	4.52	1462	1465	8
4-Nonanol	5932-79-6	22.84	0.53	0.97	2.03	1481	1479	10
2-Ethylhexanol	104-76-7	23.17	0.84	0.82	5.84	1492	1484	ND
(Z)-4-Hepten-1-ol	20851-55-2	23.46	0.77	0.72	5.21	1502	1502	ND
(E)-2-Hepten-1-ol	33467-76-4	23.61	1.13	0.75	9.91	1507	1504	ND
1-Octanol	111-87-5	24.97	0.77	0.81	5.07	1557	1555	ND
2,3-Butanediol	513-85-9	25.38	0.72	0.55	6.89	1572	1583	ND
2,4-Hexadien-1-ol	111-28-4	25.89	0.20	0.61	3.79	1591	1588	6
(5E)-3,7-Dimethyl-1,5,7-octatrien-3-ol (hotrienol)	53834-70-1	26.46	0.70	0.81	5.54	1611	1602	ND
(E)-2-Octen-1-ol	18409-17-1	26.52	0.68	0.75	4.96	1613	1611	ND

2-(2-Ethoxyethoxy)ethanol	111-90-0	26.86	0.61	0.68	3.65	1623	1615	ND
1-Nonanol	143-08-8	27.63	0.67	0.85	4.90	1648	1663	ND
6-Undecanol	23708-56-7	27.80	0.12	0.81	4.28	1654	1640	ND
(Z)-3-Nonen-1-ol	10340-23-5	28.51	0.18	0.81	2.86	1677	1682	ND
1-Decanol	112-30-1	30.24	0.64	0.90	4.33	1741	1748	ND
2-(2-Butoxyethoxy)ethanol	112-34-5	31.18	0.55	0.77	5.05	1780	1786	ND
1-Tetradecanol	112-72-1	39.78	0.44	1.06	3.68	2159	2157	ND
**Aldehydes**								
2-Methylpropanal	78-84-2	5.44	0.48	0.66	5.15	821	819	ND
Acrolein	107-02-8	5.86	0.51	0.58	6.95	834	840	ND
3-Methylbutanal	590-86-3	6.99	0.81	0.82	5.30	929	936	42
2-Butenal	4170-30-3	9.94	1.31	0.75	7.01	1060	1061	210
Hexanal	66-25-1	11.02	1.20	1.04	3.97	1093	1098	22
(E)-2-Pentenal	1576-87-0	12.40	1.19	0.87	4.31	1137	1147	63
(E)-3-Hexenal	69112-21-6	12.83	0.00	0.89	4.62	1151	1146	ND
(Z)-3-Hexenal	6789-80-6	13.13	0.80	0.88	5.38	1161	1158	16
2-Methyl-2-pentenal	623-36-9	13.46	1.24	0.97	5.30	1171	1171	ND
Heptanal	111-71-7	14.12	1.27	1.17	3.60	1193	1190	ND
3-Methyl-2-butenal	107-86-8	14.63	1.16	0.86	6.30	1209	1212	15
(Z)-2-Hexenal	16635-54-4	14.78	0.20	0.96	3.85	1214	1214	5
(E)-2-Hexenal	6728-26-3	15.15	1.10	0.98	4.21	1226	1220	ND
Octanal	124-13-0	17.35	1.09	1.25	3.10	1296	1291	ND
(E)-2-Heptenal	18829-55-5	18.42	1.02	1.05	3.69	1331	1318	ND
Nonanal	124-19-6	20.51	0.92	1.29	3.12	1400	1392	9
2,4-Hexadienal	80466-34-8	20.76	0.84	0.82	4.98	1409	1402	ND
(Z)-2-Octenal	20664-46-4	20.91	0.26	1.10	2.89	1414	1413	9
2-Furancarboxaldehyde (furfural)	98-01-1	21.50	0.85	0.63	5.71	1434	1437	ND
(E)-2-Octenal	2548-87-0	21.56	0.81	1.10	3.50	1436	1434	20
(E,Z)-2,4-heptadienal	4313-02-4	22.52	0.84	0.89	4.28	1469	1464	14
2,4-Heptadienal	5910-85-0	23.34	0.77	0.88	4.42	1498	1489	ND
Decanal	112-31-2	23.54	0.76	1.34	3.10	1505	1505	ND
(E)-2-Nonenal	18829-56-6	24.58	0.85	1.17	2.86	1543	1530	153
(E,Z)-2,6-Nonadienal	557-48-2	26.06	0.58	1.01	3.23	1597	1590	ND
β-Cyclocitral	432-25-7	26.93	0.69	1.14	3.44	1626	1611	ND
(E)-2-Decenal	3913-81-3	27.45	0.68	1.20	3.40	1643	1625	ND
(2Z)-3,7-Dimethyl-2,6-octadienal (neral)	106-26-3	28.69	0.60	1.07	3.35	1683	1663	ND
2,4-Nonadienal	6750-03-4	29.09	0.21	0.98	3.77	1695	1668	ND
Dodecanal	112-54-9	29.17	0.63	1.42	3.16	1698	1708	ND
(E)-2-Undecenal	53448-07-0	30.33	0.47	1.24	3.54	1745	1755	ND
(E,Z)-2,4-decadienal	25152-83-4	31.35	1.37	1.03	3.97	1786	1778	ND
Tridecanal	10486-19-8	31.78	0.64	1.46	3.13	1804	1821	27
Tetradecanal	124-25-4	34.25	0.52	1.49	2.94	1908	1920	ND
trans-4,5-Epoxy-(E)-2-decenal	134454-31-2	35.95	0.88	0.89	6.36	1983	1995	ND

Pentadecanal	2765-11-9	36.78	0.15	1.54	1.54	2020	2040	ND
**Aromatics**								
Toluene	108-88-3	9.81	1.14	0.90	4.86	1056	1054	ND
Ethylbenzene	100-41-4	12.39	0.53	1.05	2.69	1137	1136	7
p-Xylene	106-42-3	12.52	1.15	1.06	4.16	1141	1142	ND
m-Xylene	108-38-3	12.69	1.23	1.04	3.65	1147	1143	ND
o-Xylene	95-47-6	14.05	1.09	1.03	3.87	1190	1188	15
Propylbenzene	103-65-1	14.92	0.87	1.16	3.80	1218	1213	59
1-Ethyl-2-methylbenzene	611-14-3	15.43	1.00	1.16	4.08	1235	1235	152
1,2,4-Trimethylbenzene	95-63-6	17.07	1.00	1.12	3.44	1287	1287	ND
Benzaldehyde	100-52-7	24.24	0.75	0.75	4.87	1531	1529	ND
Methyl benzoate	93-58-3	26.98	0.64	0.81	5.00	1627	1631	ND
Phenylacetaldehyde	122-78-1	27.43	0.67	0.76	4.83	1642	1625	12
Acetophenone	98-86-2	27.65	0.82	0.79	3.98	1649	1634	11
Ethyl benzoate	93-89-0	28.12	0.65	0.88	4.41	1664	1673	ND
1,3-Dimethoxybenzene	151-10-0	30.08	0.63	0.78	4.97	1735	1730	ND
Naphthalene	91-20-3	30.09	0.17	0.85	2.73	1735	1743	ND
Ethyl phenylacetate	101-97-3	30.99	0.59	0.86	5.04	1772	1775	9
2-Methoxyphenol (guaiacol)	90-05-1	32.68	0.63	0.63	6.80	1842	1860	ND
Propyl phenylacetate	4606-15-9	32.91	0.25	0.90	3.82	1852	1848	17
Benzyl alcohol	100-51-6	33.00	0.56	0.60	6.62	1855	1864	ND
Ethyl 3-phenylpropanoate	2021-28-5	33.40	0.59	0.91	4.68	1872	1892	ND
2-Phenylethanol	60-12-8	33.84	0.54	0.65	5.64	1890	1890	ND
2-Methoxy-4-methylphenol	93-51-6	34.88	0.54	0.68	6.03	1936	1938	ND
Phenol	108-95-2	35.88	0.51	0.54	7.72	1980	1994	ND
p-Cresol	106-44-5	37.49	0.45	0.57	6.71	2053	2057	ND
2-Methoxy-4-propylphenol (4-propylguaiacol)	2785-87-7	38.23	0.46	0.75	5.58	2087	2084	ND
(Z)-3-Hexenyl benzoate	25152-85-6	38.75	0.45	1.00	4.24	2112	2120	ND
Phenoxyethanol	122-99-6	38.85	0.44	0.64	6.54	2116	2115	27
2,3-Dimethylphenol	526-75-0	39.50	0.45	0.60	6.01	2146	2155	ND
4-Ethenyl-2-methoxyphenol (4-vinylguaiacol)	7786-61-0	39.96	0.43	0.68	5.95	2168	2175	ND
Ethyl 2-hydroxy-3-phenylpropanoate	15399-05-0	41.75	0.40	0.75	5.43	2259	2249	ND
2-Methoxy-4-(1-propen-1-yl)phenol (isoeugenol)	97-54-1	42.90	0.37	0.71	6.73	2317	2316	211
**Acids**								
Acetic acid	64-19-7	22.04	1.22	0.47	8.15	1453	1452	ND
Propionic acid	79-09-4	24.73	1.04	0.49	7.54	1548	1544	27
Isobutyric acid	79-31-2	25.66	0.60	0.53	9.96	1583	1580	8
Butyric acid	107-92-6	27.39	0.36	0.49	7.32	1641	1624	ND
Isovaleric acid	503-74-2	28.38	0.64	0.52	7.46	1672	1653	ND
Pentanoic acid	109-52-4	30.07	0.64	0.53	7.37	1734	1733	18
Hexanoic acid	142-62-1	32.66	0.53	0.54	7.29	1841	1840	5
Heptanoic acid	111-14-8	35.21	0.47	0.56	7.03	1950	1960	ND
Octanoic acid	124-07-2	37.76	0.07	0.60	3.85	2066	2068	ND

Nonanoic acid	112-05-0	39.86	0.40	0.61	6.30	2163	2173	6
Decanoic acid	334-48-5	41.93	0.39	0.64	6.35	2268	2270	ND
Dodecanoic acid	143-07-7	45.92	0.57	0.70	6.46	2478	2469	ND
**Esters**								
Methyl acetate	79-20-9	5.64	0.68	0.60	6.24	827	832	ND
Ethyl acetate	141-78-6	6.48	0.84	0.71	5.63	854	870	20
Ethyl propionate	105-37-3	7.87	1.07	0.87	4.24	993	964	ND
Propyl acetate	109-60-4	8.23	1.23	0.88	5.22	1008	996	ND
Methyl butyrate	623-42-7	8.59	0.31	0.86	4.31	1019	1004	ND
Methyl isovalerate	556-24-1	9.45	0.00	0.98	2.82	1045	1025	ND
Propyl propionate	106-36-5	10.01	1.25	1.08	3.64	1062	1056	ND
Ethyl 2-methylbutanoate	7452-79-1	10.22	1.24	1.22	3.33	1069	1063	ND
Ethyl isovalerate	108-64-5	10.67	1.21	1.18	3.34	1083	1079	13
2-Pentyl acetate	626-38-0	10.80	1.23	1.15	3.71	1087	1080	48
Methyl pentanoate	624-24-8	11.14	1.05	1.02	2.80	1097	1090	8
Isoamyl acetate	123-92-2	12.25	1.26	1.14	3.79	1133	1126	ND
Propyl butyrate	105-66-8	12.32	0.66	1.22	3.65	1135	1133	ND
Ethyl pentanoate	539-82-2	12.63	1.22	1.19	3.55	1145	1142	ND
Propyl isovalerate	557-00-6	13.22	1.00	1.37	2.75	1164	1153	535
Amyl acetate	628-63-7	13.80	1.26	1.15	4.10	1182	1177	6
Methyl hexanoate	106-70-7	14.23	1.21	1.13	3.96	1196	1190	94
Isoamyl propionate	105-68-0	14.29	1.17	1.34	3.60	1198	1192	6
Propyl pentanoate	141-06-0	15.24	1.13	1.36	3.64	1228	1217	ND
Butyl butyrate	109-21-7	15.34	0.00	1.35	0.00	1232	1230	ND
Ethyl hexanoate	123-66-0	15.67	1.09	1.30	3.13	1242	1240	ND
Methyl (Z)-3-hexenoate	13894-62-7	16.49	1.08	0.98	4.11	1268	1265	15
2-Heptyl acetate	5921-82-4	16.55	1.06	1.39	3.53	1270	1255	ND
Isoamyl butyrate	106-27-4	16.69	0.84	1.48	2.56	1275	1270	ND
Hexyl acetate	142-92-7	16.88	1.05	1.22	2.98	1281	1276	ND
Methyl (E)-2-hexenoate	13894-63-8	17.60	0.17	1.05	0.98	1304	1305	35
(E)-3-Hexenyl acetate	3681-82-1	18.02	0.95	1.07	3.94	1318	1306	14
(Z)-3-Hexenyl acetate	3681-71-8	18.25	1.08	1.06	3.68	1325	1319	ND
Propyl hexanoate	626-77-7	18.28	0.97	1.42	2.96	1326	1316	17
Ethyl heptanoate	106-30-9	18.70	0.96	1.36	3.17	1340	1332	ND
Propanoic acid, 2-hydroxy-, ethyl ester (ethyl lactate)	687-47-8	18.96	0.98	0.67	5.65	1349	1356	ND
Ethyl 2-hexenoate	1552-67-6	19.10	1.01	1.14	3.59	1353	1343	17
Isobutyl hexanoate	105-79-3	19.30	0.93	1.58	2.98	1360	1351	ND
Heptyl acetate	112-06-1	19.92	0.98	1.28	3.71	1381	1370	ND
Ethyl (4E)-4-heptenoate	54340-70-4	20.22	1.13	1.19	3.83	1390	1382	20
Butyl-(Z)-3-hexenoate	69668-84-4	21.14	0.90	1.32	3.47	1422	1421	ND
Ethyl 2-hydroxy-3-methyl butyrate	2441-06-7	21.51	0.94	0.80	1.13	1434	1422	ND
Ethyl octanoate	106-32-1	21.72	0.84	1.42	3.08	1442	1440	ND
Isoamyl hexanoate	2198-61-0	22.43	0.79	1.59	2.78	1466	1453	ND

(E)-3-Hexenyl butyrate	53398-84-8	22.53	0.97	1.30	5.05	1470	1466	12
Octyl acetate	112-14-1	22.90	0.75	1.33	3.07	1483	1480	ND
Ethyl 4-octenoate	138234-61-4	22.99	0.77	1.24	4.03	1485	1470	ND
Ethyl (E,E)-2,4-Hexadienoate (ethyl sorbate)	2396-84-1	23.76	0.75	0.95	4.51	1513	1501	ND
Ethyl nonanoate	123-29-5	24.56	0.74	1.46	2.99	1542	1530	ND
Isopentyl 2-hydroxypropanoate (isoamyl lactate)	19329-89-6	25.47	0.72	0.81	4.98	1576	1583	ND
Nonyl acetate	143-13-5	25.70	0.68	1.38	3.12	1584	1582	11
1,2-Propanediol, 2-acetate	6214-01-3	26.55	0.73	0.60	7.24	1613	1621	ND
Hexyl hexanoate	6378-65-0	26.61	0.69	1.59	2.74	1615	1599	ND
α-Methyl-γ-butyrolactone	1679-47-6	26.85	0.10	0.77	4.17	1623	1625	20
γ-Butyrolactone	96-48-0	27.09	0.71	0.71	4.87	1631	1635	ND
Ethyl decanoate	110-38-3	27.29	0.64	1.49	4.33	1637	1624	ND
(Z)-3-Hexenyl hexanoate	31501-11-8	27.80	0.67	1.39	3.19	1654	1638	ND
Butanedioic acid, 1,4-diethyl ester (diethyl succinate)	123-25-1	28.28	0.67	0.87	5.02	1669	1677	ND
γ-Hexalactone	695-06-7	28.99	0.65	0.82	4.49	1692	1689	ND
Propyl decanoate	30673-60-0	29.44	0.28	1.59	2.73	1709	1722	ND
Propanoic acid, 2-hydroxy-, (3Z)-3-hexenyl ester ((E)-3-hexenyl lactate)	61931-81-5	29.45	0.63	1.22	3.76	1709	1727	ND
3-Methyl-2(5H)-furanone	22122-36-7	29.46	0.48	0.71	5.88	1709	1713	ND
Benzyl acetate	140-11-4	29.65	0.60	0.79	4.83	1717	1733	ND
Methyl 2-hydroxy-benzoate (methyl salicylate)	119-36-8	30.91	0.58	0.81	5.01	1768	1757	ND
2-Phenylethyl acetate	103-45-7	31.79	0.59	0.85	4.66	1804	1820	6
Ethyl dodecanoate	106-33-2	32.44	0.57	1.57	3.15	1832	1848	ND
Propyl dodecanoate	3681-78-5	34.33	0.36	1.64	2.39	1911	1927	ND
γ-Nonalactone	104-61-0	36.64	0.50	0.96	4.30	2014	2020	ND
Isopropyl tetradecanoate (isopropyl myristate)	110-27-0	36.87	0.47	1.77	2.49	2025	2026	10
Ethyl tetradecanoate (ethyl myristate)	124-06-1	37.08	0.47	1.63	2.96	2034	2046	ND
Methyl hexadecanoate (methyl palmitate)	112-39-0	40.61	0.42	1.56	3.01	2204	2210	ND
Ethyl hexadecanoate (Ethyl palmitate)	628-97-7	41.36	0.39	1.69	3.15	2240	2254	ND
Ethyl (E)-9-hexadecenoate	54546-22-4	41.94	0.45	1.56	3.54	2269	2277	ND
Propyl hexadecanoate (propyl palmitate)	2239-78-3	43.04	0.37	1.76	2.81	2324	2335	ND
Ethyl (Z)-9-octadecenoate (Ethyl oleate)	111-62-6	45.69	0.35	1.62	3.05	2466	2470	ND
Ethyl (Z,Z)-9,12-Octadecadienoate (ethyl linoleate)	544-35-4	46.52	0.33	1.51	3.81	>2500		ND
Ethyl (Z,Z,Z)-9,12,15-Octadecatrienoate (ethyl linolenate)	1191-41-9	47.70	0.31	1.39	3.76	>2500		ND
**Heterocyclic compounds**								
2-Methylfuran	534-22-5	6.24	0.00	0.66	0.00	846	850	ND
2-Ethylfuran	3208-16-0	7.78	0.90	0.79	5.75	987	965	ND
2-Pentylfuran	3777-69-3	15.56	1.12	1.19	3.63	1239	1235	32
2-Furanmethanol (furfuryl alcohol)	98-00-0	27.68	0.76	0.55	6.78	1650	1651	ND
2-Ethyl-3-methyl maleimide	20189-42-8	41.56	0.41	0.62	5.80	2250	2260	ND
**Hydrocarbons**								
Propane	74-98-6	4.49	2.45	0.50	6.96	NC	300	ND
Heptane	142-82-5	4.49	0.00	0.86	4.36	700	700	ND
Octane	111-65-9	5.25	0.22	1.30	3.63	800	800	5

Nonane	111-84-2	6.59	0.75	1.90	3.14	900	900	10
(E)-1,3-Octadiene	1002-33-1	7.80	0.76	1.27	4.28	989	958	234
Undecane	1120-21-4	11.24	1.63	2.98	2.69	1100	1100	ND
1-Undecene	821-95-4	12.67	1.03	2.36	1.81	1146	1142	ND
Dodecane	112-40-3	14.38	1.12	3.06	2.09	1200	1200	ND
Tridecane	629-50-5	17.48	1.00	3.05	2.17	1300	1300	ND
1-Tetradecene	1120-36-1	21.84	1.21	2.46	2.49	1446	1428	23
Tetradecane	629-59-4	20.51	0.86	2.99	1.46	1400	1400	ND
Pentadecane	629-62-9	23.41	0.82	2.97	2.47	1500.00	1500	ND
Hexadecane	544-76-3	26.13	0.64	2.88	1.98	1600	1600	ND
**Ketones**								
Acetone	67-64-1	5.48	0.00	0.58	6.38	822	821	ND
Methyl ethyl ketone	78-93-3	6.71	0.00	0.72	4.94	909	905	ND
2-Pentanone	107-87-9	8.28	0.96	0.87	2.96	1010	1007	ND
Butanedione	431-03-8	8.37	1.24	0.64	6.45	1013	993	ND
2-Methyl-3-pentanone	565-69-5	8.72	1.26	1.04	4.09	1023	1003	ND
1-Penten-3-one	1629-58-9	9.33	1.22	0.80	5.36	1042	1024	ND
2,3-Pentanedione	600-14-6	10.56	0.00	0.78	0.00	1079	1070	ND
2-Heptanone	110-43-0	14.00	1.13	1.13	3.17	1189	1184	24
6-Methyl-2-heptanone	928-68-7	15.87	0.00	1.23	0.00	1248	1236	ND
5-Methyl-3-heptanone	541-85-5	16.26	1.04	1.30	3.05	1261	1265	6
2-Octanone	111-13-7	17.26	1.15	1.23	2.62	1293	1291	5
3-Hydroxy-2-butanone (acetoin)	513-86-0	17.32	1.14	0.64	5.74	1295	1287	8
2,2,6-Trimethylcyclohexanone	2408-37-9	18.27	1.03	1.31	3.03	1326	1320	81
4-Nonanone	4485-09-0	18.47	1.13	1.41	3.59	1333	1322	7
(Z)-6-Octen-2-one	74810-53-0	18.73	0.48	1.06	3.24	1341	1316	20
6-Methyl-5-hepten-2-one	110-93-0	18.84	0.94	1.04	3.83	1345	1340	32
2-Nonanone	821-55-6	20.49	0.52	1.28	1.91	1399	1386	ND
(E,Z)-3,5-Octadien-2-one	30086-02-3	24.21	0.18	0.93	2.79	1529	1513	39
(E,E)-3,5-Octadien-2-one	30086-02-3	25.61	0.17	0.92	1.92	1581	1570	ND
2-Undecanone	112-12-9	26.41	0.20	1.37	1.69	1609	1606	33
6,10-Dimethyl-2-undecanone	1604-34-8	28.59	0.64	1.50	3.18	1679	1660	ND
6,10,14-Trimethyl-2-pentadecanone	502-69-2	38.70	0.44	1.71	2.99	2110	2110	ND
**Others**								
Styrene	100-42-5	16.30	1.09	0.88	4.34	1262	1264	ND
Hexanenitrile	628-73-9	17.69	1.02	0.93	4.27	1307	1303	51
1-Nitropentane	628-05-7	20.78	0.97	0.88	4.05	1409	1409	ND
1-Nitrohexane	646-14-0	23.86	0.18	0.94	3.38	1517	1511	ND
2,3,3a,4,5,7a-Hexahydro-3,6-dimethylbenzofuran	70786-44-6	23.95	0.75	1.22	3.33	1520	1527	15
Dimethyl Sulfoxide	67-68-5	24.97	0.00	0.70	1.42	1557	1560	ND
3,5,5-Trimethyl-2-cyclohexene-1,4-dione (ketoisophorone)	1125-21-9	28.95	0.09	0.89	2.60	1691	1676	23
3,4-Dimethyl-2,5-furandione	766-39-2	30.02	0.33	0.80	5.50	1732	1714	ND
Bis(2-hydroxypropyl) ether	110-98-5	31.90	0.74	0.56	3.77	1809	1817	ND

5,6,7,7a-Tetrahydro-4,4,7a-trimethyl-2(4*H*)-benzofuranone	15356-74-8	43.16	0.38	1.02	4.15	2331	2325	ND
Hexadecanolide	109-29-5	43.85	0.44	1.58	3.16	2368	2367	ND
**Terpenes**								
Limonene	138-86-3	14.48	1.12	1.51	3.04	1204	1194	ND
β-Ocimene	13877-91-3	16.08	1.06	1.37	4.43	1255	1254	ND
p-Cymene	535-77-3	16.78	0.91	1.25	1.54	1277	1270	21
cis-Linalool oxide (furanoid)	60047-17-8	21.97	0.88	1.02	3.80	1450	1441	22
Nerol oxide	1786-08-9	22.65	0.82	1.14	3.67	1474	1469	ND
trans-Linalool oxide (furanoid)	34995-77-2	22.74	0.83	1.00	4.48	1477	1469	ND
Cyclosativene	22469-52-9	23.22	0.84	2.09	2.45	1494	1487	ND
Copaene	3856-25-5	23.45	0.82	2.07	2.03	1501	1489	ND
(E)-Theaspirane	43126-22-3	23.72	0.74	1.64	2.65	1511	1500	ND
Linalool	78-70-6	24.73	0.76	0.87	4.09	1549	1544	ND
Theaspirane	36431-72-8	24.75	0.77	1.57	3.18	1549	1540	ND
β-Caryophyllene	87-44-5	26.39	0.74	1.84	2.60	1608	1598	ND
Menthol	2216-51-5	27.26	0.67	0.93	4.22	1636	1626	10
α-Terpineol	98-55-5	28.66	0.64	0.88	4.58	1681	1687	ND
α-Humulene	6753-98-6	29.02	0.61	1.68	2.95	1693	1678	ND
β-Bisabolene	495-61-4	29.65	0.64	1.55	3.36	1717	1723	65
Geranial	141-27-5	29.81	0.50	1.07	4.14	1724	1729	ND
Curcumene	644-30-4	30.79	0.61	1.35	3.33	1764	1766	ND
β-Damascenone	23726-93-4	31.90	0.59	1.14	3.80	1809	1821	9
Dihydro-β-ionone	17283-81-7	32.21	0.55	1.28	3.29	1822	1825	ND
Geraniol	106-24-1	32.30	0.58	0.79	4.60	1826	1836	ND
Geranylacetone	3796-70-1	32.67	0.56	1.20	3.59	1841	1852	ND
Neophytadiene	504-96-1	34.25	0.52	2.26	2.44	1908	1915	ND
β-Ionone	79-77-6	34.64	0.53	1.21	3.40	1925	1926	ND
5,6-Epoxy-β-ionone	23267-57-4	35.93	0.34	1.15	3.62	1982	1977	7

Supplementary Figure 1 – SF1: Pearson correlation matrix obtained from absolute response data corresponding to UT features with a F calc > 4 for all classes. Hierarchical clustering is based on Pearson correlation while heat map colorization ranges from blue (-1) to red (1) *r* values. Black squares highlight features cluster with a strong correlation. Comments are reported in the text.

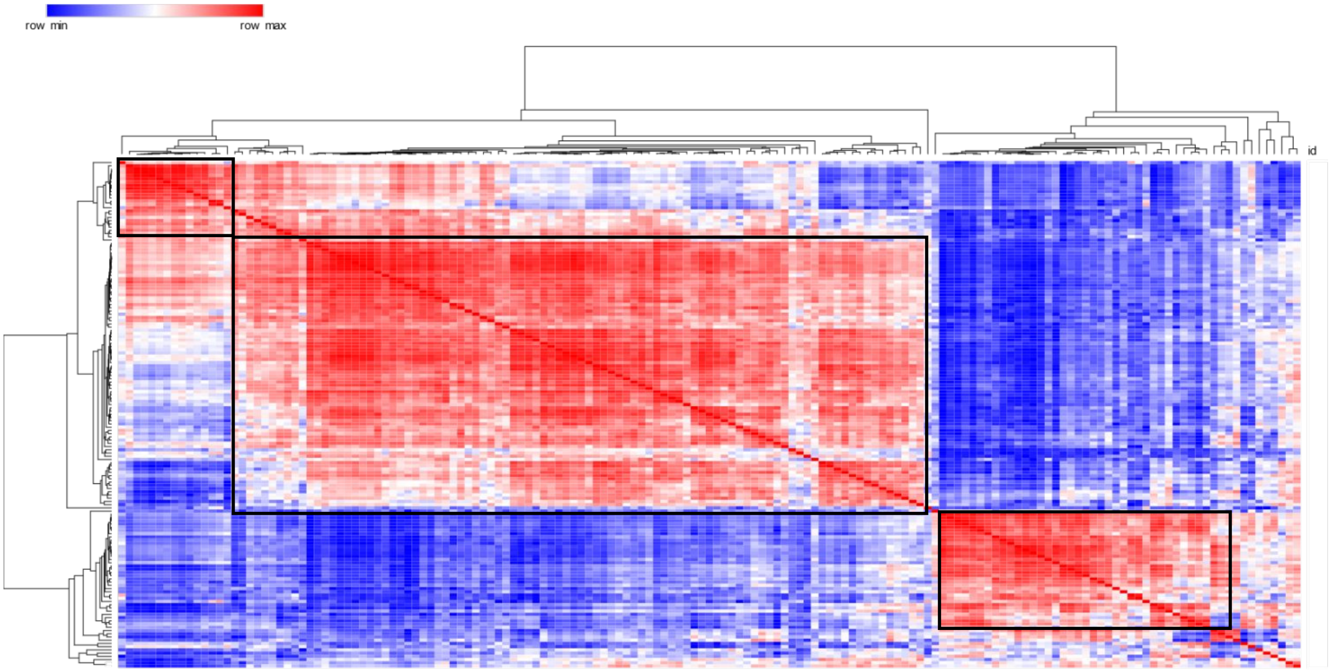

Supplementary Figure 2 – SF2: comparative visualization between composite class images obtained by summing 2D chromatograms from samples belonging to the same class. For process details refer to the section "Composite class image fingerprinting".

In **SF2A** the *analyzed* image is the composite-class chromatogram from *L. par* fermented samples while as *reference* is taken the composite class image from all herbage samples.

In **SF2B** the *analyzed* image is from *L. buc* while the *reference* is that from herbage samples.

In **SF2C** the *analyzed* is that from *L par* samples compared to the *reference* from control samples.

The comparative visualization is rendered as "colorized fuzzy ratio"; the difference at each data point between aligned pair-wise images is computed and colored green, when positive (larger detector response in the *analyzed* image) or colored red, when negative (larger detector response in the *reference* image).

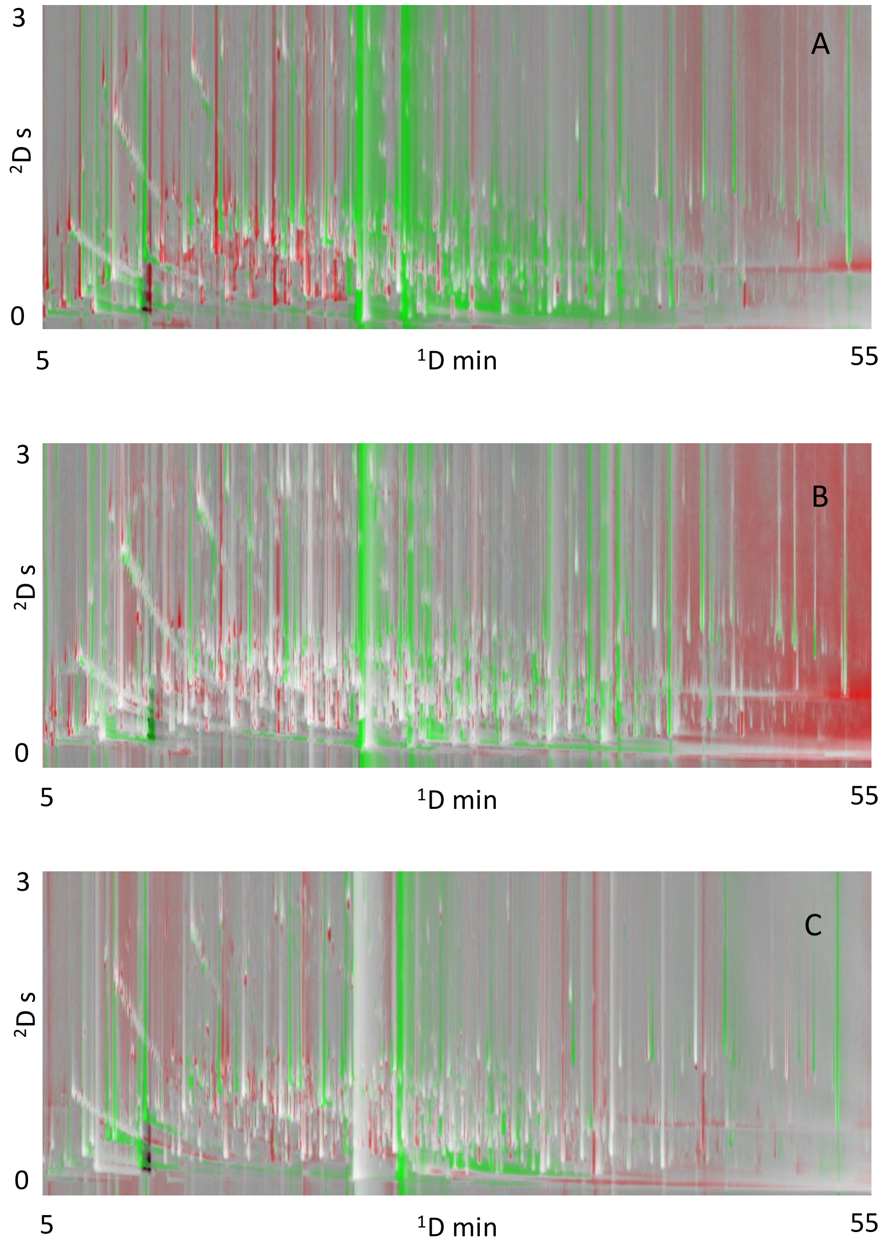

Supplement: Supplementary file 1 — jf2c03652_si_001.pdf [file jf2c03652_si_001.pdf]
